# Risk factors associated with high prevalence of intimate partner violence amongst school-going young women (aged 15–24years) in Maputo, Mozambique

**DOI:** 10.1371/journal.pone.0243304

**Published:** 2020-12-09

**Authors:** Maria Suzana Maguele, Boikhutso Tlou, Myra Taylor, Nelisiwe Khuzwayo

**Affiliations:** 1 Instituto Superior de Ciências de Saúde, ISCISA, Maputo, Mozambique; 2 School of Nursing and Public Health, Discipline of Public Health, University of KwaZulu-Natal, Durban, South Africa; 3 School of Nursing and Public Health, Discipline of Rural Health, University of KwaZulu-Natal, Durban, South Africa; University of Sao Paulo Medical School, BRAZIL

## Abstract

**Background:**

In many countries, there is evidence that intimate partner violence is prevalent among young women. This study aimed to determine the prevalence and the factors associated with intimate partner violence in young women (aged 15–24 years) attending secondary schools in Maputo, Mozambique.

**Method:**

Using a probability proportional sampling strategy, 431 participants were recruited, and the data were collected using a self-administered questionnaire. Binary and multivariate logistic regression analyses were performed to assess the association between IPV and sociodemographic and sociocultural factors. Odds ratio (OR) and 95% confidence intervals (CI) are reported.

**Results:**

Of the 413 participants, 248 (60%) (95% CI: 55.15–64.61) had experienced at least one form of IPV in their lifetime. Then, of the 293 participants who had a partner in the previous 12 months prior to the data collection, 186 (63.4%) (95% CI: 57.68–69.00) reported IPV in the 12 months prior to data collection. The psychological violence was the predominant type of violence, lifetime prevalence 230 (55.7%), and over the previous 12 months 164 (55.9%). The risk of IPV was associated with young women lacking religious commitment (AOR, 1.596, 95% CI: 1.009–2.525, p = 0.046) and if the head of the young women’s household was unemployed (AOR, 1.642 95% CI: 1.044–2.584, p = 0.032). In the bivariate analysis the odds of being abused remained lower among the younger teenage women (OR, 0.458 95% CI: 0.237–0.888, p = 0.021), and higher, among young women if the partner was employed (OR, 2.247 95% CI: 1.187–4.256, p = 0.013) and among the young women believing that males are superior to females (OR, 2.298 95% CI:1.014–5.210. p = 0.046).

**Conclusion:**

These findings reveal a high prevalence of IPV among young women. Comprehensive programs should incorporate socioeconomic empowerment strategies to increase women’s autonomy. There is a need to address religious beliefs through cultural perspectives, to improve social interactions that promote violence free relationships, gender egalitarian norms, and physical and emotional wellbeing for young women.

## Background

The World Health Organization (WHO) estimates that globally one in three women experience violence from their partners and, in the region of Sub-Saharan Africa (SSA) the statistics point to 36.6% [1]. Intimate partner violence (IPV) in SSA among ever-partnered women aged 20–24 years and among women aged 15–19 years is estimated to be 31.6% and 29.4%, respectively [1].

Globally the number of young women is increasing, with 12% of the world’s population being females aged 15−24 years [2]. Many of these young women live in developing countries, where they are affected by inequalities, such as their low level of education and high rate of unemployment [2]. Such factors affect their autonomy in making decisions about their lives, and in allowing them financial independence. This may lead them to potentially vulnerable relationships that put them at risk of violence such as IPV.

The Sustainable Development Goal 5 (SDG) emphasizes the need to address gender inequalities and the target date to achieve this is 2030. This requires that actions be taken, and programs developed over the next decade [3].

In addition to the social inequalities affecting young women, as a result of their youth there may be specific risk factors, and in particular contextual risk factors that affect young women, that could influence their vulnerability to partner violence.

Therefore, investigating such risk factors for IPV and obtaining evidence of their magnitude among young women is important to inform policymakers and programmers, in order to develop appropriately targeted interventions.

While numerous factors influencing IPV against women have been identified by social scientists, most of the studies are focusing on older women [4]. The factors most reported about women in general include individual factors such as their HIV-positive status, level of education, economic status, having witnessed violence during childhood and partner’s alcohol abuse [5–7]. Patriarchal societies have also been reported as promoting IPV against women [8–10]. For instance, in many African societies male figures occupy senior positions in their families and communities. Women are socialized to accept the seniority of their male partners and are required to comply. For instance, although according to custom a male can have more than one wife, this may expose the female partners to poor physical and emotional health [11].

The gender roles and negative community norms that influence IPV require communities to reflect and develop improved strategies for promoting norms that will be beneficial to both sexes so that IPV can be eliminated.

The continuing practice of IPV in families and communities affects the socialization of young people [12–14]. In addition and of major concern, is that young people start dating early in their lives [4, 15]. However, in most SSA countries there are no structured interventions to prepare them before they start dating, and they thus tend to imitate practices to which they have been exposed, such as IPV [4, 16–18].

Although there is increased awareness about the problem of IPV since the 2013 WHO report, providing solutions to address the problem remains a concern. According to the WHO (2020), research investigating factors underpinning IPV among young women remains of particular importance since the prevalence around the world is still escalating [19]. Coll et al. (2020) also highlight in their study the many countries where IPV is increasing, although there are exceptions in some smaller countries [20]. There is a need to develop appropriately targeted interventions, depending on the age and circumstances of the women.

Mozambique is one of the countries with a high prevalence of IPV. The most recent data report that the lifetime prevalence of IPV among women aged 15–24 years ranges between 36% and 47.8% [21]. Further, amongst young women in Mozambique the factors that were reported to influence IPV included sexual coercion, early marriage, early dating relationships, alcohol abuse and economic constraints [21–23]. Information is urgently required to suggest specific improvements to address the current dearth of intervention programs that address the needs of young women, and to provide evidence to encourage policymakers to act, in order to work towards achieving SDG 5.

Young women are more likely to provide valid responses if the questionnaire is confidential and anonymous [24]. In Mozambique prior studies were conducted more than seven years ago. To obtain more up to date and accurate information in order to promote change and reduce the occurrence of IPV, this study aimed to determine the prevalence and the risk factors for IPV in young women aged 15–24 years attending secondary schools in Maputo, Mozambique in 2019.

## Material and methods

### Study area and setting

The study was conducted in three secondary schools in KaMpfumu District Municipality, Maputo. These schools comprise the majority of the secondary schools in Maputo, enrolling students from grades 8 to 12. The KaMpfumu District Municipality is the most urban area of Maputo, covering an area of 12 square kilometres, and the total population is 80 550, of which 42 575 are females [25]. Youth in the age group 14–24 years constitute about 49% of the population. KaMpfumu District has the lowest level of poverty among the seven city districts, estimated at 28%. This metropolitan area is populated by people from different backgrounds, economic class, cultures and perspectives concerning health-seeking behavior [25]. The prevalence of IPV among school-going young women aged 15–24 years in Maputo is unknown; however, the prevalence among the general population aged 15–49 years is estimated to be 54.4% [21].

### Study design and population

This was a cross-sectional study following focus group discussions that explored the factors associated with IPV among young women in Maputo. A review of the literature indicated a lack of information about contextual factors associated with IPV among young women. In total six focus groups were held with young women (15−24 years) at schools to explore their perceptions of the risk factors associated with IPV. These young women did not participate further in the study.

In Mozambique, although education is free through 12 years of age, the female literacy rate is 28% [26]. The study population was young women 15−24 years and women attending classes in each of the selected schools during August and November 2019, aged between 15–24 years, were included in the study. Women aged 15−24 years are at increased risk of adverse health and reproductive health outcomes [5] and since young women in Mozambique often start school when they are older, 15−24 year old women are to be found in secondary schools [26, 27]. Also, since schools are often the setting where young women receive education and health promotion programs, it is feasible that comprehensive programs can be provided aimed at empowering young women with the knowledge and skills to reduce their vulnerabilities. Such interventions can contribute to changing behaviors through collaboration with the educational sector and consideration of the contextual environment when targeting youth [28].

### Study sample

The sample size calculation was based on the population-proportional size sample, using a 95% confidence interval and a 5% degree of precision. We expected 50% prevalence, and therefore added 10% to the sample for invalid responses [29]. The probability proportional random sampling strategy was employed to select the 450 participants from all the classes in the schools, based on the total number of students per academic class, per age group. The random selection provided an equal chance for all the students to be selected so that the findings could be generalized to similar populations. All the participants who mentioned that they had never been in a relationship (n = 19) were subsequently excluded from the study.

### Data collection

#### Pilot

The questionnaire was piloted in a school with a similar setting, but not included in the study, amongst 42 young women (nearly 10%), to ensure clarity of the questions and consistency in the methods of questioning and the data collection procedure. After the pilot, some issues relating to the demographic information were re-formulated for the school-going population in an urban setting in Maputo.

#### Instruments

Prior to the survey an exploratory study using focus group discussions was conducted and themes were generated to explain the IPV experienced by young women in Maputo city. The questionnaire for the survey was adapted informed by the themes and based on the WHO Multi-country surveys of violence against women instruments (Garcia-Moreno, 2005). We also based our questions on the socioecological model (Bronfenbrenner, 1998) [30]. The themes that emerged from the focus group discussions were integrated into the model for variables and included individual factors (Age of women, age of partner, young women’s relationship status, young women’s employment status; HOH education level, HOH employment status, partner employment status), community factors (religiosity) and societal factors (perceptions of gender roles).

The questionnaire used in this study to estimate the IPV and associated factors was adapted from the WHO Multi-country Survey of Women’s Health and Domestic Violence against Women (Garcia-Moreno, 2005). The WHO Multi-country tools are recommended since they cover issues of IPV and their validity and reliability have been confirmed [31]. The questionnaire’s validity in the Portuguese language was confirmed in the study done in Brazil in two different social contexts (urban and rural). The results indicated the adequacy of the instrument in estimating the occurrence of IPV and the associated factors. The study reported a Cronbach alpha coefficient of 0.88. Thus, the instrument has been shown to be reliable, consistent and adequate to be used in other similar studies accessing IPV, in different contexts such as this study [32].

The questionnaire was translated from English (S1 Appendix) to Portuguese (S2 Appendix) and back translated into English by a second translator to ensure consistency. The selection of the questions was designed to address the sociocultural context of the young women attending secondary schools in Maputo, based on the information obtained from the focus groups and the available literature.

Our study was done in an urban setting and the instrument enabled us to estimate the different types of violence (physical, sexual and psychological). The IPV was measured both across the lifetime and in the 12 months prior to conduct the survey.

*Dependent variables*. The dependent variables comprised acts of physical, sexual and psychological violence, and the questions were adapted from the WHO Multi-country Survey of Women’s Health and Domestic Violence against Women (Garcia-Moreno, 2005), using the subscales of the Abusive Behavior Inventory of physical violence [33], psychological violence [34] and sexual violence [35]. Physical, sexual or psychological violence was assessed by questioning if, since the age of 15 the young woman had ever experienced one of the acts from a current or past partner. Experience of physical, sexual or psychological violence was considered confirmed if the response was “yes” to at least one of the defined criteria questions.

Physical violence (8 questions). For example: “Has he or any other partner ever slapped you or thrown something at you which could hurt you?”

Sexual violence (10 questions). For example: “Has he or any other partner ever physically forced you to have sexual intercourse?”

Psychological abuse (13 questions). For example:” Has he or any other partner called you insulting names?”.

The questions presented to participants to assess the occurrence of each type of violence are detailed in the questionnaire (S1 Appendix).

The lifetime prevalence of IPV was defined as the proportion of women who have or ever had an intimate partner and reported violence from a partner at any time in their life since the age of 15 years.

The current prevalence or 12 months prevalence of IPV was defined as the proportion of women who currently have or ever had an intimate partner in the previous 12 months before the survey and reported violence in the previous 12 months.

An intimate partner was defined as any male partner with whom the young women have or ever had a romantic relationship that included sexual activities, either spouse/husband, boyfriend/dating partner, or ongoing sexual partner/occasional partner. The definition was based on the sociocultural context of an urban setting in Maputo. After accessing the meanings young women attribute to intimate relationships, during the pilot. Therefore, we contextualized the definition to address the specific group during the survey.

*Independent variables*. This study is part of a larger study undertaken to assess the prevalence and risk factors for IPV among young women in Maputo city. Prior to the survey an exploratory study using focus group discussions was conducted and themes were generated to explain the IPV experienced by young women in Maputo city. Therefore, the questionnaire, based on the WHO Multi-country surveys of violence against women was informed by the contextual themes and the literature concerning IPV against women. For example, the focus group discussed themes of religiosity and IPV. Therefore, we included the religiosity as a contextual variable to investigate how religion might shape young women’s perspectives, beliefs and the influence on IPV. The variables were also informed by the available literature to explain the risk factors for IPV in different sociocultural contexts. In settings such as Mozambique the society is dictated to by social norms which give privilege to male dominance [21, 23, 36]. Thus, since contextual sociocultural factors may explain the occurrence of IPV, we included sociocultural variables. We also included questions from the socioecological model which established that the risk factors for IPV emerge from different constructions within people’s interactions. These included individual, relationship, community and societal factors (Bronfenbrenner, 1998). The independent variables were divided into two sections. Section one comprised socio-demographic characteristics measured as categorical variables and section two investigated the sociocultural risk factors for IPV considering agreement or disagreement with statements of male superiority and the statements of acceptance of IPV. These were measured on a 4-point Likert scale from strongly agree, agree, disagree and strongly disagree [37].

Section one of the questionnaire comprised questions about:

Socio-demographic factors: Age, divided into two categories (15−19, 20−24 years); Employment status of respondent; Relationship status of respondent; Commitment to religion: Defined as the degree to which a participant adheres to his or her religious values, beliefs, and practices and uses them in daily living. These were measured as yes/no responses.

Household factors: With whom the participant lives; by whom the participant was raised; Educational level of the Head of Household (HOH); Employment status of the HOH.

Partner background: Age differences between the young women and partner; Alcohol consumption by partner; Employment status of the partner.

Section two comprised sociocultural variables measuring risk factors, and examples of the questions are provided:

Perceptions of gender roles (8 questions): For example: “Do you believe that a man has a superior position within a society than women?”.

Tolerance of violence (8 questions). For example: “There are times when violence by men to women is okay”.

### Ethics

This study was approved by the Humanities Social Sciences Research Ethics Committee of UKZN (HSSREC), ref: HSS/2005/018D, and the National Health Bioethics Committee of Mozambique (CNBS), ref: 360/CNBS/19. Permission was obtained from the National Directorate of Education in Maputo and the directorates of the selected schools. Consent forms and assent forms were explained and distributed to all participants before the study. All participants provided written informed consent voluntarily. Participants under 18 years of age provided assent and the consent from their parents or guardians. After they returned signed consent forms, they were eligible to participate in the study. No monetary reimbursement was given to them for their participation. Anonymity and confidentiality were ensured, and the participants’ names were not written on any questionnaires. Privacy was maintained by keeping the participants separated from each other during the completion of the self-administered questionnaire. They were told that their participation was voluntary and that they had the right to terminate it and they were assured that they would not be affected in any way if they decided to do this. There was minimal risk that the study had the potential to bring back negative memories, but participants were told to inform the researchers if this occurred, and they were assured of the availability of referral services. Participants benefited from the information about IPV and how to prevent IPV that was provided after the survey. The researcher made available contacts for reference services for assistance in case participants needed help if they experience violence. Those participants who contacted the researcher after the survey who reported experiencing IPV, were provided with the names and contact details of the services providing assistance to women suffering from gender-based violence and IPV.

### Data analysis

The Hosmer and Lemeshow test of the goodness of fit suggested the model was a good fit to the data as p = 0.396 (> 0.05). The chi-square statistic on which it is based is very dependent on sample size, so the value cannot be interpreted in isolation from the sample size. However, we had a powered sample and our data also met the one in ten rules of thumb of ten outcome events per predictor variable in the logistic regression.

Data were analyzed using SPSS version 25.0 computer software after assessing its completeness. Proportions were used to estimate the lifetime and current or 12 months prevalence of IPV among young women. For lifetime IPV the denominator included all women who currently have or ever had an intimate partner and reported ever experiencing IPV at any time in their life. For 12 months IPV the denominator included all women who currently have or ever had an intimate partner in the 12 months prior to survey and reported experiencing IPV in the 12 months prior to survey [31, 33].

Logistic regression was used to identify risk factors associated with IPV, and odds ratios (OR) and 95% confidence intervals (CI) are reported. After conducting the bivariate analysis of IPV and the potential risk factors, significant risk factors, were then included in the multivariable logistic regression. A p value <0.05 was deemed statistically significant.

## Results

### Sample description

Overall, 450 young women were enrolled in the study, but only 431 were included in the analysis. The age of participants ranged between 15 and 24 years, where the mean age was 18 (SD 1.514). Most of them 368 (85.4%), were in the 15−19 age group. Of the respondents, 226 (52.4%) had completed grade 10, 399 (98%) were unemployed, 259 (61.7.%) were committed to religion, and 286 (66.4%) were in a dating relationship at the time of data collection. Over half of the young women’s HOHs 238 (56%) were employed. Of the young women’s partners, 311 (72.2%) were not alcohol users, 366 (85.3%) were unemployed, and 194 (45.8%) were younger than or the same age as the young women. The socio-demographic characteristics of the study participants are presented in Table 1.

**Table 1:** Socio-demographic characteristics of young women.

Age categories (years)	Frequency	Percent (%)
Mean: 18 (1.514)	n = 431	
15–19	368	85.4
20–24	63	14.6
**Education level (n = 431)**		
Grade 10	226	52.4
Grade 11	205	47.6
**Religiosity (n = 420)**		
Committed to religion	259	61.7
Not committed to religion	161	38.4
**Status of employment (n = 407)**		
Employed	8	2
Unemployed	399	98
**Status of relationship (n = 431)**		
Currently married	7	1.6
Currently in relationship/dating	286	66.4
Currently not in a relationship but have had previously	138	32
**Head of household status of employment (n = 425)**		
Employed	238	56
Unemployed	187	44
**Partner alcohol use (n = 431)**		
Partner alcohol user	120	27.8
Partner not alcohol user	311	72.2
**Partner status of employment (n = 429)**		
Employed	63	14.7
Unemployed	366	85.3
**Partner age difference (n = 424)**		
Less than ten years older	188	44.3
More than ten years older	16	3.8
Younger/same age	194	45.8
Do not know	26	6.1

### Prevalence of IPV

#### Lifetime prevalence of IPV

Of the 413 young women who provided information about their experiences of IPV, 248 (60%) (95% CI: 55.15–64.61) had experienced at least one form of IPV in their lifetime. More than half of the young women, 230 (55.7%), had experienced at least one act of psychological violence, 120 (29.1%) had experienced at least one act of sexual violence, and 93 (22.5%) had experienced at least one act of physical violence. Of the study participants 45 (10.9%) had experienced all three forms of violence, with co-occurrence of two forms of violence reported by 55 (13.3%) for psychological and sexual violence, 32 (7.4%) for psychological and physical violence and 5 (1.2%) for sexual and physical violence. There were 361 women in the 15−19 years age category and 209 of them had experienced IPV constituting a proportion of 57.9%, whereas of the 52 women in the 20−24 age group, 39 of them experienced IPV constituting a proportion of 75.0% of that age group. In general, the overall IPV increased with increasing age, with psychological violence predominating in both age categories.

#### 12 months prevalence of IPV

Of the 293 young women who had a partner in the previous 12 months, 186 (63.4%) (95% CI: 57.68–69.00) reported IPV in the 12 months prior to the data collection, with psychological violence predominant, reported by 164 (55.9%) respondents (Table 2). Physical violence was reported by 55 (18.7%) respondents, and the prevalence of sexual violence was higher at 71 (24.2%).

**Table 2:** Lifetime and previous 12 months prevalence of physical, sexual and psychological violence by age categories.

**Lifetime IPV**	**Prevalence**		**Age categories (n = 413)**	
Overall	Frequency	Percent	15−19 (n = 361)	20−24 (n = 52)
	248	60	209 (57.9%)	39 (75%)
Physical	93	22.5	72 (20%)	21 (40.4%)
Sexual	120	29.1%	91 (25%)	29 (55.8%)
Psychological	230	55.7%	191 (53%)	39 (75%)
**12 months IPV**	**Prevalence**		**Age categories (n = 293)**	
Overall IPV	Frequency	Percent	15−19 (n = 238)	20−24 (n = 55)
	186	63.5	155 (65%)	31 (56.4%)
Physical	55	18.7	44 (18.5%)	11 (20%)
Sexual	71	24.2	53 (22.3%)	18 (32.7%)
Psychological	164	55.9	137 (57.6%)	27 (49.1%)

As expected, the overall 12 months prevalence of IPV decreased with increasing age. In general, the younger women had a higher prevalence of current IPV as compared with the older age category. There were 238 women in the 15−19 years age category and 155 (65%) of them had experienced IPV whereas of the 55 women in the 20−24 age group, 31 (56.4%) of them experienced IPV. However, some variations in the pattern suggest that although the older women are more protected from psychological violence, they are likely to be more at risk of physical and sexual violence as compared to younger women.

The lifetime and previous 12 months prevalence of IPV are presented in Table 2.

### Factors associated with IPV

In the bivariate analysis the odds of experiencing IPV were significantly lower among younger women, (OR, 0.458, 95% CI: 0.237−0.888, p = 0.021), compared to those in the older age categories. The odds ratio of experiencing IPV were significantly higher among young women who were not committed to religion (OR, 1.591, 95% CI: 1.048–2.415, p = 0.029), and young women whose HOH was unemployed (OR, 1.562, 95% CI: 1.021–2.392, p = 0.04). The risk of IPV doubled for the young women whose partners were employed (OR, 2.247, 95% CI:1.187–4.256, p = 0.013). For those whose partners were more than ten years older, the risk of IPV appeared to be more than fourfold higher (OR, 4.5283, 95% CI: 0.986–20.805, p = 0.052). A strong trend associated with IPV was also seen for young women who agreed with one statement of male superiority (OR, 2.298, 95% CI: 1.014–5.210, p = 0.046). The other sociocultural variables were not found to be significantly associated with young women’s experience of IPV.

The multivariable analysis confirmed the association between IPV and the young women’s lack commitment with religion (AOR, 1.596, 95% CI: 1.009–2.525, p = 0.046) and with young women’s HOH unemployment status (AOR, 1.642, 95% CI: 1.044–2.584, p = 0.032). There were also trends that the risk of IPV increased if the young women’s partner was more than ten years older, (AOR, 3.183, 95% CI: 0.661–15.341, p = 0.149) and if he was employed (AOR, 1.675, 95% CI: 1.044–2.584, p = 0.155). Similar trends indicated lower odds of IPV among the younger women (AOR, 0.750, 95% CI: 0.365–1.541, p = 0.434) as compared to the older women and also lower among young women who agreed with one statement of male superiority, (AOR, 0.894, 95% CI: 0.524–1.525, p = 0.682). The logistic regression analysis results are presented in Table 3.

**Table 3:** Logistic regression analysis of demographic and sociocultural variables: IPV dependent variable: Ever-experienced IPV.

		Bivariate			Multivariable		
Predictor variables	Categories of Predictor variables	OR	95% CI	P-value	AOR	95% CI	P-value
Age of women	15–19	0.458	0.237−0.888	0.021	0.750	0.365–1.541	0.434
	20–24	Ref			Ref		
Religion commitment	No	1.591	1.048–2.415	0.029	1.596	1.009–2.525	0.046*
	Yes	Ref			Ref		
Employment status of the head of household	Unemployed	1.562	1.021–2.392	0.04	1.642	1.044–2.584	0.032*
	Employed	Ref			Ref		
Employment status of partner	Yes	2.247	1.187–4.256	0.013	1.675	1.044–2.584	0.155
	Do not know	0.179	0.037–0.854	0.031	0.302	0.031–2.908	0.300
	No	Ref			Ref		
Age difference of the partner	Less than ten years older	1.438	0.944–2.190	0.091	1.384	0.868–2.207	0.172
	More than ten years older	4.528	0.986−20.805	0.052	3.183	0.661–15.341	0.149
	Do not know	0.278	0.111–0.693	0.006	0.360	0.114–1.139	0.082
	Younger or same age	Ref			Ref		
Do you believe that a man has a superior position within a society than women	Agree	2.298	1.014–5.210	0.046	0.894	0.524–1.525	0.682
	Disagree	Ref					

* = Statistically significant in multivariable logistic regression (AOR).

## Discussion

The results of this study provide new information about young women attending Maputo secondary schools regarding the prevalence of IPV and the factors placing them at risk. Mozambique, as with many Sub-Saharan African countries has had a difficult history and experienced much violence in the 20^th^ century. The high prevalence of IPV reported by these young women, more than half of whom had experienced IPV, emphasizes the need for targeted interventions. The factors associated with IPV emphasize the importance of changing social norms away from the acceptance of IPV and perceptions of male superiority, and to develop a more gender equitable society that values the contribution of young women, the target of SDG 5. Since women constitute 51.4% of the Mozambique population [38], it is critical that the state provides economic opportunities that allow young women to be independent and to fulfil their potential. Targeting young women who are attending school would appear to be a feasible initial strategy.

Previous studies undertaken in Mozambique include the National Demographic Survey which had a section on domestic violence among the general population [21], studies undertaken on clinical samples in healthcare centres [39, 40] and a study targeting universities and secondary school students, between the ages of 15 and 45 years. This latter study measured 12 months’ prevalence of IPV and lifetime prevalence of non-partner violence, that is, violence from other sources [22].

It may be difficult to compare the prevalence data on IPV because previous studies used different methods. For example, the national and clinical surveys considered women at risk as only ever married/cohabiting women of reproductive age. The study among university students considered currently partnered women. In our study we considered all women who ever had an intimate partner from the age of 15 (Garcia-Moreno, 2005) and we included all those who currently have or those currently not having, but who ever had an intimate partner.

These previous studies did not provide clarity regarding the prevalence and the factors underpinning IPV in the specific group of secondary school-going young women (aged 15–24) years in Maputo. As the capital city of Mozambique, Maputo draws migrants from many other parts of the country and is an appropriate place to initiate targeted programs in schools to reduce IPV, in order to work towards SDG 5 against gender violence.

### Prevalence of IPV among young women

In our study, the lifetime prevalence of IPV of 60% among young women was higher than that reported in previous studies in Mozambique, which ranged from 36% to 47.8% [21]. It was however similar to that reported in studies across SSA countries among women aged 15 to 24, where the lifetime prevalence of IPV ranged from 19% to 66% [41]. The prevalence in our study is slightly higher than the findings from a recent study that explored the trends in prevalence and risk factors associated with IPV among Zimbabwean women of reproductive age, where the prevalence among women aged 15−24 years ranged between 43% and 48%. The prevalence is also higher than findings from the United States of America (USA), where the prevalence ranged from 8% to 51.2% [42–45].

The 12 months' prevalence of IPV reported in our study of 63.5% is similar to a previous study in Mozambique among universities and secondary school women, where the 12 months prevalence was reported at 63.2% [22]. The 12 month prevalence reported in our study is higher than that reported in studies from SSA and elsewhere, which ranged from 7% to 57%, where for example in Serbia, IPV prevalence was 7%, and in Ethiopia, where the reported prevalence was 57% [41]. Similarly, the prevalence reported in our study is higher than reports in a recent study conducted in Low and Middle-Income Countries, where the range was between 34.4% to 46% [20]. Comparisons between our study and other studies are challenging due to the different measures used for current IPV and the different time periods. In South Africa, a study involving 2 115 grade 8 learners, which measured IPV prevalence over 12 months using an interview administered questionnaire, reported the prevalence at 30.9% [46]. Similarly, in a study from Tanzania among a sample of 226 young men and women, amongst the young women aged 15–30 years the overall prevalence of IPV measured in the previous 12 months was 35.8% [13]. In another study conducted in South Africa, the data regarding the prevalence of IPV over three months, were collected through self-completed interviews among a sample of grade 8 students, and the prevalence was 39.1% [47].

The study involving ten countries from the WHO Multi-country Study on Women's Health and Domestic Violence against Women, used face to face interviews as the method of collecting data in urban and rural sites [41], and under reporting of IPV was possible due to this method of data collection. In contrast, the use of self-administered questionnaires completed in an environment that provided privacy using self-reports and ensuring confidentiality and anonymity in our study, may have contributed to the participants feeling able to disclose their experiences of IPV. Therefore, the high prevalence found may be associated with the comprehensive questionnaire, training, and robust method used in collecting the data for this study.

In our study, psychological violence was the most reported form of IPV, with a prevalence of 55.7% for lifetime IPV and the prevalence of 55.9% for 12 months prevalence. Our findings are in concordance with other studies which assessed the occurrence of the three forms of IPV [21, 22]. In studies from SSA and elsewhere including the Zimbabwean study [13, 20, 41, 48] and from USA settings [44, 45, 49], psychological violence was reported as the most prevalent form of IPV. This was also found in the study conducted among grade 8 learners in an urban area of South Africa, where psychological violence was the most reported finding [46]. In contrast, another study’s findings from South Africa among adolescent learners revealed that the participants experienced more physical than psychological violence [47]. The difference in the form of IPV found in a study conducted in South Africa was deemed to be the result of the attitudes of the young women disagreeing with the ideologies of male dominance, which reduced the women’s risk of being emotionally abused. However, the authors’ findings suggested that these attitudes of disagreement might increase the risk of physical violence, since this could possibly increase their relationship conflicts. Therefore, for those partners who use physical force to solve problems, this might be a reason to increase the use of physical violence [46, 47].

In this study the lifetime prevalence increased with increasing age and this was consistent with findings from the National Demographic and Health Survey in 2011 in Mozambique. The lower prevalence was reported among the younger age category 15−19 years (36.7%) and the higher prevalence among the older age categories 20−24 (47.8%) [21]. This was similar in a study done in SSA and elsewhere [41] and in a Zimbabwean Study [48]. However, as expected, the current prevalence of IPV decreased with increasing age. In general, the women in the youngest age category had higher current IPV prevalence as compared to the older age category. This may suggest that violence occurs early when young women enter or initiate relationship. Similar trends were observed in previous studies investigating 12 months’ IPV and the youngest age women reported more IPV as compared to the older ages above 24 years [22]. This is also consistent with findings from a study conducted in Low-Middle-Income countries, including countries such as Rwanda, Namibia and Senegal [20], and in the study from SSA and elsewhere [41] where the higher prevalence of current IPV was observed among adolescents and younger adults of 15−24 years. The Spanish Macro surveys study of ever-partnered women reported higher prevalence of current IPV in women between 16−29 years [50]. However, some variations in some settings were reported. For example, in Burkina Faso, Kenya, and Ethiopia and also in some settings in Europe and Central Asia, the current IPV prevalence was skewed among older ages as compared to younger groups between 15−24 years [20, 41]. The findings from South African studies, suggest that the young women’s inexperience about relationships may limit their ability to use nonviolent methods when in conflict and to prevent violence [46, 47].

Possible explanations of the varying IPV prevalence may however be due to the different sample sizes, and the age categories. Our study recruited only school-going women between the ages of 15 and 24, whereas other studies recruited women between the ages of 15 and 49.

### Factors associated with lifetime IPV

The significant factors associated with IPV are reported here in terms of the Socioecological model (Bronfenbrenner, 1998) and include individual factors (age of women, age of partner, HOH financial status, partner financial status), community factors (religiosity) and societal factors (beliefs about male superiority).

#### Individual factors

*Age of young women*. Our study revealed that in the bivariate analysis, the odds of being abused were lower among younger women (15–19), (OR, 0.458, 95% CI: 0.237−0.888, p = 0.021), as compared to those older (20–24) years. These findings were consistent with previous studies which reported increasing odds of IPV with increasing age among groups of young women in studies from SSA and elsewhere, and in the recent study conducted in Zimbabwe to assess the trends in IPV prevalence among ever-partnered women [21, 41, 48].

These results suggest that women in their early twenties may be more time exposed to relationship conflicts since they may be married or in committed relationships. They are perhaps more likely to face relationship conflicts and therefore to tolerate IPV and to remain in a relationship with violence, while the younger women are probably at the initiation stage of their relationships, and are therefore less time exposed to relationships and conflicting relationships, and may therefore be less likely to be committed to staying in such a relationship when there are conflicts.

Our multivariable analysis did not confirm the results of the bivariate analysis, but the high prevalence of IPV that was reported by the young women emphasizes the need for specific intervention strategies to address potential risk factors and to prevent the occurrence of IPV early in young women’s lives. For example, in Mozambique, a previous study among women aged 15–49 years attending schools and universities, revealed that adolescent and young women were more vulnerable to violence compared to women above the age of 24. The study’s findings also reported a positive association between their young age, their single status, their habit of going out to parties and their alcohol consumption, with increased risk of IPV [22]. In the context of South African and USA settings for example, the findings suggested that the young age of women reduced their ability to deal with the complexities and the dynamics of relationships, therefore increasing their risk for IPV [43, 45–47, 51]. In a study which explored the trends in prevalence and risk factors associated with IPV among Zimbabwean women of reproductive age (15–49 years), the older women above 40 years, were less likely to experience sexual and physical IPV compared to the younger women between 15–24 years [48]. This suggests that although the older women are more time exposed to relationships and may have experienced violence in the initial stages of their relationships, their experience regarding relationships may increase their ability to manage a more stable relationship and also to use more protective methods when in a conflicting relationship.

The likely reasons for the differences in the different studies’ results concerning the age of the women experiencing IPV, may be due to the selection process for the study samples and the comparisons between different age categories. Our study recruited only school-going women between the ages of 15 and 24 years. In Mozambique, a developing country, children often enter school when they are older than is typical in other countries and continue to attend school above the age of 20. Therefore, amongst these young women attending school to improve their educational attainment, their young age and their single status suggest that they may be less exposed to conflicting relationships, or that they can challenge and not tolerate conflict in relationships.

Further, the older age category in the study is less represented in the sample size and in this setting and this may have affected the results. Interpreting these results calls for caution and the consideration of the local context when developing and implementing IPV programs. The findings of this study suggest that the consideration of age as a factor associated with vulnerability to IPV, needs to be considered as a specific or contextual factor with potential variability across countries and settings.

These study results indicate that IPV is of concern in this urban setting in Maputo and emphasize the importance of early interventions in Mozambique, so that young women are made aware of the risk of IPV and are advised about how this could be prevented, and if violence occurs, how this should be handled. Efforts are required in order to enhance the young women’s reproductive rights, so that young women in schools and communities can pursue healthy relationships free from violence.

*Young women’s HOH’s unemployment status*, *partner employment status and partner more than ten years older*. In the bivariate analysis, several of the risk factors which have been reported from other studies showed a similar trend in this study. Young women whose partners were more than ten years older than themselves and those who did not know the age of their partner were more likely to report IPV (OR, 4.528, 95% CI: 0.986–20.805, p = 0.052).

Similar findings are reported from a Botswana study, which revealed that large age differences between partners is a predictor for IPV [52]. Moreover, studies from South Africa and other SSA settings reported that women who have partners older than themselves struggle to air their opinions about relationships, and furthermore, the older partner may expose young women to risky behavior that includes violence [41, 46, 51].

Economic status has also been shown to be an essential factor associated with IPV. In the multivariable analysis, there was significant association between the financial situation of the HOH, if unemployed, and young women reporting IPV (AOR, 1.642 95% CI: 1.044–2.584, p = 0.032). This suggests that young women from households with unemployed HOHs have an increased likelihood of experiencing financial constraints, and therefore dating older men may result from the economic dependence of women.

Moreover, the bivariate analysis suggests that having a partner who is employed (OR, 2.247 95% CI: 1.87−4.256, p = 0.013) might lead them to tolerate and to remain in relationships with IPV, since employed partners are more likely to exert financial power over the women, and may even perpetrate violence against them. This was also a confirmed trend in the multivariable analysis which showed that the risk of abuse increased when the partner is employed (AOR, 1.675 95% CI: 1.044–2.584. p = 0.155).

Our findings follow previous studies which reported that adolescent, single young adults experiencing financial strain were more vulnerable to being abused [22]. This is also consistent with findings from South African adolescent learners that revealed a high risk of IPV among those adolescents not receiving pocket money from their parents, compared to those who were [46]. Findings on low socio-economic status and the increasing risk of IPV were also reported in studies from SSA and elsewhere [41] and in a Zimbabwean and Ghanaian Studies [48, 53].

Similarly, a survey conducted in 31 countries, including South Africa and Tanzania, evaluating IPV and economic status among college students, revealed that the higher levels of IPV were associated with an inability to meet daily financial needs and with being younger [54]. In a study conducted in low-income and middle-income countries, the findings indicated that in general, richer and more empowered women reported less IPV [20]. A study conducted in South Africa found that IPV reduced with interventions combining education and economic empowerments. The violence experienced by women with their own income decreased by 55% (A0R = 0.45; 95% CI = 0.23, 0.91). There were also improvements in the women’s ability to challenge the acceptance of violence and to leave violent relationship [55]. The results suggest that improving the socio-economic circumstances of women may improve women’s independence and control over their lives, improving their skills to manage healthy relationships with no violence. Particularly among younger women, who are at the initiation stage of their relationship, their financial dependence might reduce their likelihood of leaving a relationship if there is violence.

Since many young women in schools are not working, the opportunity to remain at school may mean that they cannot leave the relationship. Young women are likely to date partners who are able to buy food, clothes, and other things. Further, young women under such conditions accept the violence that is perpetrated by their partners and this includes having sex when they do not want to and performing other sexual activities. Thus, the young women’s reliance on partners, exposes them to a higher risk of IPV (Da Cruz et al., 2014). This understanding emphasizes the public health burden of IPV and its rationale among young women experiencing financial constraints in a developing country such as Mozambique. There is indication of an initiative aimed at improving the economic and financial status of women in Mozambique, through promotion of entrepreneurship and employment opportunities, as an element of the 2030 Sustainable Development Agenda. However, the reported “National Program for women’s economic empower” was only launched recently in 2019, and it prioritizes the illiterate women from rural areas and is not addressing the needs of school going young women [56]. Notwithstanding this, there is a decade in which progress can be made towards the SDGs in Mozambique, and to develop targeted strategies towards the 2030 goals. The findings of this study contribute evidence-based information to advocate for specific prevention programs to improve the economic circumstances of young women in schools.

#### Community factors

*Religiosity*. Our multivariable analysis confirmed that the odds of experiencing IPV increased substantially among women who do not consider themselves committed to any religion, in contrast to their counterparts (AOR, 1.596, 95% CI: 1.009–2.525, p = 0.046). Being a person who follows religious principles suggests that these women were less likely to be involved in risky behavior, which can lead to IPV, or may suggest that they are more likely to use nonviolent methods when in conflict situations. In contrast to these results, a qualitative study among Togolese women reported a high tolerance of IPV among those who were committed to religion [57]. This could be because in some religion, women are supposed to respect and obey their partner at all times, and IPV may be acceptable according to such religious beliefs. For example, the findings exploring the links between religious affiliation and IPV among women in Ghana, reported trends in sexual and emotional IPV among women adhering to a religion, when compared to those not involved with any religion affiliation. However, they were at the same time less likely to report physical violence [53]. However, in the Mozambican context, there are many different churches and people can freely choose to attend whichever they prefer. Therefore, people who commit themselves to religious principles are less likely to be involved in risky behavior, including sexual and physical violence. However, they may at the same time, be more likely to be tolerant of violent behavior if their religion enforces gender inequalities and ideas about male dominance and female submissiveness [58]. Hence, religiosity may even hinder the women recognizing their risk and reporting the abuse if the religion’s views accept violence and this may perpetuate IPV [59]. The differences reported in our results may be due the methods we used to assess religiosity. We assessed religious commitment as a yes/no responses, which could have been too crude to provide sufficient information regarding religiosity as a risk or protective factor for IPV. The role of religiosity and its relationship to IPV in this setting appears to be complex and calls for further research on how religious values might shape social attitudes and perspectives on IPV. The various religions could also have a role in preventing IPV, through their social and cultural perspectives.

Through collaboration with community members, leaders and the educational sectors, IPV awareness and preventions strategies could be discussed and implemented taking into consideration both the needs of individuals and the community’s religious values and the detrimental consequences of IPV on women and children.

#### Societal factors

*Young women’s beliefs about male superiority*. In many traditional societies beliefs about male superiority remain strong, and the empowerment of women has proved to be a slow process (Shamu et al. 2015). Amongst the young women in our study despite their attending secondary school, in the bivariate analysis, a statistically significant association was found between experiencing IPV (OR 2.298, 95% CI: 1.014–5.210 p = 0.046) and their believing that “males are superior to females”. The possibility of changing such beliefs is feasible since our results differ from studies done among adolescent learners in South Africa. Young women there reported empowered attitudes, disagreeing with male superiority, and these attitudes were associated with a reduced risk of being emotionally abused [46, 47]. This reduction in abuse can be interpreted either as an indication that such women may obey their partner and not argue, or it may be hypothesized that young women who disagree with ideologies of male superiority are more likely to manage conflicts by resolving them within the relationship. In these studies, although the attitudes of women who disagree with ideas of male superiority reduced their risk of emotional violence, they remained at risk of physical violence, emphasizing the importance of including both sexes in school-based and community-based initiatives to reduce gender violence. Of concern is that both the female and the male learners in a South African studies, advanced ideologies of entitlement and male superiority [46, 47]. These perceptions are very traditional and are found in many cultures. Such beliefs need to be challenged in order to achieve more egalitarian societies which respect and develop the contribution of women, who often constitute half the population. Such an approach is essential to reduce IPV and achieve the SDG 5.

Consistency regarding the association between the sociocultural factors and the risk of IPV has also been reported from SSA and elsewhere [41, 54] and in studies conducted in different countries in low-middle income countries, where empowered women were less likely to report IPV [20].

Many statements regarding gender issues were not found to be associated with IPV in this study. Further, the multivariable logistic regression analyses did not confirm the trends of the bivariate analysis. The likely reason for these results could be the setting where the study was undertaken, since schools are often where young women receive education and health promotion programs and this population is possibly more aware of the required social norms and gender issues.

The current prevention programs on partner violence in Mozambique indicate collaboration with the educational sectors to promote gender equality [60]. However, the findings from our study revealed a surprising and concerning high prevalence of IPV and this appears to indicate a gap among young women in schools, regarding their skills to challenge male dominance norms and to effectively prevent IPV. Further, obtaining information regarding health promotion and how to prevent risky behaviors and violence, proved to be difficult among the school going women in our study, as they explained after answering the questionnaire. This suggests that the existing sociocultural vulnerabilities among young women may result from the social context or environment where they are integrated, such as the community where they live, which may endorse strong ideologies of male dominance, rather than the educational setting. In Mozambique, concurrent gender norms which privilege male dominance over women still exist. For example, in some cultures, it is perceived as normal and acceptable for men to have more than one partner [36, 61]. These findings were consistent with those of a previous study evaluating the multisectoral response of gender-based violence (GBV) in Mozambique, which revealed that young women justified that a man has a right to have sex and that women should satisfy men at any time [23]. Such views are also consistent with studies done in similar settings [13, 20, 48, 53, 54]. Similarly, social norms of male dominance and the risk of IPV against women were also described in recent studies from Bangladesh [10], Vietnam [9] and Nepal [8].

The finding from our study raise concern about the young women’s low gender empowerment attitudes and indicate an urgent need for the inclusion of education on gender equality early in the school programs and the importance of involving both sexes for effective results. Further a systematic review of studies conducted to evaluate preventive interventions on violence against adolescents and young girls in Lower Income Countries, including the SSA countries, pointed out that the persistent sociocultural factors may limit the success of prevention programs in these settings [4, 62].

There is thus a need for consideration of the contextual sociocultural environment and to include comprehensive programs that empower younger women with skills to challenge such negative gender norms when implementing IPV programs in schools. Comprehensive programs require collaboration with the community and the educational sector in working to change gender norms regarding male dominance. This can further improve social interactions and relationships and thus prevent violence and IPV.

### Implications

This study has identified important factors associated with IPV and emphasizes the need to develop targeted programs to reduce IPV. Using the information obtained from this study interventions can be developed based on the data and which offer opportunities to work with young women attending secondary schools, to determine whether programs that address the protective variables are associated with reductions in IPV rates in young women. This cross-sectional research improved our understanding of the magnitude of IPV among young women and some of the contextual factors that need to be addressed. It has thus contributed information towards the development of the contextual preventive strategies required, since the study provided new insights and empirical data that can be used in developing programs to reduce IPV in the young women. The study has shown that targeted interventions should be initiated in secondary schools and evaluated. Based on these results the targeting of the interventions will need to be further developed and extended to young women not in school and to young women living in rural areas. In working to contribute to SDG 5 this cross-sectional study provides useful current information about the extent of the problem and makes recommendations as to how IPV among young Mozambican women can be reduced.

### Limitations and strengths

As with most studies, this study had limitations. Firstly, as the study was based on young women’s self-reported experiences of IPV, it is possible that some may have under- or over-reported their experiences, according to the perceptions that they attribute to IPV. The time factor is a further limitation, in asking them to think back over a year and over their lifetime. Secondly, the study is a cross-sectional design, which could not establish firm causal relationships. Longitudinal studies, such as intervention studies, would be the next step and are needed to determine whether the protective variables identified are associated with reductions in IPV rates. Thirdly, the measurement of certain variables (e.g. alcohol use, the status of employment, religiosity) in a yes/no format may have been too crude to capture their association with IPV fully. Fourthly, data were collected only among secondary school-going young women in KaMpfumu District Municipality and thus excluded those who were not in school or those who had dropped out of school. This may limit the generalizability of the findings to all young women in Maputo. We did not find statistically significant results in most of our multivariable results, which could be due to the sample size power limitation, and we are aware that the relationships observed in our sample may not be found in all the population we intend to represent. Thus, this could limit the effectiveness of an intervention based only on the sample instead of the whole population. Further in this study we did not ask about childhood experiences of violence or previous exposure in their households to witnessing violence. The study benefited from the population proportional sample size, the random sampling method, the use of standardized, validated questionnaires and of stratified and multivariable methods of analyzing the results to reduce the effects of confounding factors. More studies with larger samples including specific variables affecting young women such as peer and parental influences, childhood experiences of violence, are needed to provide the basis for inference to the entire population, but this study provides a basis for the development of targeted interventions for young school going women.

Despite weaknesses, the strength of this study is its further confirmation of the findings from previous studies. The results from this study also provide new insights to contribute useful empirical data for programs which address interventions on IPV, especially for targeting young women in order to design better intervention and prevention measures.

## Conclusions

We found a high prevalence of IPV in our study and report that psychological violence was the most prevalent form of IPV in all the age categories, followed by sexual and physical violence for both lifetime and twelve months’ prevalence. Younger women (teens) were less likely to be abused compared to those older (20−24 years). IPV was highly associated with the low economic status of women, indicated if the HOH was unemployed. The odds of IPV increased among women whose partner was employed and there were trends of IPV if the partner was much older. The results also indicate an association between IPV and sociocultural factors resulting from the young women’s lack commitment to religion and trends indicating increased odds of IPV among women agreeing with ideas about male superiority. The results from this study have identified the contextual socioecological factors interacting at individual, community and societal levels. The study emphasizes the need to develop and implement interventions early in the lives of young women attending schools and the consideration of a multilevel approach to address the socio-economic and cultural risk factors which emerged is important to include in such programs.

Although research linking the economic aspects with IPV is evident among the adult population, this study examined contextual factors among the specific group of school going young women in an urban setting. The study is an important step contributing to the field of IPV, by providing an understanding of the contextual risk factors, their role and how the interaction at individual, community and societal levels place young women at high risk of IPV in Maputo city.

The study findings are also noteworthy as an important contribution to the body of literature, as it investigated risk factors and also incorporated sociocultural factors influencing the experiences of IPV among schooling young women aged 15−24 in Mozambique, not previously studied. The understanding of the cultural context may help to explain the occurrence of IPV and the responses needed, since in this setting, the majority of communities are dictated to by the social norms which give privilege to men’s dominance over women, leading to gender inequalities and promoting IPV. This study is also important, given its focus on young women, who are a group that is affected by gender inequalities which result in an increased risk for IPV. Research concerning risk factors for IPV among young women aged 15−24 are standard in the setting of USA but less so in SSA, and this has not previously been undertaken in Mozambique. This study thus increases the current information by providing a unique context of sociocultural gaps which place young women at increased risk of IPV in the study setting.

In particular, the economic vulnerabilities within young women’s families have important political implications and consideration of programs that bolster financial capital within households and communities, while integrating cultural beliefs and gender egalitarian notions. This study’s findings provide the information required to enhance the existing programs in schools in Mozambique and emphasized the need to incorporate in such programs, strategies of women’s economic empowerment and consideration of religious and cultural values.

Mozambique is committed to the 2030 targets for sustainable development, and the 2015−2019 and the 2020−2024 Government five-year programs, have integrated the strategic objective of gender equality and empowering women and girls [63]. Therefore, the study contributes to Mozambique’s efforts toward achieving SDG 5, by providing the evidence-based information required to advocate for improvements in existing programs. The study findings call for involvement of all stakeholders and the need for consideration of the cultural and social dynamics and their implications, when addressing gender-based prevention programs. An integrated approach to ensure that no woman is left behind is required to end IPV among young women in Mozambique and contribute to the success of the 2030 SDG5.

## Supporting information

S1 Appendix English questionnaire. File with English questionnaire.
(PDF)

S2 Appendix Portuguese questionnaire. File with Portuguese questionnaire.
(PDF)

S1 Dataset IPV among young women in Maputo. File with data on IPV.
(PDF)
