# Supplementary material for: Risk factors associated with high prevalence of intimate partner violence amongst school-going young women (aged 15–24years) in Maputo, Mozambique
Source: PLoS One. 2020 Dec 9;15(12):e0243304. doi: 10.1371/journal.pone.0243304 (PMC7725391; doi:10.1371/journal.pone.0243304)
Supplement: S1 Appendix — File with English questionnaire. (PDF) [file pone.0243304.s001.pdf]

Self-administered questionnaire

Survey to assess prevalence and factors associated with IPV among young women attending in secondary schools in KaMpfumo municipality district, Maputo- Mozambique.

The questionnaire used in this study to estimate the IPV and associated factors was adapted from the WHO Multi-country Survey of Women's Health and Domestic Violence against Women (Garcia-Moreno, 2005). The WHO Multi-country tools are recommended since they cover issues of IPV, and their validity and reliability have been confirmed. The questionnaire's validity in the Portuguese language was confirmed in the study done in Brazil in two different social contexts (urban and rural). The results indicated the adequacy of the instrument in estimating the occurrence of IPV and the associated factors. The study reported a Cronbach alpha coefficient of 0.88. Thus, the instrument has been shown to be reliable, consistent and adequate to be used in other similar studies accessing IPV, in different contexts such as this study (Schraiber at al., 2010). The IPV was measured both across the lifetime and in the 12 months prior to conduct the survey. The questionnaire was translated from English to Portuguese and back translated into English by a second translator to ensure consistency. The selection of the questions was designed to address the sociocultural context of the young women attending secondary schools in Maputo, based on the information from the focus groups.

The questionnaire was piloted in a school with a similar setting, but not included in the study, amongst 42 young women (10%), to ensure clarity of the questions and consistency in the methods of questioning and the data collection procedure. After the pilot, some issues relating to demographic information were re-formulated for the school-going population in an urban setting in Maputo. The independent variables were divided into two sections. Section one comprised socio-demographic characteristics measured as categorical variables and Section two investigated the socio-cultural risk factors for IPV considering agreement or disagreement with statements of male superiority and the statements of acceptance of IPV. These were measured as a 4-point Likert scale from strongly agree, agree, disagree and strongly disagree.

Part A. Introduction- to be completed by the researcher team member

1	Study identification number	
2	School name	
3	Research team name	
4	Date of survey	DD/MM/YY<br>____/____/______

Thank you for agreeing to be part of the study on factors influencing IPV among young women (15-24 years) in KaMPfumo municipality district. As part of the study, we would like to request you to complete this questionnaire. Please not that there are no wrong or right answers to the questions in this form.

Part B. To be completed by participants

Section 1- Demographics

	QUESTION	OPTIONS OF ANSWER	EXPLANATION
1.1	How old are you?	1. Indicate the age you complete this year 2019------	
1.2	Where do you live?	1. Area inside Kamfumo____<br>2. Area outside Kampfumo____<br>3. Name the area---------------------------	
1.3	What is your completed educational level?	1. Grade 7____<br>2. Grade 8____<br>3. Grade 9____<br>4. Grade 10____<br>5. Grade 11_____	
14	Are you doing any financial activity?	1. Employed___<br>2. Not working____<br>3. Self employed____	
1.5	Do you consider yourself committed with any religion?	1. Yes____<br>2. No____	It refers to degree to which you adhere to religious values, beliefs, and practices and uses them in daily living.
1.7	Where did you grow up? Area	1. Area Inside city____<br>2. Area Outside city____	
1.8	What is your status of relationship?	1. Married___<br>2. Currently in a relationship____	

		3. Currently no relationship but ever had<br>4. Occasional partner____<br>5. Never been in relationship_______	
1.9	With whom do you live<br>(mark with x all that applies)	1. Both parents___<br>2. Father only___<br>3. Mother only___<br>4. Grandparent(s)<br>5. Extended family (aunt, uncle, etc.) ___<br>6. If other, please explain_______	
1.10	With whom did you grow up (Mark with x all that applies)	1. Both parents___<br>2. Father only___<br>3. Mother only___<br>4. Grandparent(s)<br>5. Extended family (aunt, uncle, etc.) ___<br>6. If other, please explain_______	
**Please respond about the head of your household. Consider the head of household the person who is responsible in providing subsistence for the family**			
1.11	What is the highest standard or grade of the head of household?	1. Primary school___<br>2. Secondary school (basic)___<br>3. Secondary school (media)___<br>4. Degree___<br>5. Postgraduate_<br>6. Alphabetic___<br>7. Analphabetic___	
1.12	Is your head of household employed	1. Employed___<br>2. Not working____<br>____	
**Partner background- please refers to your current partner or most recent partner (partner is a male partner that you have/ever had romantic relationship including sexual activities. This can be a boyfriend, spouse, or any occasional partner**			
1.13	Does your current partner or most recent partner an alcohol user?	1. Yes____<br>2. 2. No____<br><br>3. Don't Know____	
1.14	What your current or most recent partner do for financial income?	1. Employed___<br>2. Not working____<br>4. Don't Know___	
1.15	How many years is your current or most recent partner	1. 10 years or more older____	

	older than you?	2. Less than 10 years older____<br>3. Younger than me /same age___<br>4. Don't know___	
	**Section 2. sociocultural factors**		
	**This section constitutes the agreement with statement of male superiority**		
2.1	Do you believe that a man has a superior position within a society than women?	1.Strongly agree___<br>2. Agree____<br>3. Disagree____<br>4. Strongly disagree____	
2.2	Do you think is justified man having more than one partner if he wants?	1.Strongly agree___<br>2. Agree____<br>3. Disagree____<br>4. Strongly disagree___	
2.3	More encouragement in a family should be given to sons than daughters to go to college.	1.Strongly agree___<br>2. Agree____<br>3. Disagree____<br>4. Strongly disagree____	
2.4	In general, the father should have greater authority than the mother in making family decisions.	1.Strongly agree___<br>2. Agree____<br>3. Disagree____<br>4. Strongly disagree____	
2.5	It is more important for boys than girls to do well in school.	1.Strongly agree___<br>2. Agree____<br>3. Disagree____<br>4. Strongly disagree____	
2.6	Boys are better leaders than girls	1.Strongly agree___<br>2. Agree____<br>3. Disagree____<br>4. Strongly disagree____	
2.7	Girls should be more concerned with becoming good wives and mothers rather than desiring a professional or business career.	1.Strongly agree___<br>2. Agree____<br>3. Disagree____<br>4. Strongly disagree____	
2.8	A man can't control their sexual desire; in that way the partner must attend him when he wants sex	1.Strongly agree___<br>2. Agree____<br>3. Disagree____<br>4. Strongly disagree____	
	**Statement of acceptance of violence**		
2.9	Violence between intimate partners can improve the relationship	1.Strongly agree___<br>2. Agree____<br>3. Disagree____	

		4. Strongly disagree____	
2.10	Women sometimes deserve to be hit by their romantic partners.	1.Strongly agree___<br>2. Agree____<br>3. Disagree____<br>4. Strongly disagree____	
2.11	A woman who makes her partner jealous on purpose deserves to be hit	1.Strongly agree___<br>2. Agree____<br>3. Disagree____<br>4. Strongly disagree____	
2.12	There are times when violence by men to women is okay	1.Strongly agree___<br>2. Agree____<br>3. Disagree____<br>4. Strongly disagree____	
2.13	Sometimes violence is the only way in men to express feelings	1.Strongly agree___<br>2. Agree____<br>3. Disagree____<br>4. Strongly disagree____	
2.14	Some women must accept violence from their partners to solve their problems	1.Strongly agree___<br>2. Agree____<br>3. Disagree____<br>4. Strongly disagree____	
2.15	Violence between intimate partners is a personal matter and people should not interfere.	1.Strongly agree___<br>2. Agree____<br>3. Disagree____<br>4. Strongly disagree____	
2.16	A man has all right to hit his partner, just to correct her	1.Strongly agree___<br>2. Agree____<br>3. Disagree____<br>4. Strongly disagree____	
**Section 3- IPV experiences**			
**When two people have together a romantic relationship, they usually share both good and bad moments. I would now like to ask you to respond some questions about your current and past relationships and how your partner treats /treated you (since you were 15 years old). I would again like to assure you that your answers will be kept confidential and anonymous, and that to assure feasible results, I would like to ask you to try to answer all questions.**			
	**Physical Violence – Has he or any other partner ever did some of the following**		**Has this happened in the past 12 months with your current partner or partner you had in last 12 months?**

3.1	Slapped you or thrown something at you that could hurt you?	1.____yes<br>2. ____no	1.____yes<br>2. ____no
3.2	Pushed you or shoved you or pulled your hair?	1.____yes<br>2. ____no	1.____yes<br>2. ____no
3.3	Hit you with his fit or with something else that could hurt you?	1.____yes<br>2. ____no	1.____yes<br>2. ____no
3.4	Kicked you, dragged you or beaten you up?	1.____yes<br>2. ____no	1.____yes<br>2. ____no
3.5	Choked or burnt you on purpose?	1.____yes<br>2. ____no	1.____yes<br>2. ____no
3.6	Threatened you with or used a gun, knife or another weapon against you?	1.____yes<br>2. ____no	1.____yes<br>2. ____no
	**Sexual violence- Has your current or any other partner ever did some of the following acts?**		**Has this happened in the past 12 months with your current partner or partner you had in last 12 months?**
3.7	Has a current or previous partner physically forced you to have sexual intercourse when you did not want to?	1.____yes<br>2. ____no	1.____yes<br>2. ____no
3.8	Did your current or previous partner ever physically force you to have sexual acts when you did not want to, for example, by twisting your arm or holding you down?	1.____yes<br>2. ____no	1.____yes<br>2. ____no
3.9	Did your current or previous partner ever force you to have	1.____yes<br>2. ____no	1.____yes<br>2. ____no

	sexual intercourse with him even when you did not want to?		
3.10	Have you had sexual intercourse when you did not want to because you were afraid your partner might hurt or abandon you?	1.____yes<br>2. ____no	1.____yes<br>2. ____no
3.11	Did you ever have sexual intercourse when you did not want to because you were afraid of what your partner might do if you refused?	1.____yes<br>2. ____no	1.____yes<br>2. ____no
3.12	Has your partner used threats or intimidation (but not physical force) to get you to have sexual intercourse when you did not want to?	1.____yes<br>2. ____no	1.____yes<br>2. ____no
3.13	Has a current or previous partner made you do sexual things that you found degrading or humiliating?	1.____yes<br>2. ____no	1.____yes<br>2. ____no
3.14	Did your current or previous partner ever forced you to perform sexual acts (other than vaginal intercourse) when you did not want to?	1.____yes<br>2. ____no	1.____yes<br>2. ____no
	**Psychological Abuse- Has your current or any other partner ever did some of the following?**		**Has this happened in the past 12 months with you current partner or partner you had in last 12 months?**
3.15	Called you insulting names?	1.____yes<br>2. ____no	1.____yes<br>2. ____no
3.16	Swore at you?	1.____yes<br>2. ____no	1.____yes<br>2. ____no
3.17	Yelled and screamed at you?	1.____yes<br>2. ____no	1.____yes<br>2. ____no

3.18	Treated you like an inferior?	1.____yes<br>2. ____no	1.____yes<br>2. ____no
3.19	Told your feelings were irrational or crazy?	1.____yes<br>2. ____no	1.____yes<br>2. ____no
3.20	Blamed you for his problems?	1.____yes<br>2. ____no	1.____yes<br>2. ____no
3.21	Tried to make you feel crazy?	1.____yes<br>2. ____no	1.____yes<br>2. ____no
3.22	Monitored your time and made your account for your whereabouts?	1.____yes<br>2. ____no	1.____yes<br>2. ____no
3.23	Used your money or made important financial decisions without talking to you about it?	1.____yes<br>2. ____no	1.____yes<br>2. ____no
3.24	Jealous or suspicious of your friends that restricted them?	1.____yes<br>2. ____no	1.____yes<br>2. ____no
3.25	Restricted your use of the telephone?	1.____yes<br>2. ____no	1.____yes<br>2. ____no

Would you like to make some questions?____

____

____

Would you like to add some information/comments?

____

End of the questionnaire.

Explanation:

Religious Commitment

Is defined as the degree to which you adhere to your religious values, beliefs, and practices and use them in daily living.

Is operationalized in the contest of this study as the report in yes/no responses to religious commitment.

Intimate partner (current or previous)

Is the male partner with whom you have or ever had a romantic relationship, since the age of 15 years. Can be your spouse/husband, boyfriend, dating partner, or ongoing sexual partner.

Head of Household

Is the person who is responsible for providing subsistence
