# Supplementary material for: Risk factors associated with high prevalence of intimate partner violence amongst school-going young women (aged 15–24years) in Maputo, Mozambique
Source: PLoS One. 2020 Dec 9;15(12):e0243304. doi: 10.1371/journal.pone.0243304 (PMC7725391; doi:10.1371/journal.pone.0243304)
Supplement: S2 Appendix — File with Portuguese questionnaire. (PDF) [file pone.0243304.s002.pdf]

APENDIX D

Questionário Auto Administrado (português)

Inquérito para investigar a prevalência e os factores associados a VPI em mulheres jovens de 15-24 anos de idade no distrito municipal KaMpfumo, na Cidade Maputo

Parte A. Introdução- a ser preenchida pelos membros da equipa de investigação

1	Número de identificação do estudo	
2	Nome da escola	
3	Nome dos investigadores	
4	Data do inquérito	DD/MM/AA<br>____/____/______

Obrigada por aceitar fazer parte do estudo, sobre factores socio-culturais que influenciam a violência pelo parceiro intimo em mulheres jovens (15-24 anos de idade) no Distrito Municipal KaMpfumo na Cidade de Maputo. Como parte do estudo, gostariamos de pedir para preencher este questionário. Por favor note que não existem respostas erradas ou certas nas perguntas colocadas neste formulário.

Part B. A ser preenchida pelos participantes

Secção 1- Dados demográficoss - Nesta secção escolha apenas uma alternativa

	PERGUNTA	OPÇÕES DE RESPOSTAS	explicaçao
1.1	Em que faixa etária voce se enquadra?	1. 15-17 ____<br>2. 18-20___<br>3. 21-24__<br>4. Marrque a idade que voce completa em 2019------	Marque neste espaço a idade em anos que completa/ou em 2019
1.**2**	Onde voce vive?	1. Area dentro da cidade<br>2. Area suburbana/periferia fora da cidade<br>3. Por favor indique o nome do bairro-------------------	

1.3	Qual é o teu nível de educação completo?<br>(Indica a classe concluida em 2018)	1. 7a classe____<br>2. 8a classe____<br>3. 9a classe____<br>4. 10a classe____<br>5. 11a classe____	
1.4	Voce realiza alguma actividade remuneravel?	1. Empregada___<br>2. Nao trabalho____<br>3. Auto emprego/negocio____	
1.5	Voce se considera uma pessoa comprometida com a religião? Ou cumpridora dos principios e regras da sua religião?	1. Sim____<br>2. Nao____	Considere comprometida com religiao se voce se baseia nos principios religiosos para a sua vivencia no dia a dia
1.7	Em que area voce cresceu? Ou foi criada?	1. Area urbana dentro da cidade____<br>2. Area rural fora da cidade____<br>3. Por favor coloque o nome da região ou cidade/bairro onde voce foi criada___________________	
1.8	Qual é o teu estado civil ou de relacionamento romantico?	1. Casado/vive maritalmente----_<br>2. Actualmente tenho Namorado ____<br>3. Actualmente sem relacionamento romantico mas já tive_____	

		4. Actualmente em Relacionamento ocasional____<br>5. Nunca estive em relacionamento romantico/nunca namorei______<br>________________________________<br>___________________________________	
1.9	Com quem voce vive?<br>(marque com x todas alternativas aplicaveis)	1. Com pai e mae___<br>2. Somente com pai___<br>3. Somente com mãe___<br>4. Avó (s)--------<br>5. Outros familiares (tia, tio, etc.) ___	
1.10	Com quem voce cresceu ou foi criado?<br>(marque com x todas alternativas aplicaveis)	1. Somente com pai___<br>2. Somente com mãe___<br>3. Com pai e mae_____<br>4. Avó (s)<br>5. Outros familiares (tia, tio, etc.) ___	
**Por favor, responda sobre o chefe de familia. Considere chefe de familia a pessoa responsavel por prover a subsistencia da familia**			
1.11	Qual é o maior grau educational do chefe de familia	1. Escola primaria___<br>2. Escola secundaria basico___<br>3. Escola secundaria medio___<br>4. Licenciatura___<br>5. Mestrado/Doutorado___<br>6. Alfabetizado___<br>7. Nao estudou_____<br>8. Nao sei-----	

1.12	O que faz o chefe de familia como actividade rentavel?	1. Empregado___<br>2. Não trabalha____	
**Por favor responda sobre o seu parceiro intimo. Parceiro intimo é o homem com que voce tem ou teve uma relacaco remantica que envolve relacoes sexuais, pode ser marido, namorado ou um parceiro ocasional. Considerar parceiro actual se actualmente tiver ou considerar parceiro passado se actualmente não tem parceiro mas teve desde a idade de 15 anos**			
1.13	O teu parceiro intimo actual ou passado é consumidor de alchool?	1.Sim____<br>2. Não____<br>3.Nao sei----	
1.14	O que faz o teu parceiro intimo actual ou passado como actividade rentável?	1. Empregado___<br>2. Não trabalha____<br>5. Não sei____	
1.15	Quantos anos mais velho que tu é o teu parceiro intimo actual ou passado?	1 Menos de 10 anos mais velho____<br>2 Mais de 10 anos mais velho___<br>3 Mais jovem que eu/mesma idade________<br>4 Não sei/não me lembro_____	
	**Secçao 2- factores socioculturais de superioridade masculina e justificação de violencia- Diga ate que ponto voce concorda ou discorda com as seguintes afirmacoes**		
2.1	Você acredita que um homem tem uma posição superior dentro de uma sociedade do que as mulheres?	1.Concordo fortemente___<br>2. Concordo____<br>3. Discordo____<br>4. Discordo fortemente_______	
2.2	Você acha que existem razoes ou justificaçao aceitavel para o homem ter mais de uma parceira se ele quiser?	1.Concordo fortemente___<br>2. Concordo____<br>3. Discordo____<br>4. Discordo fortemente	
2.3	Mais incentivo para ir a faculdade em uma família,	1.Concordo fortemente___<br>2. Concordo____	

	deve ser dado aos filhos do que às filhas	3. Discordo____<br>4. Discordo fortemente	
2.4	Em geral, o pai deve ter maior autoridade do que a mãe na tomada de decisões familiares.	1.Concordo fortemente___<br>2. Concordo____<br>3. Discordo____<br>4. Discordo fortemente	
2.5	É mais importante que os meninos se saiam bem na escola do que as meninas.	1.Concordo fortemente___<br>2. Concordo____<br>3. Discordo____<br>4. Discordo fortemente	
2.6	Os meninos são melhores líderes que as meninas	1.Concordo fortemente___<br>2. Concordo____<br>3. Discordo____<br>4. Discordo fortemente	
2.7	As meninas devem se preocupar mais em se tornar boas esposas e mães do que em desejar uma carreira profissional ou de negócios.	1.Concordo fortemente___<br>2. Concordo____<br>3. Discordo____<br>4. Discordo fortemente	
2.8	Um homem não pode controlar seu desejo sexual; dessa forma a parceira deve atendê-lo sempre que ele quiser ter relacoes sexuais	1.Concordo fortemente___<br>2. Concordo____<br>3. Discordo____<br>4. Discordo fortemente	
	**Razoes da violencia do homen contra a parceira**		
2.9	A violência entre parceiros íntimos por vezes pode melhorar a relaçao entre o casal	1.Concordo fortemente___<br>2. Concordo____<br>3. Discordo____<br>4. Discordo fortemente	
2.1	As mulheres às vezes	1.Concordo fortemente___	

0	merecem ser batidas por seus parceiros intimos	2. Concordo____<br>3. Discordo____<br>4. Discordo fortemente	
2.1<br>1	Uma mulher que provoca ciumes ao seu parceiro de propósito merece ser batida	1.Concordo fortemente___<br>2. Concordo____<br>3. Discordo____<br>4. Discordo fortemente	
2.1<br>2	Há momentos em que a violência de homens para mulheres é boa	1.Concordo fortemente___<br>2. Concordo____<br>3. Discordo____<br>4. Discordo fortemente	
2.1<br>3	Às vezes a violência é a única maneira que os homens tem de expressar sentimentos de amor pela parceira	1.Concordo fortemente___<br>2. Concordo____<br>3. Discordo____<br>4. Discordo fortemente	
2.1<br>4	As vezes as mulheres devem aceitar a violência de seus parceiros para resolver seus problemas	1.Concordo fortemente___<br>2. Concordo____<br>3. Discordo____<br>4. Discordo fortemente	
2.1<br>5	A violência entre parceiros íntimos é uma questão pessoal e as pessoas não devem interferir	1.Concordo fortemente___<br>2. Concordo____<br>3. Discordo____<br>4. Discordo fortemente	
2.1<br>6	Um homem tem todo o direito de bater em sua parceira, para corrigi-la	1.Concordo fortemente___<br>2. Concordo____<br>3. Discordo____<br>4. Discordo fortemente	
	**Section 3- Experiencias de VPI**		

Quando duas pessoas têm um relacionamento romântico, elas geralmente compartilham bons e maus momentos. Gostaria agora de pedir-lhe para responder a algumas perguntas sobre os seus relacionamentos atuais e passados e sobre como o seu parceiro o tratou / trata (desde a sua idade de 15 anos). Gostaria de garantir que suas respostas serão mantidas confidenciais e anônimas e que, para garantir resultados viáveis, gostaria de pedir que você tentasse responder a todas as perguntas			
	**Violencis física – Alguma vez na vida o seu parceiro actual ou passado fez uma das seguintes coisas?**		**Isto já aconteceu nos passados 12 meses com o teu actual parceiro ou o parceiro que teve nos ultimos 12 meses?**
3.1	Deu um tapa ou jogou algo em você que poderia machucar você?	1.____Sim<br>2. ____Não	1.____Sim<br>2. ____Não
3.2	Empurrou você ou puxou seu cabelo?	1.____Sim<br>2. ____Não	1.____Sim<br>2. ____Não
3.3	Bateu você com o seu pé ou com algo mais que poderia machucar você?	1.____Sim<br>2. ____Não	1.____Sim<br>2. ____Não
3.4	Chutou você, arrastou você ou te espancou?	1.____Sim<br>2. ____Não	1.____Sim<br>2. ____Não
3.5	Chocou ou queimou você de propósito?	1.____Sim<br>2. ____Não	1.____Sim<br>2. ____Não
3.6	Ameaçou você com ou usou uma arma, faca ou outra arma contra você?	1.____Sim<br>2. ____Não	1.____Sim<br>2. ____Não

	**Violence sexual- Alguma vez o seu parceiro actual ou passado fez uma das seguintes coisas?**		**Isto já aconteceu nos passados 12 meses com o teu actual parceiro ou o parceiro que teve nos ultimos 12 meses?**
3.7	Alguma vez o seu parceiro atual ou anterior fisicamente forçou você a ter relação sexual quando você não queria?	1.____Sim<br>2. ____Não	1.____Sim<br>2. ____Não
3.8	Alguma vez o seu parceiro atual ou anterior a forçou a praticar atos sexuais quando você não queria, por exemplo, torcendo o braço ou segurando-o para baixo ou te imobilizando?	1.____Sim<br>2. ____Não	1.____Sim<br>2. ____Não
3.9	Já alguma vez o seu parceiro atual ou anterior a forçou a ter relações sexuais com ele mesmo quando você não queria?	1.____Sim<br>2. ____Não	1.____Sim<br>2. ____Não
3.10	Você já teve relações sexuais quando não queria porque estava com medo de que seu	1.____Sim<br>2. ____Não	1.____Sim<br>2. ____Não

	parceiro a ferisse ou abandonasse?		
3.11	Você já teve relações sexuais quando não queria porque estava com medo do que seu parceiro faria se recusasse?	1.____Sim<br>2. ____Não	1.____Sim<br>2. ____Não
3.12	O seu parceiro já usou ameaças ou intimidação (mas não força física) para conseguir que você tenha relações sexuais quando voce não queria?	1.____Sim<br>2. ____Não	1.____Sim<br>2. ____Não
3.13	Alguma vez o seu parceiro actual ou anterior fez você fazer coisas sexuais que você achou humilhantes ou contra seus principios morais?	1.____Sim<br>2. ____Não	1.____Sim<br>2. ____Não
3.14	Alguma vez o seu atual ou anterior parceiro já a forçou a realizar outros atos sexuais (além do sexo vaginal) quando você não queria?	1.____Sim<br>2. ____Não	1.____Sim<br>2. ____Não
	**Abuso psicológico- Alguma vez o seu parceiro actual ou passado fez uma das seguintes coisas?**		**Isto já aconteceu nos passados 12 meses com o teu actual parceiro ou o parceiro que teve nos ultimos 12 meses?**

3.15	Te chamou de nomes ofensivos?	1.____Sim<br>2. ____Não	1.____Sim<br>2. ____Não
3.16	Humilhou te?	1.____Sim<br>2. ____Não	1.____Sim<br>2. ____Não
3.17	Gritou com você?	1.____Sim<br>2. ____Não	1.____Sim<br>2. ____Não
3.18	Tratou te como inferior a ele?	1.____Sim<br>2. ____Não	1.____Sim<br>2. ____Não
3.19	Disse que seus sentimentos eram irracionais ou de loucos despresando-os?	1.____Sim<br>2. ____Não	1.____Sim<br>2. ____Não
3.20	Culpou você pelos problemas dele?	1.____Sim<br>2. ____Não	1.____Sim<br>2. ____Não
3.21	Tentou fazer voce se sentir maluca ou sentir se sem valor?	1.____Sim<br>2. ____Não	1.____Sim<br>2. ____Não
3.22	Monitorou seu tempo e fez te prestar contas do seu paradeiro?	1.____Sim<br>2. ____Não	1.____Sim<br>2. ____Não
3.23	Usou seu dinheiro ou tomou importantes decisões financeiras sem falar com você sobre isso?	1.____Sim<br>2. ____Não	1.____Sim<br>2. ____Não
3.24	Ciumento ou faz suspeitas e desconfianças ou acusaçoes de voce trai-lo com os teus amigos?	1.____Sim<br>2. ____Não	1.____Sim<br>2. ____Não
3.25	Restringiu/controla/proibe o uso do seu telefone?	1.____Sim<br>2. ____Não	1.____Sim<br>2. ____Não

Fim do inquérito.

Muito obrigada pela sua participação.

Gostaria de colocar algumas questões?

Gostaria de adicionar algumas informações / comentários?

____

____

____

Nota: Considere parceiro actual se actualmente estiver em uma relação.

Considere **parceiro mais recente** o último parceiro com quem esteve relacionado.

Considere **últimos doze meses** os doze meses passados ate a altura do preenchimento do inquerito.

Considere **parceiro intimo** qualquer parceiro homen com quem tem ou teve relação romantica que envolve relacoes sexuais, pode ser namorado, marido, amante ou outro.

Considere se pessoa **comprometido com a religiao** se voce acredita na religião e considera o uso dos principios da sua religião nas suas atitudes e comportamentos do dia a dia.
