# Supplementary material for: Risk factors associated with high prevalence of intimate partner violence amongst school-going young women (aged 15–24years) in Maputo, Mozambique
Source: PLoS One. 2020 Dec 9;15(12):e0243304. doi: 10.1371/journal.pone.0243304 (PMC7725391; doi:10.1371/journal.pone.0243304)
Supplement: S1 Dataset — File with data on IPV. (PDF) [file pone.0243304.s003.pdf]

ID	Age	Idade	Bairro	EducLevel	Financialac	Religioncor	Placeofbor	Areagrownu
1,00	2	20	99	5	2	1	1	2
2,00	2	20	2	4	2	1	1	2
3,00	2	20	2	5	2	2	1	2
4,00	2	20	2	5	2	1	1	2
5,00	2	20	2	5	2	1	1	2
6,00	2	20	1	5	2	1	1	1
7,00	2	20	2	5	2	1	1	2
8,00	2	19	2	5	2	1	1	1
9,00	2	19	2	5	2	1	1	1
10,00	2	19	2	5	2	2	1	2
11,00	2	19	2	5	2	2	1	1
12,00	2	19	99	4	2	2	1	2
13,00	2	19	2	5	2	1	1	1
14,00	2	20	2	5	2	2	1	2
15,00	2	19	2	5	2	1	1	2
16,00	2	19	99	5	2	1	1	1
17,00	2	19	99	5	2	2	1	99
18,00	2	19	2	5	2	1	1	2
19,00	2	19	2	5	2	2	1	2
21,00	2	20	1	5	2	1	1	1
22,00	2	20	1	5	3	2	1	1
23,00	2	20	2	5	2	2	1	2
24,00	2	20	2	5	2	2	1	2
25,00	2	20	2	5	2	1	1	2
27,00	2	20	2	5	2	1	1	2
28,00	2	20	2	5	2	1	1	2
30,00	2	20	2	5	3	1	1	2
31,00	2	20	2	5	2	2	1	2
32,00	3	24	99	4	2	1	1	2
33,00	3	22	2	4	3	2	1	2
34,00	3	24	99	4	2	1	1	2
35,00	3	24	1	5	2	1	1	1
36,00	3	24	2	5	3	2	1	2
37,00	3	24	2	5	1	2	1	2
38,00	3	24	1	5	3	1	1	1
39,00	3	24	2	4	2	1	1	2
40,00	3	22	2	5	1	1	1	2
41,00	3	22	2	4	3	1	1	2
42,00	3	22	2	4	99	99	1	2
43,00	3	23	2	5	1	1	1	2
45,00	3	21	1	4	3	2	1	1
46,00	3	21	2	5	2	1	1	2
47,00	3	21	99	4	3	2	1	2
48,00	3	21	2	4	2	1	1	2
49,00	3	21	2	5	2	1	1	2
50,00	3	21	2	5	2	1	1	2
51,00	2	18	2	4	2	1	1	2
52,00	2	18	2	5	2	1	1	2
53,00	2	18	1	5	2	1	1	1

54,00	2	18	2	4	2	2	1	2
55,00	1	17	2	4	2	99	1	2
56,00	1	17	1	5	2	1	1	1
59,00	1	17	1	4	1	1	1	1
60,00	1	17	99	4	2	2	1	1
62,00	2	20	2	5	2	2	1	2
63,00	2	20	2	4	2	1	1	2
64,00	2	19	1	4	2	1	1	1
65,00	2	19	99	4	2	2	1	2
66,00	2	19	2	5	2	1	1	2
67,00	2	19	1	5	2	2	1	1
68,00	2	18	2	4	3	1	1	2
69,00	2	18	99	4	99	2	1	99
70,00	2	18	2	4	3	1	1	2
71,00	2	18	2	5	2	2	1	2
72,00	2	18	2	5	3	1	1	2
73,00	2	18	99	5	2	2	1	2
74,00	2	18	1	4	2	1	1	1
75,00	2	18	1	4	99	1	1	1
76,00	2	18	99	5	2	2	1	2
78,00	2	18	2	5	2	1	1	2
79,00	2	18	99	5	99	1	1	1
80,00	2	18	2	5	2	2	1	2
81,00	2	18	99	5	2	1	1	2
82,00	2	18	2	5	1	1	2	2
83,00	2	18	2	5	99	99	1	2
84,00	2	18	2	5	2	2	1	2
85,00	2	18	2	5	2	1	1	2
86,00	2	18	2	5	2	1	1	2
87,00	2	18	2	5	2	2	1	1
88,00	1	17	1	4	2	1	1	1
89,00	1	17	1	4	2	1	1	1
90,00	1	17	1	4	2	1	1	1
91,00	1	17	2	5	2	1	1	2
92,00	1	17	2	5	2	2	1	2
93,00	1	17	1	4	2	1	1	1
94,00	1	17	99	5	2	2	1	1
95,00	1	17	1	4	99	1	1	1
96,00	1	17	2	5	2	1	1	2
97,00	1	17	2	5	2	2	1	2
98,00	1	17	1	5	2	99	1	1
99,00	2	18	2	4	2	99	1	2
100,00	2	18	1	5	2	1	1	1
101,00	2	18	2	5	2	1	1	1
102,00	2	18	2	4	2	2	1	2
103,00	2	18	2	4	2	99	2	2
104,00	2	18	1	4	2	1	1	1
105,00	2	18	1	4	2	2	1	1
106,00	2	18	2	5	3	1	1	1
107,00	2	18	1	5	2	1	1	1

108,00	2	18	2	5	2	1	1	2
109,00	2	18	1	5	2	1	1	1
110,00	1	17	2	4	2	2	1	2
111,00	1	17	2	4	2	1	1	2
112,00	1	17	2	4	1	1	2	2
113,00	1	17	2	4	2	99	1	2
114,00	2	19	1	4	3	1	1	1
115,00	2	19	1	4	2	2	1	1
116,00	2	19	2	4	2	1	1	2
117,00	2	19	2	4	2	1	1	2
118,00	2	19	2	5	2	1	1	2
119,00	2	19	2	4	2	2	1	1
120,00	2	19	1	4	2	1	1	1
121,00	2	19	1	5	2	1	1	1
122,00	2	19	2	4	2	1	1	2
123,00	2	19	2	4	2	1	1	2
124,00	2	19	2	4	2	1	1	2
125,00	2	19	2	4	2	1	1	2
126,00	2	20	2	5	2	2	1	2
127,00	2	20	1	5	2	1	1	1
128,00	2	20	1	5	2	2	1	2
129,00	2	20	2	5	2	2	1	2
130,00	2	20	2	4	2	1	1	2
131,00	2	20	2	4	2	1	1	2
132,00	2	20	2	4	2	2	1	2
133,00	2	20	2	5	2	1	1	2
134,00	2	20	1	5	2	1	1	1
135,00	2	20	1	5	2	2	1	1
136,00	2	20	2	4	2	1	1	2
138,00	1	16	2	5	2	1	1	2
139,00	1	16	2	4	2	1	1	2
140,00	1	16	2	4	2	2	1	2
141,00	1	16	2	4	2	1	1	2
142,00	1	16	1	4	2	1	1	1
143,00	1	16	1	4	2	2	1	1
144,00	1	16	2	4	2	1	1	2
145,00	1	16	2	4	1	2	1	1
146,00	1	16	2	4	2	1	1	2
147,00	1	17	2	5	2	2	1	2
148,00	1	16	1	4	2	2	1	1
149,00	1	16	1	5	2	1	1	1
150,00	1	16	2	4	2	1	1	2
151,00	1	16	1	4	2	1	1	1
152,00	1	16	2	4	2	1	1	2
154,00	1	16	2	4	2	2	1	2
155,00	1	16	2	4	2	2	1	2
156,00	1	17	1	4	2	1	1	1
157,00	1	17	2	4	2	1	1	2
158,00	1	17	2	5	2	1	1	2
159,00	1	17	2	4	2	1	1	2

160,00	1	17	2	4	2	1	1	2
162,00	1	17	1	5	2	1	1	1
163,00	1	17	2	5	2	1	1	2
164,00	1	17	1	5	2	1	1	1
165,00	1	17	1	5	3	1	1	1
166,00	1	17	2	5	3	1	1	2
167,00	1	17	2	5	2	1	1	2
168,00	1	17	2	4	2	2	1	2
169,00	1	17	2	4	2	99	1	2
170,00	1	17	2	4	2	1	1	1
171,00	1	17	2	4	2	1	1	2
172,00	1	17	2	4	2	1	1	2
173,00	1	17	2	4	2	2	1	2
174,00	1	17	1	4	2	1	1	1
175,00	1	17	2	4	3	1	1	2
176,00	1	17	2	4	2	1	1	1
177,00	1	17	1	4	2	1	1	1
178,00	1	17	1	4	3	1	1	1
179,00	1	17	2	4	2	1	1	2
180,00	1	17	2	5	2	1	1	2
181,00	1	17	2	5	2	1	1	2
182,00	1	17	1	4	2	1	1	1
183,00	1	17	2	5	2	1	1	2
184,00	1	17	2	5	2	2	1	2
185,00	1	17	2	5	2	2	1	2
186,00	1	17	2	5	2	1	1	2
187,00	1	17	2	5	2	2	1	2
188,00	1	17	2	4	2	1	1	2
189,00	1	17	1	4	2	1	1	1
190,00	1	17	1	4	2	1	1	1
191,00	1	17	1	4	2	1	1	1
192,00	1	17	2	4	2	1	1	2
193,00	1	17	2	4	2	1	1	2
194,00	1	17	1	5	2	1	1	1
195,00	1	16	2	4	2	2	1	1
196,00	1	17	1	4	2	1	1	1
197,00	1	17	1	1	2	2	1	1
198,00	1	17	2	4	2	1	1	2
199,00	1	17	2	4	2	1	1	2
200,00	2	18	2	5	3	1	1	2
201,00	2	18	2	4	2	1	1	2
202,00	2	18	2	5	3	2	1	2
203,00	2	18	2	5	2	2	1	2
204,00	2	18	2	4	2	2	1	2
205,00	2	18	2	4	2	2	1	2
206,00	2	18	1	4	2	1	1	1
207,00	2	18	1	4	2	1	1	1
208,00	2	18	2	4	2	2	1	2
209,00	2	18	2	5	2	1	2	2
210,00	2	18	2	5	2	1	1	2

211,00	2	18	2	5	2	2	2	1
212,00	2	18	1	4	2	1	1	1
213,00	2	18	2	5	2	2	1	2
214,00	2	18	1	4	2	1	1	1
215,00	2	16	2	4	2	1	1	2
216,00	2	18	1	4	2	99	1	1
217,00	2	18	2	4	2	2	1	2
218,00	2	18	1	5	2	1	1	1
219,00	2	18	2	4	2	1	1	2
220,00	2	18	2	4	2	1	1	2
221,00	2	18	2	4	2	1	1	2
222,00	2	18	2	4	2	1	1	2
223,00	2	18	2	5	2	1	1	2
224,00	2	18	2	4	2	1	1	2
225,00	2	18	1	4	2	1	1	1
226,00	2	18	2	4	2	1	1	2
227,00	2	18	2	4	2	2	1	2
228,00	2	18	2	4	2	2	1	2
229,00	2	18	1	5	2	2	1	1
230,00	2	18	1	5	2	1	1	1
231,00	2	18	1	5	2	2	1	1
232,00	2	18	1	5	2	2	1	1
233,00	2	18	1	5	2	2	1	1
234,00	2	18	2	4	2	2	1	2
235,00	2	18	1	4	2	2	1	1
236,00	2	18	1	4	2	2	1	2
237,00	2	18	2	4	2	1	1	1
238,00	2	18	1	4	2	1	1	1
239,00	2	18	2	4	2	2	1	2
240,00	2	18	1	4	2	1	1	1
241,00	2	18	1	4	2	1	1	1
242,00	2	18	2	5	2	1	1	2
243,00	2	18	1	5	3	2	1	1
244,00	2	18	2	4	2	2	1	2
245,00	2	18	2	5	2	2	1	1
246,00	2	18	1	4	2	1	1	1
247,00	2	20	2	5	2	1	1	2
248,00	2	20	2	5	2	1	1	2
249,00	2	20	2	5	2	2	1	2
250,00	2	20	1	5	2	2	1	1
251,00	2	20	2	5	2	1	1	2
252,00	2	20	1	5	2	1	1	1
253,00	2	20	2	4	2	2	1	2
254,00	2	20	2	5	2	1	1	2
255,00	2	20	2	4	2	1	1	2
256,00	2	20	2	4	2	2	1	2
257,00	2	20	2	4	2	2	1	2
258,00	2	19	1	5	3	1	1	1
259,00	2	19	1	5	3	1	1	1
260,00	2	19	2	5	2	2	1	2

261,00	2	19	1	4	2	2	1	1
262,00	2	19	2	4	2	2	1	2
263,00	2	19	2	4	2	2	1	2
264,00	2	19	1	4	2	2	1	1
265,00	2	19	2	4	2	2	1	2
266,00	2	19	1	5	2	1	1	1
267,00	2	19	2	5	3	1	1	2
268,00	2	19	2	4	2	1	1	2
269,00	2	18	2	4	3	1	1	2
270,00	2	18	2	4	2	2	1	2
271,00	2	18	2	5	3	1	1	2
272,00	2	18	1	5	2	1	1	1
273,00	2	18	2	5	2	1	1	1
274,00	2	18	2	5	2	2	1	2
275,00	2	18	2	4	2	1	1	2
276,00	2	18	1	4	3	1	1	1
277,00	2	18	2	5	2	2	1	1
278,00	2	18	2	5	2	1	1	2
279,00	2	18	2	4	3	1	1	2
280,00	2	18	2	5	2	1	1	2
281,00	2	18	1	5	2	1	1	1
282,00	2	18	1	5	2	1	1	2
283,00	2	18	2	5	2	1	1	2
284,00	2	18	1	5	2	1	1	1
285,00	2	18	2	5	2	2	1	2
286,00	2	18	2	5	2	2	1	2
287,00	2	18	1	4	2	1	1	1
288,00	2	18	2	4	2	1	1	2
289,00	2	18	2	4	2	2	1	2
290,00	2	18	1	4	2	1	1	2
291,00	2	18	2	4	2	2	1	2
292,00	2	18	2	4	2	1	1	2
293,00	2	18	2	4	2	1	1	2
294,00	2	18	2	4	2	1	1	2
295,00	2	18	1	5	2	1	1	2
296,00	2	18	1	5	2	1	1	1
297,00	2	18	2	4	2	1	1	2
298,00	2	18	2	5	2	1	1	2
299,00	2	18	2	5	3	1	1	2
300,00	2	18	1	5	2	1	1	1
301,00	2	18	1	4	2	1	1	1
302,00	2	18	2	4	2	1	1	2
303,00	2	18	2	4	2	1	2	2
304,00	2	18	2	4	2	2	1	2
305,00	2	18	2	4	3	2	1	2
306,00	2	18	2	4	2	2	1	2
307,00	2	18	2	4	2	2	1	2
308,00	2	18	2	4	2	1	1	2
309,00	1	17	2	4	2	1	1	2
310,00	1	17	2	4	2	1	1	2

311,00	1	17	1	4	1	2	2	1
312,00	1	17	2	5	2	2	1	2
313,00	1	17	2	4	2	2	1	2
314,00	1	17	1	4	2	2	1	1
315,00	1	17	2	4	2	2	1	2
316,00	1	17	1	4	2	1	1	1
317,00	1	17	2	4	2	2	1	2
318,00	1	17	2	4	2	2	1	2
319,00	1	17	2	5	2	2	1	2
320,00	1	17	2	4	2	1	1	2
321,00	1	17	1	4	2	1	1	1
322,00	1	17	1	5	2	2	1	2
323,00	1	17	1	5	2	1	1	1
324,00	1	17	1	5	2	1	1	1
325,00	1	17	1	5	2	1	1	1
326,00	1	17	2	5	2	1	1	2
327,00	1	17	2	4	2	1	1	2
328,00	1	17	1	4	2	1	1	1
329,00	1	17	1	4	2	1	1	1
330,00	1	17	1	5	2	2	1	1
331,00	1	17	1	4	2	2	1	2
332,00	1	17	1	4	2	1	1	1
333,00	1	17	1	4	2	1	1	2
334,00	1	17	2	5	2	1	1	2
335,00	1	17	1	4	2	1	1	1
336,00	1	17	2	4	2	2	1	2
337,00	1	17	2	4	2	1	1	2
338,00	1	17	2	4	2	1	1	2
339,00	1	17	1	5	2	1	1	1
340,00	1	17	2	5	2	1	1	2
341,00	1	17	1	4	2	1	1	1
342,00	1	17	1	5	2	2	1	1
343,00	1	17	2	4	2	2	1	2
344,00	1	17	1	5	2	1	1	2
345,00	1	17	2	4	2	2	1	2
346,00	1	17	2	4	2	1	1	2
347,00	1	17	2	5	2	1	1	1
348,00	1	16	1	4	2	2	1	1
349,00	1	16	1	4	2	2	1	2
350,00	1	16	2	4	2	2	1	2
351,00	1	16	2	5	2	2	1	2
352,00	1	16	2	4	2	1	1	2
353,00	1	15	1	4	2	2	1	1
356,00	1	16	1	4	2	1	1	1
357,00	1	16	2	2	2	2	1	2
358,00	1	16	2	4	2	1	1	2
359,00	1	16	1	4	2	1	1	1
360,00	1	16	1	4	2	1	1	1
361,00	1	16	1	4	2	99	1	1
363,00	1	16	1	5	2	2	1	1

364,00	1	16	2	5	2	1	1	2
365,00	1	16	2	5	2	1	1	2
366,00	1	16	2	5	2	1	1	2
367,00	1	16	1	4	2	1	1	1
368,00	1	16	2	4	2	1	1	2
369,00	1	16	2	4	2	1	1	2
371,00	1	16	2	4	2	2	1	2
372,00	1	16	2	4	2	2	1	2
373,00	1	16	2	4	1	2	1	2
374,00	1	16	1	4	2	2	1	1
375,00	1	16	1	4	2	99	1	1
377,00	1	16	1	4	2	1	1	1
378,00	1	16	2	4	2	1	1	2
379,00	1	16	1	4	2	2	1	1
380,00	1	16	2	4	2	1	1	2
381,00	1	16	2	4	2	2	1	2
382,00	1	15	1	4	2	1	1	1
385,00	2	18	2	5	2	2	1	2
386,00	2	18	2	5	2	2	1	2
387,00	2	18	1	5	2	1	1	1
388,00	2	18	2	5	2	1	1	2
389,00	2	18	2	5	2	2	1	2
290,00	2	18	2	5	2	2	1	2
391,00	2	18	1	5	2	1	1	1
392,00	2	18	2	5	2	1	1	2
393,00	2	18	2	5	2	2	1	2
394,00	2	18	2	5	2	1	1	2
395,00	2	18	1	5	2	2	1	1
396,00	2	18	1	5	2	1	1	1
397,00	2	18	2	4	2	1	1	2
398,00	2	18	2	5	2	2	1	2
399,00	2	18	1	5	2	1	1	1
400,00	2	18	2	4	2	2	1	2
401,00	2	18	1	5	2	2	1	1
402,00	2	18	2	5	2	1	1	2
403,00	2	18	1	5	2	1	1	2
404,00	2	18	2	5	2	2	1	2
405,00	2	18	1	5	2	2	1	2
406,00	2	18	2	5	2	1	1	2
407,00	2	18	2	5	3	1	1	2
408,00	2	18	2	4	2	1	1	2
409,00	2	18	2	5	2	2	1	2
410,00	2	18	2	5	2	2	1	2
411,00	2	18	2	5	3	2	1	2
412,00	2	18	2	5	2	2	1	2
413,00	2	18	2	4	2	2	1	2
414,00	2	18	2	5	2	2	1	2
415,00	2	18	2	4	2	2	1	2
416,00	2	18	2	5	2	2	1	2
417,00	2	18	2	4	2	2	1	2

418,00	2	18	2	5	2	2	1	2
419,00	2	18	2	4	2	1	1	2
420,00	2	18	2	4	2	2	1	2
421,00	2	18	2	5	2	1	1	2
422,00	2	18	1	4	2	1	1	2
423,00	2	18	2	5	2	1	1	2
424,00	2	18	2	5	2	2	1	2
425,00	2	18	2	5	2	2	1	2
426,00	2	18	2	5	2	2	1	2
427,00	2	19	2	5	2	1	1	2
428,00	2	19	2	5	2	2	1	2
429,00	2	19	2	5	2	2	1	2
430,00	2	19	2	5	2	2	1	2
431,00	2	19	2	5	2	2	1	2
432,00	2	19	2	5	2	1	1	2
433,00	2	19	2	5	2	1	1	2
434,00	2	20	1	5	2	1	1	1
435,00	2	20	1	5	2	2	1	2
436,00	2	20	2	5	2	2	1	2
437,00	3	23	2	5	3	1	1	2
438,00	1	17	1	4	2	1	1	1
439,00	1	17	1	4	2	2	1	2
440,00	1	17	2	5	2	1	1	2
441,00	1	17	2	4	2	2	1	2
442,00	1	15	1	4	2	1	1	2
444,00	1	16	2	4	2	1	1	2
445,00	1	16	2	4	2	1	1	2
446,00	1	16	2	4	2	1	1	2
447,00	1	16	1	4	2	1	1	1
448,00	1	16	2	4	2	2	1	2
449,00	1	16	2	4	2	2	1	2
450,00	1	16	2	4	2	1	1	2

Relashion	Wihtwhom	STAY	withwhom	Highestgra	HOH_EDUC	Headhous	Headofhouse	Partneralch
2	1	1,00	3	2	3,00	1	3	0
2	5	3,00	3	3	3,00	2	3	0
2	1	1,00	5	2	3,00	2	2	0
2	5	3,00	5	2	3,00	99	1	1
2	1	1,00	3	2	3,00	99	4	1
2	1	1,00	4	4	4,00	1	1	0
2	1	1,00	3	3	3,00	99	4	0
2	1	1,00	3	5	4,00	2	1	1
3	3	2,00	2	5	4,00	1	1	0
2	3	2,00	2	4	4,00	1	2	0
2	1	1,00	3	8	#NULL!	1	1	0
2	4	3,00	3	2	3,00	1	1	0
2	3	2,00	3	4	4,00	2	99	0
2	3	2,00	2	2	3,00	1	1	0
4	5	3,00	4	1	2,00	2	4	1
2	1	1,00	3	4	4,00	1	2	0
2	5	3,00	5	2	3,00	1	1	0
2	1	1,00	3	2	3,00	2	2	0
2	1	1,00	3	2	3,00	99	4	0
2	3	2,00	2	2	3,00	2	2	0
2	1	1,00	3	2	3,00	1	1	1
2	3	2,00	3	2	3,00	2	2	0
3	1	1,00	3	8	#NULL!	2	2	1
2	4	3,00	2	8	#NULL!	1	1	1
2	2	2,00	3	1	2,00	2	2	1
2	2	2,00	1	1	2,00	99	3	0
2	1	1,00	3	8	#NULL!	1	1	0
2	5	3,00	1	8	#NULL!	1	4	0
2	5	3,00	5	6	2,00	2	2	0
2	1	1,00	1	2	3,00	1	1	1
2	2	2,00	1	6	2,00	2	2	0
1	6	3,00	3	99	#NULL!	1	1	0
3	3	2,00	2	7	1,00	2	3	1
4	1	1,00	3	2	3,00	1	1	1
2	2	2,00	3	4	4,00	2	4	0
2	4	3,00	4	6	2,00	2	3	0
2	3	2,00	2	1	2,00	2	99	1
2	3	2,00	2	3	3,00	2	1	0
1	3	2,00	2	99	#NULL!	2	3	3
2	1	1,00	3	4	4,00	1	1	1
3	5	3,00	5	2	3,00	2	2	1
2	3	2,00	2	2	3,00	1	2	0
2	1	1,00	2	3	3,00	1	1	0
2	5	3,00	2	6	2,00	1	2	0
2	4	3,00	2	5	4,00	1	1	1
2	1	1,00	4	3	3,00	1	2	0
2	1	1,00	3	4	4,00	1	1	0
2	1	1,00	3	2	3,00	1	1	0
2	1	1,00	3	5	4,00	1	1	0

2	3	2,00	2	1	2,00	2	2	0
3	1	1,00	5	8	#NULL!	1	4	0
2	5	3,00	5	5	4,00	1	1	0
2	3	2,00	3	5	4,00	2	2	1
2	4	3,00	4	1	2,00	2	2	0
2	3	2,00	2	7	1,00	2	2	0
2	1	1,00	3	8	#NULL!	99	4	0
3	5	3,00	4	3	3,00	2	4	0
2	5	3,00	5	7	1,00	99	3	0
2	1	1,00	3	2	3,00	2	1	0
2	1	1,00	3	4	4,00	1	1	0
2	1	1,00	3	2	3,00	1	1	3
3	5	3,00	2	2	3,00	2	4	0
3	1	1,00	3	1	2,00	2	1	0
2	4	3,00	4	5	4,00	1	1	0
3	1	1,00	3	6	2,00	2	2	0
3	3	2,00	3	2	3,00	1	1	1
2	3	2,00	3	4	4,00	1	4	0
3	1	1,00	3	99	#NULL!	1	1	0
3	1	1,00	3	2	3,00	2	2	3
2	1	1,00	3	5	4,00	1	1	0
2	1	1,00	3	4	4,00	1	2	1
3	4	3,00	2	8	#NULL!	99	3	0
3	2	2,00	1	2	3,00	2	1	1
3	2	2,00	1	3	3,00	2	2	0
2	4	3,00	5	6	2,00	2	4	1
2	1	1,00	5	5	4,00	2	4	0
3	1	1,00	3	3	3,00	2	2	0
2	1	1,00	3	5	4,00	1	2	0
2	1	1,00	1	1	2,00	2	2	1
2	1	1,00	3	3	3,00	1	1	0
2	3	2,00	3	5	4,00	2	99	0
2	1	1,00	3	8	#NULL!	1	2	1
2	3	2,00	5	1	2,00	2	2	1
3	1	1,00	3	3	3,00	2	1	0
2	3	2,00	3	1	2,00	1	2	0
2	1	1,00	99	8	#NULL!	99	4	0
2	1	1,00	1	5	4,00	1	1	0
2	1	1,00	3	3	3,00	1	1	0
2	3	2,00	3	3	3,00	2	2	1
2	1	1,00	3	5	4,00	1	2	0
2	5	3,00	5	8	#NULL!	1	1	0
2	5	3,00	4	4	4,00	1	1	1
4	1	1,00	3	4	4,00	2	1	1
2	3	2,00	2	3	3,00	1	4	0
2	1	1,00	3	3	3,00	1	1	0
2	3	2,00	3	4	4,00	1	1	0
2	1	1,00	3	8	#NULL!	99	4	0
4	3	2,00	2	2	3,00	1	4	0
2	1	1,00	3	5	4,00	99	4	0

2	4	3,00	4	3	3,00	2	1	0
3	1	1,00	3	4	4,00	1	1	0
2	1	1,00	3	1	2,00	1	4	3
3	1	1,00	3	4	4,00	1	1	0
2	1	1,00	3	4	4,00	1	1	0
2	1	1,00	3	1	2,00	99	99	0
2	1	1,00	3	5	4,00	1	1	0
3	5	3,00	3	8	#NULL!	1	1	0
3	4	3,00	4	8	#NULL!	2	3	0
2	2	2,00	3	1	2,00	1	2	1
3	1	1,00	3	4	4,00	1	1	0
2	1	1,00	3	7	1,00	2	2	0
2	1	1,00	3	5	4,00	2	1	1
3	1	1,00	3	4	4,00	2	4	0
2	1	1,00	3	2	3,00	1	2	1
2	1	1,00	3	1	2,00	2	2	0
2	1	1,00	2	5	4,00	1	1	0
2	1	1,00	2	3	3,00	1	1	0
3	5	3,00	1	3	3,00	1	1	1
2	1	1,00	3	1	2,00	2	3	0
2	1	1,00	3	3	3,00	1	1	0
2	1	1,00	3	2	3,00	1	2	1
2	3	2,00	3	1	2,00	2	3	0
3	5	3,00	2	2	3,00	1	1	0
2	4	3,00	4	4	4,00	1	2	1
2	1	1,00	2	4	4,00	1	1	1
2	3	2,00	2	3	3,00	1	1	1
2	3	2,00	3	99	#NULL!	99	99	3
2	5	3,00	5	4	4,00	1	1	0
3	1	1,00	3	2	3,00	2	4	0
3	1	1,00	3	4	4,00	1	4	0
3	1	1,00	3	2	3,00	1	4	0
3	3	2,00	3	99	#NULL!	1	2	0
3	1	1,00	3	2	3,00	1	1	0
3	4	3,00	3	8	#NULL!	1	1	0
3	3	2,00	2	2	3,00	1	1	0
2	1	1,00	3	7	1,00	2	1	0
3	3	2,00	5	5	4,00	1	2	3
2	3	2,00	3	2	3,00	1	2	0
2	1	1,00	3	4	4,00	1	4	0
2	5	3,00	3	3	3,00	2	4	0
3	3	2,00	2	3	3,00	2	2	0
2	1	1,00	3	4	4,00	1	4	0
2	1	1,00	3	4	4,00	1	4	0
2	3	2,00	4	2	3,00	2	2	0
2	1	1,00	4	5	4,00	1	1	0
2	3	2,00	2	5	4,00	1	1	1
3	1	1,00	3	8	#NULL!	99	99	0
1	2	2,00	1	3	3,00	1	1	1
2	1	1,00	3	3	3,00	99	99	0

2	1	1,00	3	5	4,00	2	2	1
3	3	2,00	2	5	4,00	1	1	1
2	3	2,00	2	4	4,00	2	1	0
3	1	1,00	3	3	3,00	2	4	0
3	5	3,00	5	5	4,00	1	1	0
3	3	2,00	2	2	3,00	2	2	3
2	2	2,00	5	1	2,00	1	1	1
3	1	1,00	5	6	2,00	2	2	1
3	5	3,00	2	5	4,00	1	1	0
2	1	1,00	3	5	4,00	2	1	1
3	4	3,00	4	3	3,00	1	1	0
2	3	2,00	2	99	#NULL!	1	1	1
3	3	2,00	3	8	#NULL!	1	1	0
2	1	1,00	3	7	1,00	2	2	0
2	3	2,00	2	7	1,00	2	2	0
2	2	2,00	2	5	4,00	1	1	1
3	3	2,00	2	5	4,00	2	1	0
3	1	1,00	3	3	3,00	1	1	3
3	3	2,00	3	3	3,00	2	1	0
3	3	2,00	3	4	4,00	2	1	0
2	1	1,00	3	4	4,00	1	1	0
3	1	1,00	3	2	3,00	1	1	0
2	4	3,00	3	8	#NULL!	99	4	1
3	1	1,00	3	2	3,00	2	1	1
3	3	2,00	3	2	3,00	2	1	1
2	1	1,00	4	4	4,00	1	1	3
2	1	1,00	3	3	3,00	1	1	1
3	1	1,00	3	5	4,00	1	1	0
2	3	2,00	2	5	4,00	99	4	1
3	3	2,00	3	1	2,00	1	99	0
2	3	2,00	3	5	4,00	2	2	0
2	5	3,00	1	5	4,00	1	1	0
3	2	2,00	3	5	4,00	1	1	0
2	3	2,00	3	8	#NULL!	1	1	0
2	1	1,00	2	5	4,00	1	1	0
2	1	1,00	3	99	#NULL!	1	4	0
2	1	1,00	3	3	3,00	2	3	0
2	5	3,00	2	8	#NULL!	2	1	1
3	1	1,00	3	2	3,00	1	4	0
2	1	1,00	3	4	4,00	1	1	0
2	5	3,00	5	5	4,00	1	1	0
4	5	3,00	1	4	4,00	2	1	0
4	1	1,00	3	1	2,00	1	2	0
3	3	2,00	2	3	3,00	2	1	1
2	1	1,00	3	3	3,00	1	1	0
2	3	2,00	2	8	#NULL!	1	1	1
2	3	2,00	2	2	3,00	2	2	0
2	1	1,00	3	1	2,00	2	2	0
2	1	1,00	3	2	3,00	1	1	0
2	1	1,00	3	3	3,00	1	4	0

2	3	2,00	1	3	3,00	2	4	0
3	1	1,00	3	3	3,00	2	4	0
4	4	3,00	4	5	4,00	99	4	1
2	3	2,00	2	2	3,00	2	3	0
2	1	1,00	3	5	4,00	1	1	0
2	2	2,00	3	4	4,00	1	1	0
2	4	3,00	4	4	4,00	1	1	0
2	1	1,00	2	4	4,00	1	1	0
4	3	2,00	2	3	3,00	99	4	0
4	1	1,00	3	3	3,00	1	1	0
2	2	2,00	3	3	3,00	2	2	0
3	3	2,00	2	8	#NULL!	99	4	1
4	1	1,00	3	2	3,00	2	2	3
3	4	3,00	4	8	#NULL!	99	4	0
2	3	2,00	2	2	3,00	1	4	0
3	3	2,00	3	3	3,00	2	2	0
3	5	3,00	3	8	#NULL!	99	4	3
3	3	2,00	2	2	3,00	1	1	0
3	1	1,00	3	4	4,00	1	1	0
3	5	3,00	5	8	#NULL!	99	4	0
4	2	2,00	3	2	3,00	1	1	3
4	3	2,00	3	3	3,00	2	2	1
2	1	1,00	3	8	#NULL!	1	2	0
2	1	1,00	3	1	2,00	1	1	0
3	5	3,00	4	4	4,00	1	1	1
2	3	2,00	5	2	3,00	2	2	1
2	3	2,00	4	1	2,00	2	2	0
3	1	1,00	3	5	4,00	2	2	0
2	2	2,00	2	7	1,00	99	4	0
3	1	1,00	2	99	#NULL!	1	1	3
2	1	1,00	3	5	4,00	1	1	0
2	1	1,00	3	4	4,00	1	1	1
3	5	3,00	3	5	4,00	2	2	0
2	1	1,00	3	8	#NULL!	2	3	0
2	5	3,00	4	3	3,00	2	2	0
2	3	2,00	2	2	3,00	1	1	1
2	4	3,00	4	5	4,00	1	1	0
3	4	3,00	4	2	3,00	99	4	0
3	1	1,00	3	3	3,00	2	3	0
2	1	1,00	3	4	4,00	1	1	0
2	1	1,00	5	4	4,00	1	1	0
2	3	2,00	3	8	#NULL!	1	4	0
2	1	1,00	3	2	3,00	1	1	0
2	5	3,00	4	5	4,00	99	4	0
2	4	3,00	4	1	2,00	2	3	0
2	4	3,00	4	3	3,00	2	3	0
2	3	2,00	3	5	4,00	1	1	0
2	3	2,00	3	4	4,00	1	1	0
2	5	3,00	5	3	3,00	2	2	0
2	4	3,00	1	5	4,00	2	1	0

2	1	1,00	3	2	3,00	2	1	0
2	3	2,00	2	8	#NULL!	2	4	1
2	1	1,00	3	2	3,00	1	1	0
2	4	3,00	4	2	3,00	2	3	1
2	3	2,00	3	8	#NULL!	1	4	0
2	4	3,00	4	3	3,00	2	3	1
3	3	2,00	2	5	4,00	1	1	1
2	5	3,00	2	8	#NULL!	2	3	0
2	5	3,00	5	99	#NULL!	1	4	1
2	1	1,00	3	3	3,00	2	2	0
2	3	2,00	2	2	3,00	2	2	0
3	5	3,00	5	1	2,00	1	4	0
3	1	1,00	3	3	3,00	1	1	0
2	1	1,00	3	5	4,00	2	2	1
3	1	1,00	5	1	2,00	2	2	0
3	3	2,00	3	4	4,00	1	1	0
2	1	1,00	3	4	4,00	1	1	0
3	3	2,00	3	3	3,00	1	1	0
3	4	3,00	4	2	3,00	1	4	1
3	1	1,00	2	5	4,00	1	1	0
3	1	1,00	3	4	4,00	1	1	0
3	1	1,00	3	99	#NULL!	1	1	0
3	1	1,00	3	3	3,00	1	1	0
3	1	1,00	3	2	3,00	1	1	0
3	3	2,00	3	2	3,00	1	1	0
3	1	1,00	3	99	#NULL!	1	1	3
3	5	3,00	2	4	4,00	1	1	0
3	3	2,00	2	4	4,00	1	1	0
2	1	1,00	3	8	#NULL!	1	1	0
3	1	1,00	3	2	3,00	2	2	0
2	3	2,00	2	8	#NULL!	2	3	0
2	1	1,00	2	2	3,00	1	1	0
2	3	2,00	3	8	#NULL!	2	2	0
2	4	3,00	4	8	#NULL!	2	2	0
2	4	3,00	3	8	#NULL!	99	4	0
2	5	3,00	2	3	3,00	1	1	0
3	1	1,00	3	5	4,00	2	4	3
3	2	2,00	1	2	3,00	1	1	0
2	1	1,00	3	8	#NULL!	1	1	0
3	1	1,00	3	3	3,00	1	1	0
2	1	1,00	3	8	#NULL!	1	1	0
2	4	3,00	4	8	#NULL!	2	3	0
1	3	2,00	4	3	3,00	2	2	0
2	1	1,00	3	3	3,00	99	4	0
3	1	1,00	3	8	#NULL!	2	2	0
2	1	1,00	3	4	4,00	2	2	0
2	4	3,00	4	2	3,00	2	2	0
2	1	1,00	3	2	3,00	1	1	0
2	1	1,00	3	2	3,00	2	2	0
3	1	1,00	3	3	3,00	1	1	0

3	5	3,00	3	4	4,00	1	1	0
3	1	1,00	3	3	3,00	2	2	0
3	1	1,00	5	5	4,00	1	1	0
2	5	3,00	3	5	4,00	1	1	3
2	1	1,00	3	8	#NULL!	1	4	1
2	5	3,00	5	4	4,00	2	1	0
3	1	1,00	3	8	#NULL!	1	4	1
3	3	2,00	2	3	3,00	1	1	0
3	1	1,00	3	8	#NULL!	2	4	0
3	5	3,00	1	1	2,00	1	1	0
2	3	2,00	2	5	4,00	1	1	0
3	5	3,00	3	8	#NULL!	99	1	0
3	1	1,00	3	4	4,00	1	1	3
3	1	1,00	2	5	4,00	1	1	3
3	3	2,00	2	4	4,00	2	2	0
3	1	1,00	3	4	4,00	1	1	0
2	3	2,00	3	3	3,00	1	1	1
3	1	1,00	3	4	4,00	2	1	0
3	5	3,00	5	3	3,00	2	2	0
3	1	1,00	3	1	2,00	2	1	1
2	2	2,00	3	2	3,00	1	1	0
2	5	3,00	4	3	3,00	1	1	1
3	4	3,00	2	4	4,00	1	1	3
2	4	3,00	2	4	4,00	1	1	0
3	3	2,00	1	3	3,00	1	1	0
4	3	2,00	2	5	4,00	1	1	1
2	3	2,00	3	4	4,00	1	1	0
2	1	1,00	3	2	3,00	2	2	1
2	1	1,00	3	4	4,00	1	1	1
2	4	3,00	4	8	#NULL!	2	3	1
3	3	2,00	2	4	4,00	1	1	0
2	1	1,00	3	3	3,00	2	2	0
3	1	1,00	3	3	3,00	1	1	1
2	5	3,00	4	2	3,00	2	2	0
3	5	3,00	3	3	3,00	1	1	1
3	1	1,00	3	2	3,00	1	1	0
3	1	1,00	5	4	4,00	1	1	0
2	2	2,00	1	3	3,00	1	1	1
2	1	1,00	3	4	4,00	1	1	0
2	4	3,00	4	4	4,00	2	1	0
2	5	3,00	8	1	2,00	1	2	0
3	3	2,00	3	4	4,00	1	1	0
3	1	1,00	2	4	4,00	1	1	0
3	1	1,00	3	8	#NULL!	2	2	1000
2	1	1,00	3	2	3,00	2	2	1
2	1	1,00	3	5	4,00	1	1	0
2	3	2,00	3	4	4,00	1	1	3
2	1	1,00	3	8	#NULL!	1	2	0
3	4	3,00	4	3	3,00	1	1	0
3	1	1,00	3	5	4,00	1	1	1

2	3	2,00	2	5	4,00	99	4	0
3	1	1,00	3	5	4,00	1	1	0
2	1	1,00	3	5	4,00	1	1	0
2	3	2,00	2	2	3,00	2	2	1
3	1	1,00	3	99	#NULL!	1	1	0
2	3	2,00	3	4	4,00	1	4	1
2	5	3,00	5	4	4,00	1	1	0
3	3	2,00	3	4	4,00	1	1	0
3	1	1,00	3	4	4,00	1	1	0
2	4	3,00	4	8	#NULL!	2	2	1
2	1	1,00	3	8	#NULL!	1	1	0
2	1	1,00	3	3	3,00	1	1	0
3	1	1,00	3	2	3,00	2	2	0
2	5	3,00	5	4	4,00	1	1	0
2	1	1,00	3	4	4,00	1	1	0
2	4	3,00	4	2	3,00	1	1	0
2	3	2,00	3	8	#NULL!	1	99	0
4	1	1,00	1	3	3,00	2	3	0
3	3	2,00	2	4	4,00	1	1	0
2	5	3,00	2	3	3,00	1	1	0
3	3	2,00	2	4	4,00	1	1	0
2	1	1,00	3	3	3,00	1	4	0
3	3	2,00	2	8	#NULL!	2	4	0
2	1	1,00	3	8	#NULL!	2	3	1
2	1	1,00	3	7	1,00	1	2	0
2	3	2,00	2	99	#NULL!	2	3	0
2	1	1,00	5	2	3,00	2	2	0
2	3	2,00	2	99	#NULL!	2	3	0
2	3	2,00	3	1	2,00	2	2	0
2	3	2,00	2	1	2,00	2	2	0
2	4	3,00	2	3	3,00	2	3	1
1	6	3,00	3	8	#NULL!	99	4	1
2	5	3,00	2	3	3,00	2	2	1
2	1	1,00	3	5	4,00	1	1	1
2	1	1,00	3	8	#NULL!	1	1	0
3	4	3,00	5	5	4,00	2	2	0
2	2	2,00	2	8	#NULL!	2	3	1
2	3	2,00	4	2	3,00	1	1	0
3	1	1,00	3	3	3,00	2	2	3
2	5	3,00	5	8	#NULL!	1	1	0
1	1	1,00	3	2	3,00	1	1	1
2	3	2,00	2	2	3,00	2	2	0
3	4	3,00	3	5	4,00	1	1	0
2	1	1,00	3	2	3,00	1	1	0
2	5	3,00	3	5	4,00	2	4	0
2	1	1,00	3	8	#NULL!	99	4	0
3	1	1,00	5	8	#NULL!	1	1	0
2	1	1,00	3	1	2,00	1	99	1
3	2	2,00	1	2	3,00	2	2	0
3	1	1,00	3	1	2,00	2	2	0

2	1	1,00	3	5	4,00	1	1	0
1	6	3,00	3	1	2,00	2	1	1
2	3	2,00	3	3	3,00	2	3	1
2	3	2,00	2	2	3,00	1	1	0
2	1	1,00	3	2	3,00	1	1	0
2	1	1,00	99	3	3,00	1	1	0
3	1	1,00	99	2	3,00	99	99	0
2	5	3,00	5	8	#NULL!	2	4	0
2	1	1,00	3	2	3,00	1	1	0
2	3	2,00	2	1	2,00	2	2	0
2	1	1,00	3	99	#NULL!	1	99	0
2	5	3,00	2	2	3,00	1	2	1
2	1	1,00	99	3	3,00	1	1	0
2	3	2,00	3	2	3,00	2	2	1
2	1	1,00	3	8	#NULL!	99	4	0
3	5	3,00	5	3	3,00	1	1	1
4	1	1,00	3	3	3,00	1	2	0
2	5	3,00	3	1	2,00	2	99	0
3	1	1,00	4	8	#NULL!	1	1	0
2	1	1,00	3	99	#NULL!	2	99	1
2	4	3,00	4	8	#NULL!	2	2	0
2	1	1,00	3	2	3,00	1	1	1
2	3	2,00	2	4	4,00	1	1	0
2	1	1,00	3	2	3,00	2	2	3
2	1	1,00	3	5	4,00	1	1	0
2	1	1,00	3	99	#NULL!	2	3	0
3	1	1,00	3	6	2,00	2	2	0
3	5	3,00	2	1	2,00	2	2	0
3	3	2,00	3	1	2,00	1	2	0
2	1	1,00	3	4	4,00	1	1	0
2	1	1,00	3	2	3,00	2	2	0
2	1	1,00	3	1	2,00	2	2	0

partnerem	yearspartn	Believemar	Menhaving	encorauger	Familydecis	Moreimpo	Boysarebet	Girlsconcer
7	1	2	2	2	2	2,00	2,00	2,00
7	5	2	1	1	1	2,00	2,00	1,00
1	1	2	2	2	1	2,00	2,00	2,00
7	5	2	2	2	2	2,00	2,00	2,00
7	1	2	2	2	2	2,00	2,00	2,00
7	1	1	2	1	1	2,00	2,00	2,00
7	5	1	2	2	2	2,00	2,00	2,00
7	5	2	2	2	2	2,00	2,00	2,00
7	5	2	2	2	1	2,00	2,00	2,00
7	5	1	2	1	2	2,00	2,00	2,00
7	1	2	2	2	2	2,00	2,00	2,00
7	2	2	2	2	2	2,00	2,00	2,00
7	1	2	1	1	2	2,00	2,00	2,00
1	1	2	1	2	2	2,00	2,00	2,00
7	2	2	2	2	1	2,00	2,00	2,00
7	1	99	2	2	1	2,00	2,00	1,00
7	5	2	2	2	2	2,00	2,00	2,00
7	5	2	2	2	1	2,00	2,00	2,00
7	5	2	1	2	2	2,00	2,00	2,00
7	1	2	2	2	1	2,00	2,00	2,00
1	1	2	2	2	2	2,00	2,00	2,00
7	5	2	2	2	2	2,00	2,00	2,00
7	5	1	2	2	2	2,00	2,00	2,00
1	1	2	2	2	2	2,00	2,00	2,00
7	1	2	2	2	1	2,00	2,00	2,00
7	1	2	2	2	2	2,00	2,00	2,00
7	1	2	2	2	1	2,00	2,00	2,00
1	5	2	2	2	2	2,00	2,00	2,00
7	5	2	2	2	1	2,00	2,00	1,00
7	1	2	2	2	2	2,00	2,00	2,00
1	5	2	1	2	2	1,00	2,00	2,00
1	99	1	2	2	1	2,00	2,00	2,00
7	2	22	2	2	2	2,00	2,00	2,00
1	1	1	1	2	1	2,00	2,00	2,00
1	1	2	2	2	1	2,00	2,00	2,00
7	5	2	2	2	2	2,00	2,00	2,00
1	5	2	2	2	2	2,00	2,00	2,00
7	99	1	1	1	1	2,00	1,00	2,00
7	99	2	2	2	2	2,00	2,00	2,00
7	1	1	2	2	2	2,00	2,00	2,00
1	1	1	1	2	2	2,00	2,00	2,00
1	1	2	1	1	1	2,00	2,00	2,00
1	2	2	1	2	2	2,00	2,00	1,00
7	5	2	2	2	1	2,00	1,00	2,00
1	1	2	2	2	2	2,00	2,00	2,00
1	1	2	2	2	2	2,00	2,00	2,00
7	5	2	2	2	2	2,00	2,00	2,00
7	1	2	2	2	2	2,00	2,00	2,00
7	5	2	2	2	1	2,00	2,00	2,00

7	1	2	2	2	2	2,00	2,00	2,00
7	1	2	2	2	2	2,00	2,00	2,00
7	1	1	1	2	1	2,00	2,00	2,00
7	5	2	2	1	2	2,00	2,00	2,00
1	5	2	2	2	2	2,00	2,00	2,00
7	2	2	2	2	2	2,00	2,00	2,00
1	5	2	2	1	1	2,00	2,00	2,00
7	5	2	2	2	1	2,00	2,00	2,00
7	5	2	2	2	2	2,00	2,00	2,00
7	5	2	2	2	2	2,00	2,00	2,00
7	1	2	2	2	2	2,00	2,00	2,00
7	4	1	2	2	1	2,00	2,00	1,00
7	1	2	2	1	1	2,00	1,00	1,00
1	5	1	2	1	1	1,00	1,00	1,00
7	1	99	2	99	1	2,00	2,00	2,00
7	99	2	1	2	2	2,00	2,00	2,00
7	1	2	2	2	2	2,00	2,00	2,00
7	1	2	2	2	2	2,00	2,00	2,00
7	5	1	2	2	1	2,00	2,00	2,00
7	1	2	2	2	2	2,00	2,00	2,00
7	5	2	2	2	1	2,00	1,00	2,00
7	5	1	99	99	99	99,00	2,00	1,00
7	4	2	2	99	2	2,00	2,00	2,00
1	2	2	2	2	2	2,00	2,00	2,00
7	1	2	2	2	2	2,00	2,00	2,00
7	5	1	2	2	1	2,00	2,00	2,00
7	5	2	2	2	2	2,00	2,00	2,00
7	1	1	1	1	2	2,00	2,00	2,00
1	1	1	2	1	1	2,00	2,00	2,00
1	5	2	2	2	2	2,00	2,00	2,00
1	1	1	2	2	1	2,00	1,00	2,00
7	99	2	1	2	1	2,00	2,00	1,00
7	1	2	2	2	1	2,00	2,00	2,00
7	5	2	2	1	2	2,00	2,00	2,00
7	5	1	2	2	2	2,00	2,00	2,00
7	1	2	2	2	2	2,00	2,00	2,00
5	99	1	2	2	1	1,00	2,00	2,00
7	5	2	2	2	2	2,00	2,00	2,00
7	5	2	2	2	1	2,00	1,00	2,00
7	5	2	1	2	2	2,00	2,00	2,00
7	1	2	1	2	2	2,00	2,00	2,00
1	1	1	2	2	1	1,00	1,00	2,00
7	5	2	2	2	2	2,00	2,00	2,00
1	1	2	2	2	2	2,00	2,00	2,00
7	1	1	2	2	2	2,00	2,00	2,00
7	5	2	2	2	2	2,00	2,00	2,00
7	5	2	2	2	2	2,00	2,00	2,00
7	5	1	1	2	1	2,00	2,00	1,00
7	5	2	1	2	2	2,00	2,00	2,00
7	5	2	2	2	2	2,00	2,00	2,00

7	5	2	2	1	2	2,00	2,00	2,00
7	5	1	2	2	2	2,00	2,00	2,00
7	5	2	2	2	1	2,00	2,00	2,00
7	1	2	2	2	1	2,00	2,00	2,00
1	2	2	1	2	2	2,00	2,00	2,00
7	5	2	2	2	1	2,00	2,00	2,00
7	5	2	2	2	2	2,00	2,00	2,00
7	4	2	2	1	2	2,00	2,00	1,00
7	1	2	2	2	1	2,00	2,00	2,00
7	1	2	2	2	1	2,00	2,00	2,00
5	4	1	2	2	2	2,00	2,00	2,00
7	1	1	1	2	1	2,00	1,00	2,00
7	5	2	2	2	2	2,00	2,00	2,00
7	1	2	2	2	1	2,00	2,00	2,00
7	1	2	2	2	2	2,00	2,00	2,00
7	5	2	2	2	1	2,00	2,00	2,00
7	1	2	2	1	2	2,00	2,00	2,00
7	5	2	2	2	1	2,00	2,00	2,00
7	1	2	2	2	2	2,00	2,00	2,00
1	1	1	2	2	2	1,00	2,00	2,00
1	1	1	2	2	2	2,00	2,00	2,00
1	1	2	2	2	2	2,00	2,00	2,00
7	1	1	2	2	1	2,00	2,00	2,00
7	5	2	2	2	2	2,00	2,00	2,00
1	1	2	2	2	2	2,00	2,00	2,00
7	1	1	2	2	2	2,00	2,00	2,00
1	1	2	2	2	2	2,00	2,00	2,00
1	1	1	2	2	1	2,00	2,00	2,00
7	1	2	2	2	2	2,00	2,00	2,00
7	1	2	2	2	2	2,00	2,00	2,00
7	1	2	2	1	2	2,00	2,00	2,00
7	1	2	2	2	2	2,00	2,00	2,00
7	5	2	2	2	1	2,00	2,00	2,00
7	4	2	2	2	2	2,00	2,00	1,00
7	1	2	2	2	1	2,00	2,00	2,00
7	1	1	2	2	2	1,00	2,00	2,00
7	5	2	1	2	2	2,00	2,00	2,00
7	5	2	2	2	2	2,00	2,00	2,00
7	1	2	2	2	2	2,00	2,00	2,00
7	5	1	2	2	1	2,00	1,00	2,00
7	1	2	2	2	2	2,00	2,00	2,00
5	5	2	2	2	1	2,00	2,00	2,00
7	5	1	2	2	1	2,00	2,00	2,00
7	5	1	2	1	1	1,00	2,00	2,00
1	1	2	2	2	2	2,00	2,00	2,00
7	1	2	2	2	2	2,00	2,00	2,00
1	2	1	2	2	2	2,00	2,00	2,00
7	1	2	2	2	1	2,00	2,00	2,00
7	5	1	2	2	2	1,00	2,00	2,00
7	5	1	2	2	2	2,00	2,00	2,00

7	5	2	2	2	1	2,00	2,00	2,00
7	1	2	2	2	2	2,00	2,00	2,00
7	1	1	2	2	2	2,00	2,00	2,00
7	1	2	2	2	2	2,00	2,00	2,00
1	1	2	1	2	2	2,00	2,00	2,00
1	1	2	1	2	2	2,00	2,00	2,00
7	4	2	1	2	2	1,00	1,00	1,00
7	1	1	1	2	2	2,00	2,00	2,00
7	1	1	2	2	2	2,00	2,00	2,00
7	1	2	2	2	1	2,00	2,00	2,00
7	5	2	2	2	1	2,00	2,00	2,00
7	5	1	1	2	2	2,00	2,00	2,00
7	5	1	2	2	1	2,00	2,00	2,00
7	1	2	2	2	1	2,00	2,00	2,00
7	5	1	2	2	1	1,00	1,00	2,00
7	1	2	2	2	2	2,00	2,00	2,00
7	5	2	2	2	2	2,00	2,00	2,00
5	5	1	2	2	1	2,00	2,00	2,00
7	4	2	2	2	2	2,00	2,00	2,00
7	1	2	2	2	2	2,00	2,00	2,00
7	1	2	2	1	2	2,00	2,00	1,00
7	4	2	2	2	1	2,00	2,00	2,00
7	5	1	2	2	2	2,00	2,00	2,00
7	5	2	2	2	2	2,00	2,00	2,00
7	5	2	2	2	2	2,00	2,00	2,00
7	5	2	2	2	2	2,00	2,00	2,00
7	1	2	1	1	2	2,00	1,00	1,00
7	5	2	1	2	2	2,00	2,00	2,00
7	1	2	2	2	1	2,00	2,00	2,00
7	1	2	2	2	1	2,00	2,00	2,00
7	5	2	1	2	2	2,00	2,00	2,00
7	5	2	2	1	2	2,00	2,00	2,00
7	1	2	2	2	1	2,00	2,00	2,00
7	5	2	2	2	2	2,00	2,00	2,00
7	4	2	2	2	2	2,00	2,00	2,00
7	1	2	2	2	2	2,00	2,00	2,00
1	2	2	2	2	1	2,00	2,00	2,00
7	4	2	2	2	2	2,00	2,00	2,00
7	5	2	2	2	2	2,00	2,00	2,00
7	1	2	2	2	1	2,00	2,00	2,00
1	1	1	2	2	1	2,00	2,00	2,00
1	1	2	2	2	2	2,00	2,00	2,00
7	5	2	2	2	2	2,00	2,00	2,00
7	5	1	1	2	2	2,00	1,00	2,00
7	1	2	2	2	2	2,00	2,00	2,00
7	5	2	2	2	2	2,00	2,00	2,00
7	5	2	2	2	2	2,00	2,00	2,00
7	5	2	2	2	1	2,00	2,00	2,00
1	5	2	1	2	1	2,00	2,00	2,00
7	5	2	2	2	1	2,00	2,00	2,00

7	5	2	2	1	2	2,00	2,00	2,00
5	4	2	2	2	2	2,00	2,00	1,00
7	1	1	2	2	1	2,00	2,00	2,00
7	1	1	2	2	2	2,00	2,00	2,00
7	5	2	2	2	2	2,00	2,00	2,00
7	1	2	2	2	2	2,00	2,00	2,00
7	1	2	2	2	2	2,00	2,00	2,00
7	1	2	2	2	2	2,00	2,00	2,00
7	5	2	2	2	2	2,00	2,00	2,00
7	5	2	2	1	2	2,00	2,00	2,00
7	5	1	2	2	2	2,00	2,00	2,00
7	4	2	1	2	2	2,00	2,00	2,00
5	4	2	2	2	2	2,00	2,00	2,00
5	5	1	2	2	2	2,00	2,00	2,00
7	1	2	2	2	2	2,00	2,00	2,00
7	5	2	2	2	2	2,00	2,00	2,00
5	4	1	1	2	2	2,00	2,00	2,00
1	1	2	2	2	2	2,00	1,00	2,00
7	5	2	1	2	1	2,00	2,00	1,00
7	5	2	2	2	2	2,00	2,00	2,00
7	5	2	2	2	2	2,00	2,00	2,00
7	5	1	2	2	1	2,00	2,00	2,00
7	1	2	2	2	2	2,00	2,00	2,00
7	1	2	2	1	1	2,00	2,00	2,00
7	5	2	2	2	2	2,00	2,00	2,00
7	5	1	2	2	1	1,00	2,00	2,00
1	1	2	2	2	2	2,00	2,00	2,00
7	1	2	2	1	1	2,00	2,00	2,00
7	1	2	1	1	2	2,00	2,00	2,00
5	4	2	2	2	1	2,00	2,00	2,00
1	1	2	2	2	1	2,00	2,00	1,00
7	1	2	2	1	1	2,00	2,00	1,00
7	5	1	2	2	1	2,00	2,00	2,00
7	5	1	1	2	1	2,00	1,00	1,00
7	2	2	2	2	2	2,00	2,00	2,00
7	5	2	2	2	2	2,00	2,00	2,00
7	5	2	2	1	1	1,00	1,00	2,00
7	5	2	2	2	2	1,00	2,00	2,00
7	1	2	2	2	2	2,00	2,00	2,00
7	1	2	2	1	1	2,00	2,00	2,00
7	5	2	2	2	2	2,00	2,00	2,00
7	1	2	2	2	2	2,00	2,00	2,00
7	1	2	2	2	1	2,00	1,00	2,00
7	5	2	2	2	1	2,00	2,00	2,00
7	5	2	2	2	1	2,00	2,00	2,00
7	1	2	2	2	2	2,00	2,00	2,00
7	5	1	2	1	1	1,00	1,00	2,00
7	1	2	2	2	2	2,00	2,00	2,00
7	1	1	2	2	1	2,00	1,00	2,00
7	5	1	2	2	2	2,00	2,00	2,00

7	1	2	2	2	2	2,00	2,00	2,00
7	1	1	2	2	1	2,00	2,00	2,00
7	5	2	2	2	1	2,00	2,00	1,00
7	5	2	2	2	2	2,00	2,00	2,00
1	5	2	2	2	1	2,00	2,00	2,00
7	5	1	2	1	2	1,00	2,00	1,00
1	1	2	2	2	2	2,00	2,00	2,00
7	5	2	2	2	1	2,00	2,00	2,00
7	5	2	2	2	2	2,00	2,00	2,00
7	5	2	2	2	2	2,00	2,00	2,00
7	1	1	2	2	2	2,00	2,00	2,00
7	1	2	2	1	2	1,00	2,00	2,00
7	1	2	2	2	1	2,00	2,00	1,00
1	1	2	2	1	2	1,00	2,00	2,00
1	1	2	2	2	2	2,00	2,00	2,00
7	5	2	2	2	1	2,00	2,00	2,00
7	1	2	2	2	2	2,00	2,00	2,00
7	1	2	2	2	2	2,00	2,00	2,00
7	1	2	2	2	1	2,00	2,00	2,00
7	4	2	2	1	2	2,00	2,00	1,00
7	1	2	2	2	2	2,00	2,00	2,00
7	1	2	2	2	2	2,00	2,00	2,00
7	5	2	1	1	1	1,00	2,00	1,00
7	1	1	2	2	2	2,00	2,00	2,00
7	1	2	2	2	2	2,00	2,00	2,00
7	1	2	2	2	2	2,00	2,00	2,00
7	1	2	2	2	2	2,00	2,00	2,00
7	4	2	2	2	2	2,00	2,00	2,00
1	1	2	2	2	2	2,00	2,00	2,00
7	1	2	2	2	1	2,00	2,00	2,00
7	5	2	2	2	2	2,00	2,00	2,00
7	5	2	2	2	1	2,00	1,00	2,00
1	1	2	2	2	2	2,00	2,00	2,00
7	2	2	2	2	2	2,00	2,00	2,00
7	4	2	2	2	2	2,00	2,00	2,00
7	1	2	1	2	1	2,00	2,00	2,00
7	5	1	2	2	2	2,00	2,00	2,00
7	5	2	2	2	2	2,00	2,00	2,00
7	1	2	1	2	1	2,00	1,00	2,00
1	5	2	2	2	2	2,00	2,00	2,00
1	5	1	2	2	1	2,00	1,00	2,00
1	5	1	2	2	1	2,00	1,00	2,00
7	1	1	1	2	2	2,00	2,00	2,00
7	4	2	2	2	2	2,00	2,00	2,00
7	4	2	2	2	2	2,00	2,00	1,00
7	4	2	2	2	1	2,00	2,00	2,00
1	1	1	2	2	1	2,00	2,00	2,00
7	1	2	2	2	1	2,00	2,00	2,00
7	5	2	2	1	2	2,00	2,00	2,00
7	1	2	2	2	2	2,00	2,00	2,00

7	1	2	2	2	2	2,00	2,00	2,00
7	5	2	2	2	1	2,00	2,00	2,00
7	5	1	2	2	2	2,00	2,00	2,00
7	5	2	2	2	2	2,00	2,00	2,00
7	1	2	2	2	2	2,00	2,00	2,00
7	5	2	2	2	2	2,00	2,00	2,00
7	5	2	2	2	2	2,00	2,00	2,00
7	1	2	2	2	2	2,00	2,00	2,00
7	5	2	2	2	2	2,00	2,00	2,00
7	1	1	2	2	2	2,00	2,00	2,00
7	5	2	2	2	2	2,00	2,00	2,00
7	5	2	2	2	2	2,00	2,00	2,00
7	5	2	2	2	2	2,00	2,00	2,00
7	5	2	2	2	2	2,00	2,00	2,00
7	5	2	2	2	2	2,00	2,00	2,00
7	5	2	2	2	1	2,00	1,00	2,00
1	2	2	2	2	2	2,00	2,00	2,00
7	1	2	2	2	2	1,00	2,00	2,00
7	5	2	2	2	2	2,00	2,00	2,00
7	1	1	2	2	1	2,00	2,00	2,00
7	1	2	2	2	2	2,00	2,00	2,00
7	1	2	2	2	2	2,00	2,00	2,00
5	4	2	2	2	2	2,00	2,00	2,00
7	5	2	2	2	2	2,00	2,00	2,00
7	5	1	2	2	2	2,00	2,00	2,00
7	5	2	2	2	2	2,00	2,00	2,00
7	5	2	2	2	2	2,00	2,00	2,00
1	1	2	2	2	1	2,00	2,00	2,00
7	1	2	2	2	1	2,00	2,00	2,00
7	1	2	2	2	2	2,00	1,00	2,00
7	5	2	2	1	2	2,00	2,00	2,00
7	5	2	2	2	2	2,00	2,00	2,00
7	5	2	2	2	2	2,00	2,00	1,00
7	1	2	2	2	2	2,00	2,00	2,00
7	5	2	2	2	1	2,00	2,00	2,00
7	5	2	1	2	2	2,00	2,00	2,00
7	1	2	2	2	2	2,00	2,00	2,00
1	2	1	2	2	1	2,00	2,00	2,00
1	1	2	2	2	2	2,00	2,00	2,00
7	5	2	2	2	1	2,00	2,00	2,00
7	5	2	2	2	2	2,00	2,00	2,00
7	1	2	2	2	2	2,00	2,00	2,00
7	5	2	2	2	2	2,00	2,00	2,00
1000	1000	2	2	2	1	2,00	2,00	2,00
7	1	2	2	2	2	2,00	2,00	2,00
7	5	2	2	2	2	2,00	2,00	2,00
7	5	1	2	1	2	2,00	2,00	2,00
7	1	2	2	2	1	2,00	2,00	2,00
7	5	2	2	2	2	2,00	2,00	2,00
7	1	2	2	2	2	2,00	2,00	2,00

7	5	1	2	1	1	2,00	2,00	2,00
99	1	2	2	2	2	2,00	2,00	2,00
7	5	2	2	2	2	2,00	2,00	2,00
7	1	2	2	2	1	2,00	2,00	2,00
7	5	2	1	2	2	2,00	2,00	2,00
1	1	2	2	2	1	2,00	1,00	2,00
7	5	1	2	2	2	2,00	2,00	2,00
7	5	2	2	2	2	2,00	2,00	2,00
7	5	1	2	2	1	2,00	2,00	2,00
7	5	2	2	2	1	2,00	2,00	2,00
7	1	2	2	2	2	2,00	2,00	2,00
7	5	2	2	2	2	2,00	2,00	2,00
1	5	2	2	2	2	2,00	2,00	2,00
7	5	2	2	2	2	2,00	2,00	2,00
7	1	2	2	2	1	2,00	2,00	2,00
7	5	2	2	2	1	2,00	2,00	2,00
7	5	1	2	2	2	2,00	2,00	2,00
7	5	2	2	2	2	2,00	2,00	2,00
7	5	2	2	2	2	2,00	2,00	1,00
7	5	2	2	2	2	2,00	2,00	2,00
7	5	2	2	2	2	2,00	2,00	2,00
7	5	1	2	2	1	1,00	2,00	1,00
7	5	1	2	2	1	2,00	2,00	2,00
7	5	2	2	2	2	2,00	2,00	2,00
7	1	2	1	2	2	2,00	2,00	2,00
7	5	2	2	2	2	2,00	2,00	2,00
7	1	2	2	2	2	2,00	2,00	2,00
7	1	1	1	1	1	1,00	1,00	1,00
1	1	2	2	2	1	2,00	2,00	2,00
7	5	1	2	2	1	2,00	2,00	2,00
1	1	1	2	2	2	2,00	2,00	2,00
7	2	1	2	2	2	2,00	2,00	2,00
7	1	2	2	2	2	2,00	2,00	2,00
7	5	2	2	2	2	2,00	2,00	2,00
7	1	2	2	2	2	2,00	2,00	2,00
7	5	2	2	2	2	2,00	2,00	2,00
7	1	2	2	2	2	2,00	2,00	2,00
7	5	2	2	2	2	2,00	2,00	2,00
7	1	2	2	2	2	2,00	2,00	2,00
7	5	2	1	2	1	2,00	2,00	2,00
7	1	1	2	2	1	2,00	2,00	2,00
7	1	2	1	2	2	2,00	2,00	2,00
7	5	2	2	2	1	2,00	2,00	2,00
7	5	1	1	2	2	2,00	2,00	2,00
7	1	2	2	2	2	2,00	2,00	2,00
1	4	2	2	2	1	2,00	2,00	2,00
1	5	2	2	2	2	2,00	2,00	2,00
7	1	2	2	2	2	2,00	1,00	2,00
7	5	2	2	1	2	2,00	2,00	2,00
7	1	1	2	2	1	2,00	2,00	2,00

7	5	2	2	2	2	2,00	2,00	2,00
7	5	2	2	2	1	2,00	1,00	1,00
7	5	2	2	2	2	2,00	2,00	2,00
7	5	2	2	2	1	2,00	2,00	2,00
7	1	1	2	2	1	2,00	2,00	2,00
7	1	1	2	2	2	2,00	2,00	2,00
7	1	2	2	2	2	2,00	2,00	1,00
7	1	2	2	2	2	2,00	2,00	2,00
7	5	2	2	2	2	2,00	2,00	2,00
1	1	1	2	2	2	2,00	2,00	2,00
7	1	1	2	1	1	1,00	1,00	1,00
7	1	2	2	2	2	2,00	2,00	2,00
7	1	2	2	2	2	2,00	2,00	2,00
7	5	2	2	2	2	2,00	2,00	2,00
7	5	1	2	2	2	2,00	2,00	2,00
7	5	2	2	2	2	2,00	2,00	2,00
7	1	2	2	2	2	2,00	2,00	2,00
7	4	2	1	2	1	2,00	2,00	2,00
7	2	2	2	2	2	2,00	2,00	2,00
7	1	2	2	2	2	2,00	2,00	2,00
7	1	1	2	2	2	2,00	2,00	2,00
7	2	1	2	2	2	2,00	1,00	2,00
7	5	2	2	2	2	2,00	2,00	2,00
7	5	2	2	2	1	2,00	1,00	2,00
7	5	1	2	2	2	2,00	2,00	2,00
7	1	1	1	2	1	2,00	1,00	2,00
7	4	2	2	2	1	2,00	2,00	2,00
7	1	2	2	2	2	2,00	2,00	2,00
7	1	2	2	2	2	2,00	2,00	2,00
99	4	2	2	2	2	2,00	2,00	2,00
7	5	1	2	2	1	2,00	1,00	2,00
7	5	2	2	2	2	2,00	2,00	2,00

womensho	violenceim	Womendes	womenmal	violenceby	violenceisw	womenmu	violenceisp	manhasrigt
2,00	2,00	2,00	2,00	2,00	2,00	2,00	2,00	2,00
1,00	1,00	2,00	1,00	1,00	1,00	1,00	1,00	1,00
1,00	2,00	2,00	2,00	2,00	2,00	2,00	2,00	2,00
2,00	2,00	2,00	2,00	2,00	2,00	2,00	2,00	2,00
2,00	2,00	2,00	1,00	1,00	1,00	2,00	2,00	2,00
2,00	2,00	2,00	2,00	2,00	2,00	2,00	2,00	2,00
2,00	2,00	2,00	2,00	2,00	2,00	2,00	1,00	2,00
1,00	2,00	2,00	2,00	2,00	2,00	2,00	2,00	2,00
2,00	2,00	2,00	2,00	2,00	2,00	2,00	2,00	2,00
2,00	2,00	2,00	2,00	2,00	1,00	2,00	1,00	2,00
2,00	2,00	2,00	2,00	2,00	2,00	2,00	2,00	2,00
2,00	2,00	2,00	2,00	2,00	2,00	2,00	2,00	2,00
2,00	2,00	2,00	2,00	1,00	2,00	2,00	1,00	2,00
2,00	2,00	2,00	2,00	2,00	2,00	2,00	1,00	2,00
2,00	2,00	2,00	2,00	2,00	2,00	2,00	2,00	2,00
2,00	2,00	2,00	2,00	2,00	2,00	2,00	2,00	2,00
2,00	2,00	2,00	2,00	2,00	2,00	2,00	2,00	2,00
2,00	2,00	2,00	2,00	1,00	1,00	2,00	1,00	2,00
2,00	2,00	2,00	2,00	2,00	2,00	2,00	2,00	2,00
2,00	2,00	2,00	2,00	2,00	2,00	2,00	2,00	2,00
1,00	2,00	2,00	2,00	2,00	2,00	2,00	2,00	2,00
2,00	2,00	2,00	2,00	2,00	2,00	2,00	2,00	2,00
2,00	2,00	2,00	2,00	1,00	2,00	2,00	1,00	2,00
2,00	2,00	2,00	2,00	2,00	2,00	2,00	2,00	2,00
2,00	2,00	2,00	2,00	2,00	1,00	2,00	1,00	2,00
2,00	2,00	2,00	2,00	2,00	99,00	2,00	2,00	2,00
2,00	2,00	2,00	2,00	2,00	2,00	2,00	2,00	2,00
2,00	2,00	2,00	2,00	2,00	2,00	2,00	2,00	2,00
2,00	1,00	1,00	2,00	2,00	2,00	2,00	1,00	2,00
2,00	2,00	2,00	2,00	2,00	2,00	2,00	2,00	2,00
1,00	2,00	2,00	2,00	2,00	2,00	2,00	1,00	2,00
2,00	2,00	2,00	2,00	2,00	2,00	2,00	2,00	2,00
2,00	2,00	2,00	2,00	2,00	2,00	2,00	2,00	2,00
1,00	2,00	2,00	2,00	2,00	2,00	2,00	2,00	2,00
2,00	2,00	2,00	2,00	2,00	2,00	2,00	2,00	2,00
2,00	2,00	2,00	2,00	2,00	2,00	2,00	2,00	2,00
2,00	2,00	2,00	2,00	2,00	2,00	2,00	2,00	2,00
2,00	1,00	2,00	1,00	2,00	1,00	2,00	1,00	2,00
2,00	2,00	2,00	2,00	2,00	2,00	2,00	2,00	2,00
2,00	2,00	2,00	2,00	2,00	2,00	2,00	2,00	2,00
2,00	2,00	2,00	2,00	2,00	2,00	2,00	2,00	2,00
2,00	2,00	2,00	2,00	2,00	2,00	2,00	1,00	2,00
1,00	1,00	1,00	1,00	1,00	1,00	1,00	1,00	1,00
1,00	2,00	2,00	2,00	2,00	2,00	2,00	1,00	2,00
2,00	2,00	2,00	2,00	2,00	2,00	2,00	2,00	2,00
2,00	2,00	2,00	2,00	2,00	2,00	2,00	2,00	2,00
2,00	2,00	2,00	2,00	2,00	1,00	2,00	2,00	2,00
2,00	2,00	2,00	2,00	2,00	2,00	2,00	2,00	2,00
2,00	2,00	2,00	2,00	2,00	2,00	2,00	2,00	2,00

2,00	2,00	2,00	2,00	2,00	2,00	2,00	2,00	2,00
2,00	2,00	2,00	2,00	2,00	2,00	2,00	2,00	2,00
2,00	2,00	2,00	2,00	2,00	2,00	2,00	2,00	2,00
2,00	2,00	2,00	2,00	2,00	2,00	2,00	2,00	2,00
2,00	2,00	2,00	2,00	2,00	2,00	2,00	2,00	2,00
2,00	2,00	2,00	2,00	2,00	2,00	2,00	2,00	2,00
2,00	2,00	2,00	2,00	2,00	2,00	2,00	2,00	2,00
2,00	2,00	2,00	2,00	2,00	2,00	2,00	2,00	2,00
2,00	2,00	2,00	2,00	2,00	2,00	2,00	2,00	2,00
2,00	2,00	2,00	2,00	2,00	2,00	2,00	2,00	2,00
2,00	2,00	2,00	2,00	2,00	2,00	2,00	2,00	2,00
2,00	2,00	2,00	2,00	1,00	2,00	2,00	2,00	2,00
2,00	2,00	2,00	2,00	2,00	1,00	1,00	1,00	1,00
1,00	2,00	2,00	2,00	1,00	1,00	1,00	1,00	1,00
2,00	2,00	2,00	2,00	2,00	1,00	2,00	2,00	2,00
2,00	1,00	2,00	2,00	2,00	2,00	2,00	2,00	2,00
2,00	2,00	2,00	2,00	2,00	2,00	2,00	2,00	2,00
2,00	2,00	2,00	2,00	2,00	2,00	2,00	2,00	2,00
2,00	2,00	2,00	2,00	2,00	2,00	2,00	2,00	2,00
2,00	2,00	2,00	2,00	2,00	2,00	2,00	2,00	2,00
2,00	2,00	2,00	2,00	2,00	2,00	2,00	2,00	2,00
2,00	1,00	2,00	2,00	2,00	2,00	2,00	1,00	2,00
2,00	2,00	2,00	2,00	1,00	2,00	2,00	1,00	2,00
1,00	1,00	2,00	1,00	2,00	2,00	2,00	1,00	2,00
2,00	2,00	2,00	2,00	2,00	2,00	2,00	2,00	2,00
2,00	2,00	2,00	2,00	2,00	2,00	2,00	2,00	2,00
2,00	2,00	2,00	2,00	2,00	2,00	2,00	2,00	2,00
2,00	2,00	2,00	2,00	2,00	2,00	2,00	2,00	2,00
1,00	2,00	2,00	2,00	2,00	2,00	2,00	2,00	2,00
2,00	2,00	2,00	2,00	2,00	2,00	2,00	2,00	2,00
2,00	2,00	2,00	2,00	2,00	2,00	2,00	2,00	2,00
1,00	1,00	2,00	2,00	1,00	2,00	2,00	1,00	2,00
2,00	2,00	2,00	2,00	2,00	2,00	2,00	2,00	2,00
2,00	2,00	2,00	2,00	2,00	2,00	2,00	2,00	2,00
2,00	2,00	2,00	1,00	2,00	2,00	2,00	2,00	2,00
2,00	2,00	2,00	2,00	2,00	2,00	2,00	2,00	2,00
2,00	1,00	1,00	2,00	2,00	2,00	1,00	1,00	1,00
2,00	2,00	2,00	2,00	2,00	2,00	2,00	2,00	2,00
2,00	1,00	2,00	2,00	1,00	2,00	2,00	1,00	2,00
2,00	2,00	2,00	1,00	2,00	2,00	2,00	1,00	2,00
2,00	2,00	1,00	1,00	2,00	2,00	2,00	1,00	2,00
2,00	2,00	2,00	2,00	2,00	2,00	2,00	2,00	2,00
2,00	2,00	2,00	2,00	2,00	2,00	2,00	2,00	2,00
2,00	2,00	2,00	2,00	2,00	2,00	2,00	2,00	2,00
2,00	2,00	2,00	2,00	2,00	2,00	2,00	2,00	1,00
2,00	2,00	2,00	2,00	2,00	2,00	2,00	2,00	2,00
2,00	2,00	2,00	2,00	2,00	2,00	2,00	2,00	2,00
2,00	2,00	2,00	2,00	2,00	2,00	2,00	1,00	2,00
2,00	2,00	2,00	2,00	2,00	2,00	2,00	1,00	2,00
2,00	2,00	2,00	2,00	2,00	2,00	2,00	2,00	2,00

2,00	2,00	2,00	2,00	2,00	2,00	2,00	2,00	2,00
2,00	2,00	2,00	2,00	2,00	2,00	2,00	2,00	2,00
2,00	2,00	2,00	2,00	1,00	2,00	2,00	2,00	2,00
2,00	2,00	2,00	2,00	2,00	2,00	2,00	2,00	2,00
2,00	2,00	2,00	2,00	2,00	2,00	2,00	2,00	2,00
2,00	2,00	2,00	2,00	2,00	2,00	2,00	2,00	2,00
2,00	2,00	2,00	2,00	2,00	2,00	2,00	2,00	2,00
2,00	2,00	2,00	2,00	2,00	2,00	2,00	2,00	2,00
2,00	2,00	2,00	2,00	2,00	2,00	2,00	2,00	2,00
2,00	2,00	2,00	2,00	2,00	2,00	2,00	2,00	2,00
2,00	2,00	2,00	2,00	2,00	2,00	2,00	2,00	2,00
2,00	2,00	2,00	1,00	2,00	2,00	2,00	1,00	2,00
2,00	2,00	2,00	2,00	2,00	2,00	2,00	2,00	2,00
2,00	2,00	2,00	1,00	1,00	1,00	1,00	1,00	1,00
2,00	2,00	2,00	2,00	2,00	2,00	2,00	2,00	2,00
2,00	2,00	2,00	1,00	2,00	1,00	2,00	2,00	2,00
2,00	1,00	2,00	1,00	1,00	1,00	2,00	2,00	2,00
2,00	2,00	2,00	1,00	2,00	1,00	2,00	2,00	2,00
2,00	2,00	2,00	2,00	2,00	2,00	2,00	2,00	2,00
1,00	2,00	2,00	2,00	1,00	2,00	2,00	2,00	2,00
2,00	2,00	1,00	2,00	2,00	2,00	2,00	2,00	2,00
2,00	2,00	2,00	2,00	2,00	2,00	2,00	2,00	2,00
2,00	2,00	2,00	2,00	2,00	2,00	2,00	2,00	1,00
2,00	2,00	2,00	2,00	2,00	2,00	2,00	2,00	2,00
2,00	2,00	2,00	1,00	2,00	2,00	2,00	2,00	2,00
2,00	2,00	2,00	2,00	2,00	2,00	2,00	2,00	2,00
2,00	2,00	2,00	2,00	2,00	2,00	2,00	2,00	2,00
2,00	1,00	2,00	2,00	2,00	2,00	2,00	2,00	2,00
2,00	2,00	2,00	2,00	2,00	2,00	2,00	2,00	2,00
2,00	2,00	2,00	2,00	2,00	2,00	2,00	2,00	2,00
1,00	2,00	2,00	2,00	1,00	2,00	2,00	1,00	2,00
2,00	1,00	2,00	2,00	1,00	1,00	2,00	2,00	2,00
1,00	1,00	2,00	2,00	1,00	2,00	2,00	2,00	2,00
2,00	2,00	2,00	2,00	2,00	2,00	2,00	2,00	2,00
2,00	1,00	1,00	2,00	1,00	1,00	2,00	1,00	1,00
2,00	2,00	2,00	2,00	2,00	2,00	2,00	2,00	2,00
2,00	2,00	2,00	2,00	1,00	2,00	2,00	1,00	2,00
2,00	2,00	2,00	2,00	2,00	2,00	2,00	1,00	1,00
2,00	2,00	2,00	2,00	2,00	2,00	2,00	2,00	2,00
2,00	99,00	2,00	2,00	1,00	2,00	2,00	1,00	2,00
2,00	2,00	2,00	1,00	2,00	2,00	2,00	2,00	2,00
2,00	2,00	2,00	2,00	2,00	2,00	2,00	1,00	2,00
2,00	99,00	2,00	2,00	1,00	2,00	2,00	1,00	2,00
2,00	2,00	2,00	1,00	2,00	2,00	1,00	1,00	2,00
2,00	2,00	2,00	2,00	2,00	1,00	2,00	1,00	2,00
2,00	2,00	2,00	2,00	2,00	1,00	2,00	1,00	2,00
2,00	2,00	2,00	2,00	2,00	2,00	2,00	1,00	2,00
2,00	2,00	2,00	2,00	2,00	2,00	2,00	2,00	2,00
2,00	2,00	2,00	1,00	2,00	2,00	2,00	2,00	2,00
2,00	2,00	2,00	2,00	2,00	2,00	2,00	2,00	2,00

2,00	2,00	2,00	2,00	2,00	2,00	2,00	2,00	2,00
2,00	2,00	2,00	2,00	2,00	2,00	2,00	2,00	2,00
2,00	2,00	2,00	2,00	2,00	2,00	2,00	2,00	2,00
2,00	2,00	2,00	2,00	2,00	2,00	2,00	2,00	2,00
2,00	2,00	2,00	2,00	2,00	2,00	2,00	2,00	2,00
2,00	2,00	2,00	2,00	2,00	2,00	2,00	2,00	2,00
2,00	1,00	2,00	2,00	2,00	2,00	2,00	2,00	2,00
2,00	2,00	2,00	2,00	2,00	1,00	2,00	2,00	2,00
2,00	2,00	2,00	2,00	2,00	2,00	2,00	2,00	2,00
2,00	2,00	2,00	2,00	2,00	2,00	2,00	2,00	2,00
2,00	2,00	2,00	2,00	2,00	2,00	2,00	2,00	2,00
2,00	2,00	2,00	2,00	2,00	2,00	2,00	1,00	2,00
2,00	2,00	2,00	2,00	2,00	2,00	2,00	2,00	2,00
2,00	2,00	2,00	1,00	2,00	2,00	2,00	2,00	2,00
2,00	2,00	2,00	1,00	2,00	1,00	2,00	2,00	2,00
2,00	2,00	2,00	2,00	2,00	2,00	2,00	1,00	2,00
2,00	2,00	2,00	2,00	2,00	2,00	2,00	2,00	2,00
2,00	2,00	2,00	2,00	2,00	2,00	2,00	1,00	2,00
2,00	1,00	2,00	2,00	2,00	1,00	2,00	1,00	2,00
2,00	2,00	2,00	2,00	2,00	2,00	2,00	2,00	2,00
2,00	2,00	2,00	2,00	2,00	2,00	2,00	2,00	2,00
2,00	2,00	2,00	2,00	2,00	2,00	2,00	1,00	2,00
2,00	2,00	2,00	2,00	2,00	2,00	2,00	2,00	2,00
2,00	2,00	2,00	2,00	2,00	2,00	2,00	2,00	2,00
2,00	2,00	2,00	2,00	2,00	2,00	2,00	2,00	2,00
2,00	2,00	2,00	2,00	2,00	2,00	2,00	2,00	2,00
1,00	2,00	2,00	2,00	2,00	2,00	2,00	2,00	2,00
2,00	2,00	2,00	2,00	2,00	2,00	1,00	1,00	2,00
2,00	2,00	2,00	2,00	2,00	2,00	2,00	2,00	2,00
2,00	2,00	2,00	2,00	2,00	2,00	1,00	2,00	2,00
2,00	2,00	2,00	2,00	2,00	2,00	2,00	1,00	2,00
2,00	2,00	2,00	2,00	2,00	2,00	2,00	2,00	2,00
1,00	2,00	2,00	2,00	2,00	2,00	2,00	2,00	2,00
2,00	2,00	2,00	2,00	2,00	2,00	2,00	2,00	2,00
2,00	2,00	2,00	2,00	2,00	2,00	2,00	2,00	2,00
2,00	2,00	2,00	2,00	2,00	2,00	2,00	2,00	2,00
2,00	2,00	2,00	2,00	2,00	2,00	2,00	2,00	2,00
2,00	2,00	2,00	2,00	2,00	2,00	2,00	2,00	2,00
2,00	2,00	2,00	2,00	2,00	2,00	2,00	2,00	2,00
2,00	2,00	2,00	2,00	2,00	2,00	2,00	2,00	2,00
2,00	2,00	2,00	2,00	2,00	2,00	2,00	2,00	2,00
2,00	2,00	2,00	2,00	2,00	2,00	2,00	2,00	2,00
2,00	2,00	2,00	2,00	2,00	2,00	2,00	2,00	2,00
2,00	1,00	1,00	1,00	1,00	1,00	1,00	1,00	1,00
2,00	2,00	2,00	2,00	2,00	1,00	2,00	2,00	2,00
2,00	2,00	2,00	2,00	2,00	2,00	2,00	2,00	2,00
2,00	2,00	2,00	2,00	2,00	2,00	2,00	2,00	2,00
2,00	1,00	2,00	2,00	2,00	2,00	2,00	2,00	2,00
2,00	1,00	1,00	1,00	1,00	1,00	1,00	1,00	1,00
2,00	2,00	2,00	2,00	2,00	2,00	2,00	2,00	2,00

2,00	2,00	2,00	2,00	2,00	2,00	2,00	2,00	2,00
2,00	2,00	2,00	2,00	2,00	2,00	2,00	1,00	2,00
1,00	2,00	2,00	1,00	2,00	2,00	2,00	2,00	2,00
2,00	2,00	2,00	2,00	2,00	2,00	2,00	1,00	2,00
2,00	2,00	2,00	2,00	2,00	2,00	2,00	2,00	2,00
2,00	2,00	2,00	2,00	2,00	2,00	2,00	2,00	2,00
2,00	2,00	2,00	2,00	2,00	2,00	2,00	2,00	2,00
2,00	2,00	2,00	2,00	2,00	2,00	2,00	2,00	2,00
2,00	2,00	2,00	2,00	2,00	2,00	2,00	2,00	2,00
2,00	2,00	2,00	2,00	2,00	2,00	2,00	2,00	2,00
2,00	2,00	2,00	2,00	1,00	2,00	2,00	1,00	2,00
2,00	2,00	2,00	2,00	2,00	2,00	2,00	1,00	2,00
2,00	2,00	2,00	2,00	2,00	2,00	2,00	2,00	2,00
2,00	2,00	2,00	2,00	2,00	2,00	2,00	2,00	2,00
2,00	2,00	2,00	2,00	2,00	2,00	2,00	2,00	2,00
2,00	2,00	2,00	2,00	2,00	2,00	2,00	2,00	2,00
2,00	2,00	2,00	2,00	1,00	2,00	2,00	2,00	2,00
2,00	2,00	1,00	2,00	2,00	2,00	1,00	2,00	2,00
2,00	2,00	2,00	2,00	2,00	1,00	2,00	2,00	2,00
2,00	2,00	2,00	2,00	2,00	2,00	2,00	2,00	2,00
2,00	2,00	2,00	2,00	2,00	2,00	2,00	2,00	2,00
2,00	2,00	2,00	2,00	2,00	2,00	2,00	1,00	2,00
2,00	2,00	2,00	2,00	2,00	2,00	2,00	2,00	2,00
2,00	1,00	2,00	1,00	2,00	2,00	2,00	2,00	2,00
2,00	2,00	2,00	2,00	2,00	2,00	2,00	2,00	2,00
2,00	2,00	2,00	1,00	2,00	2,00	2,00	2,00	2,00
2,00	2,00	2,00	2,00	2,00	2,00	2,00	2,00	2,00
2,00	1,00	2,00	1,00	2,00	2,00	2,00	1,00	2,00
1,00	1,00	1,00	1,00	1,00	2,00	2,00	1,00	2,00
2,00	2,00	2,00	2,00	2,00	2,00	2,00	2,00	2,00
2,00	2,00	2,00	2,00	2,00	2,00	2,00	2,00	2,00
1,00	2,00	2,00	1,00	2,00	1,00	2,00	2,00	2,00
2,00	2,00	2,00	2,00	2,00	2,00	2,00	2,00	2,00
2,00	2,00	2,00	2,00	2,00	1,00	1,00	1,00	2,00
1,00	2,00	1,00	2,00	2,00	2,00	2,00	2,00	2,00
2,00	2,00	2,00	1,00	1,00	2,00	2,00	2,00	2,00
2,00	1,00	2,00	2,00	1,00	1,00	1,00	1,00	2,00
2,00	2,00	2,00	2,00	2,00	2,00	2,00	2,00	2,00
2,00	2,00	2,00	2,00	2,00	2,00	2,00	2,00	2,00
2,00	2,00	2,00	2,00	2,00	2,00	2,00	2,00	2,00
2,00	2,00	2,00	2,00	2,00	2,00	2,00	2,00	2,00
2,00	2,00	2,00	2,00	2,00	2,00	2,00	2,00	2,00
2,00	2,00	2,00	2,00	2,00	2,00	2,00	1,00	2,00
2,00	2,00	2,00	2,00	2,00	2,00	2,00	1,00	2,00
2,00	2,00	2,00	2,00	2,00	2,00	2,00	2,00	2,00
2,00	2,00	2,00	2,00	2,00	2,00	2,00	2,00	2,00
2,00	1,00	2,00	2,00	1,00	1,00	1,00	1,00	2,00
2,00	2,00	2,00	2,00	2,00	2,00	2,00	1,00	2,00
2,00	2,00	2,00	2,00	2,00	2,00	2,00	2,00	2,00
2,00	2,00	2,00	2,00	2,00	2,00	2,00	2,00	2,00

2,00	2,00	2,00	2,00	2,00	2,00	2,00	2,00	2,00
2,00	2,00	2,00	1,00	1,00	2,00	2,00	2,00	2,00
2,00	2,00	2,00	2,00	2,00	2,00	2,00	2,00	2,00
2,00	2,00	2,00	2,00	2,00	2,00	2,00	2,00	2,00
2,00	2,00	2,00	2,00	2,00	2,00	2,00	2,00	2,00
2,00	2,00	2,00	1,00	1,00	2,00	2,00	1,00	2,00
2,00	2,00	2,00	1,00	2,00	2,00	1,00	2,00	2,00
2,00	2,00	2,00	2,00	2,00	2,00	2,00	2,00	2,00
2,00	2,00	2,00	2,00	2,00	2,00	2,00	2,00	2,00
2,00	2,00	2,00	2,00	2,00	2,00	2,00	2,00	2,00
2,00	2,00	2,00	2,00	2,00	2,00	2,00	2,00	2,00
2,00	2,00	2,00	2,00	2,00	2,00	1,00	2,00	2,00
2,00	2,00	2,00	2,00	2,00	2,00	2,00	1,00	2,00
2,00	2,00	2,00	2,00	2,00	2,00	2,00	2,00	2,00
2,00	2,00	2,00	2,00	2,00	2,00	2,00	2,00	2,00
2,00	2,00	2,00	2,00	2,00	2,00	2,00	2,00	2,00
2,00	2,00	2,00	2,00	2,00	2,00	2,00	2,00	2,00
2,00	2,00	1,00	2,00	2,00	2,00	1,00	1,00	1,00
1,00	2,00	2,00	2,00	2,00	2,00	2,00	1,00	2,00
2,00	2,00	2,00	2,00	2,00	2,00	2,00	2,00	2,00
2,00	2,00	2,00	2,00	2,00	2,00	2,00	2,00	2,00
2,00	2,00	2,00	2,00	2,00	2,00	2,00	2,00	2,00
1,00	2,00	2,00	1,00	2,00	1,00	2,00	1,00	1,00
2,00	2,00	1,00	2,00	2,00	2,00	2,00	2,00	2,00
2,00	2,00	2,00	2,00	2,00	2,00	2,00	1,00	2,00
2,00	2,00	2,00	2,00	2,00	2,00	2,00	2,00	2,00
2,00	2,00	2,00	2,00	2,00	2,00	2,00	2,00	2,00
2,00	2,00	2,00	2,00	2,00	2,00	2,00	2,00	2,00
2,00	2,00	2,00	2,00	2,00	2,00	2,00	2,00	2,00
2,00	2,00	2,00	2,00	2,00	2,00	2,00	1,00	1,00
2,00	2,00	2,00	2,00	2,00	2,00	2,00	2,00	2,00
2,00	2,00	2,00	2,00	1,00	2,00	2,00	2,00	2,00
2,00	2,00	2,00	2,00	2,00	2,00	2,00	1,00	2,00
2,00	1,00	2,00	2,00	1,00	2,00	2,00	1,00	2,00
2,00	2,00	2,00	2,00	2,00	2,00	2,00	2,00	2,00
2,00	2,00	2,00	2,00	2,00	2,00	2,00	1,00	2,00
2,00	2,00	2,00	2,00	2,00	2,00	2,00	2,00	2,00
2,00	2,00	2,00	2,00	2,00	2,00	2,00	2,00	2,00
2,00	2,00	1,00	2,00	2,00	1,00	1,00	2,00	2,00
2,00	2,00	2,00	2,00	2,00	2,00	2,00	2,00	2,00
2,00	2,00	2,00	2,00	2,00	2,00	2,00	2,00	2,00
2,00	2,00	2,00	2,00	2,00	2,00	2,00	2,00	1,00
2,00	2,00	2,00	2,00	2,00	2,00	2,00	2,00	2,00
2,00	2,00	2,00	2,00	1,00	2,00	2,00	2,00	2,00
2,00	2,00	2,00	2,00	1,00	1,00	2,00	1,00	2,00
2,00	2,00	2,00	2,00	2,00	2,00	2,00	2,00	2,00
2,00	2,00	2,00	2,00	1,00	1,00	2,00	2,00	2,00
2,00	2,00	2,00	2,00	2,00	2,00	2,00	2,00	2,00
2,00	2,00	2,00	2,00	2,00	2,00	2,00	2,00	2,00
2,00	2,00	2,00	2,00	2,00	2,00	2,00	2,00	2,00

2,00	2,00	2,00	2,00	2,00	2,00	2,00	2,00	2,00
2,00	2,00	2,00	2,00	2,00	1,00	2,00	1,00	2,00
2,00	2,00	2,00	2,00	2,00	2,00	2,00	1,00	2,00
2,00	2,00	2,00	2,00	2,00	2,00	2,00	2,00	2,00
2,00	2,00	2,00	2,00	2,00	2,00	2,00	2,00	2,00
2,00	2,00	2,00	2,00	2,00	2,00	2,00	2,00	2,00
2,00	2,00	2,00	2,00	2,00	2,00	2,00	2,00	2,00
2,00	2,00	2,00	2,00	2,00	2,00	2,00	2,00	2,00
2,00	2,00	2,00	2,00	2,00	2,00	2,00	2,00	2,00
2,00	2,00	2,00	2,00	2,00	2,00	2,00	2,00	2,00
2,00	2,00	2,00	2,00	2,00	2,00	2,00	2,00	2,00
2,00	2,00	2,00	2,00	2,00	2,00	2,00	2,00	2,00
2,00	2,00	2,00	2,00	2,00	2,00	2,00	2,00	2,00
2,00	2,00	2,00	2,00	2,00	2,00	2,00	2,00	2,00
2,00	2,00	2,00	2,00	2,00	2,00	2,00	2,00	2,00
2,00	2,00	2,00	2,00	2,00	2,00	2,00	2,00	2,00
2,00	2,00	2,00	2,00	2,00	2,00	2,00	2,00	2,00
2,00	2,00	2,00	2,00	2,00	2,00	2,00	1,00	2,00
2,00	2,00	2,00	2,00	2,00	2,00	2,00	2,00	2,00
2,00	2,00	2,00	2,00	2,00	2,00	2,00	2,00	2,00
2,00	2,00	2,00	2,00	2,00	2,00	2,00	2,00	2,00
2,00	2,00	2,00	2,00	2,00	2,00	2,00	2,00	2,00
2,00	2,00	2,00	2,00	2,00	2,00	2,00	2,00	2,00
2,00	2,00	2,00	2,00	2,00	2,00	2,00	2,00	2,00
2,00	2,00	2,00	2,00	2,00	1,00	2,00	2,00	2,00
2,00	2,00	2,00	2,00	2,00	2,00	2,00	2,00	2,00
2,00	2,00	2,00	2,00	2,00	2,00	2,00	2,00	2,00
2,00	2,00	2,00	1,00	2,00	2,00	2,00	1,00	2,00
2,00	2,00	2,00	2,00	2,00	2,00	2,00	1,00	2,00
2,00	2,00	2,00	2,00	2,00	2,00	2,00	2,00	2,00
2,00	2,00	2,00	2,00	2,00	2,00	2,00	2,00	2,00
2,00	2,00	2,00	2,00	2,00	2,00	2,00	2,00	2,00
2,00	2,00	2,00	2,00	2,00	2,00	2,00	2,00	2,00
2,00	2,00	2,00	2,00	1,00	2,00	2,00	2,00	2,00
2,00	2,00	2,00	2,00	2,00	2,00	2,00	2,00	2,00
2,00	2,00	2,00	2,00	2,00	2,00	2,00	2,00	2,00
2,00	2,00	2,00	2,00	2,00	2,00	2,00	2,00	2,00
2,00	2,00	2,00	2,00	2,00	2,00	2,00	2,00	2,00
2,00	2,00	2,00	2,00	1,00	1,00	2,00	1,00	1,00
2,00	2,00	2,00	2,00	2,00	2,00	2,00	2,00	2,00
2,00	2,00	2,00	1,00	2,00	2,00	2,00	2,00	2,00
1,00	2,00	2,00	2,00	2,00	2,00	2,00	2,00	2,00
2,00	2,00	2,00	2,00	2,00	2,00	2,00	2,00	2,00
2,00	2,00	2,00	2,00	2,00	2,00	2,00	2,00	2,00
2,00	2,00	2,00	2,00	2,00	2,00	2,00	2,00	2,00
2,00	2,00	2,00	2,00	2,00	2,00	2,00	2,00	2,00
2,00	2,00	2,00	2,00	2,00	2,00	2,00	2,00	2,00
2,00	2,00	2,00	2,00	2,00	2,00	2,00	1,00	2,00
2,00	2,00	2,00	2,00	2,00	2,00	2,00	2,00	2,00
2,00	2,00	2,00	2,00	2,00	2,00	1,00	2,00	2,00

2,00	2,00	2,00	2,00	2,00	2,00	2,00	2,00	2,00
2,00	2,00	2,00	2,00	2,00	2,00	2,00	2,00	2,00
2,00	2,00	2,00	2,00	2,00	2,00	2,00	2,00	2,00
2,00	2,00	2,00	2,00	2,00	2,00	2,00	2,00	2,00
2,00	2,00	2,00	2,00	2,00	2,00	2,00	2,00	2,00
2,00	2,00	2,00	2,00	2,00	2,00	2,00	2,00	2,00
2,00	2,00	2,00	2,00	2,00	2,00	2,00	2,00	2,00
2,00	2,00	2,00	2,00	2,00	1,00	1,00	2,00	2,00
2,00	2,00	2,00	2,00	2,00	1,00	1,00	2,00	2,00
2,00	2,00	2,00	2,00	2,00	2,00	2,00	2,00	2,00
2,00	2,00	2,00	2,00	2,00	2,00	2,00	2,00	2,00
2,00	2,00	2,00	2,00	2,00	2,00	2,00	2,00	2,00
2,00	2,00	2,00	1,00	2,00	2,00	2,00	2,00	2,00
2,00	2,00	2,00	2,00	2,00	2,00	2,00	2,00	2,00
2,00	2,00	2,00	2,00	2,00	2,00	2,00	2,00	2,00
2,00	1,00	2,00	2,00	2,00	2,00	2,00	2,00	2,00
2,00	2,00	2,00	2,00	2,00	2,00	2,00	1,00	2,00
2,00	2,00	2,00	2,00	2,00	2,00	2,00	2,00	2,00
2,00	2,00	2,00	2,00	2,00	2,00	2,00	2,00	2,00
2,00	2,00	2,00	2,00	2,00	2,00	2,00	2,00	2,00
2,00	2,00	2,00	2,00	2,00	2,00	2,00	1,00	2,00
2,00	1,00	2,00	2,00	2,00	2,00	2,00	2,00	2,00
2,00	2,00	2,00	1,00	1,00	2,00	2,00	2,00	2,00
2,00	2,00	2,00	2,00	2,00	2,00	2,00	2,00	2,00
2,00	2,00	2,00	2,00	2,00	2,00	2,00	2,00	2,00
2,00	2,00	2,00	2,00	2,00	2,00	2,00	2,00	2,00
2,00	2,00	2,00	2,00	2,00	2,00	2,00	2,00	2,00
1,00	2,00	2,00	2,00	2,00	2,00	2,00	2,00	2,00
2,00	2,00	2,00	2,00	2,00	2,00	2,00	2,00	2,00
2,00	2,00	2,00	2,00	2,00	2,00	2,00	2,00	2,00
2,00	2,00	2,00	2,00	2,00	2,00	2,00	2,00	2,00
2,00	2,00	2,00	2,00	2,00	2,00	2,00	2,00	2,00
2,00	2,00	2,00	2,00	2,00	2,00	2,00	2,00	2,00
2,00	2,00	2,00	2,00	2,00	2,00	2,00	2,00	2,00
2,00	2,00	2,00	2,00	2,00	2,00	2,00	2,00	2,00
2,00	1,00	2,00	2,00	2,00	2,00	2,00	2,00	2,00
2,00	2,00	2,00	2,00	2,00	2,00	2,00	2,00	2,00
2,00	2,00	2,00	2,00	2,00	2,00	2,00	2,00	2,00
2,00	2,00	2,00	2,00	2,00	2,00	2,00	2,00	2,00
1,00	2,00	2,00	2,00	1,00	1,00	2,00	1,00	2,00
1,00	2,00	2,00	2,00	2,00	2,00	2,00	2,00	2,00
2,00	2,00	2,00	2,00	2,00	2,00	2,00	2,00	2,00
2,00	2,00	2,00	2,00	2,00	2,00	2,00	2,00	2,00
2,00	2,00	2,00	1,00	2,00	2,00	2,00	2,00	2,00
2,00	2,00	2,00	2,00	2,00	2,00	2,00	2,00	2,00
2,00	2,00	2,00	2,00	2,00	2,00	2,00	1,00	2,00
2,00	2,00	2,00	2,00	2,00	2,00	2,00	2,00	2,00
2,00	1,00	2,00	2,00	2,00	2,00	2,00	2,00	2,00
2,00	2,00	2,00	2,00	2,00	2,00	2,00	2,00	2,00
2,00	2,00	2,00	2,00	2,00	2,00	2,00	2,00	2,00

2,00	2,00	2,00	2,00	2,00	2,00	2,00	2,00	2,00
2,00	1,00	2,00	2,00	2,00	2,00	2,00	2,00	2,00
2,00	2,00	2,00	2,00	2,00	2,00	2,00	1,00	2,00
2,00	2,00	2,00	2,00	2,00	2,00	2,00	2,00	2,00
2,00	2,00	2,00	2,00	2,00	2,00	2,00	2,00	2,00
2,00	1,00	2,00	2,00	2,00	2,00	2,00	2,00	2,00
2,00	2,00	2,00	2,00	2,00	1,00	2,00	2,00	2,00
2,00	2,00	2,00	2,00	2,00	2,00	2,00	2,00	2,00
2,00	2,00	2,00	2,00	2,00	2,00	2,00	2,00	2,00
2,00	2,00	2,00	2,00	2,00	2,00	2,00	2,00	2,00
1,00	1,00	1,00	1,00	2,00	2,00	1,00	1,00	1,00
2,00	2,00	2,00	2,00	2,00	2,00	2,00	2,00	2,00
2,00	2,00	2,00	2,00	2,00	2,00	2,00	2,00	2,00
2,00	2,00	2,00	2,00	2,00	2,00	2,00	2,00	2,00
2,00	2,00	2,00	2,00	2,00	2,00	2,00	2,00	2,00
2,00	2,00	2,00	2,00	2,00	2,00	2,00	2,00	2,00
2,00	2,00	2,00	2,00	2,00	2,00	2,00	2,00	2,00
2,00	2,00	2,00	2,00	2,00	2,00	2,00	2,00	2,00
2,00	2,00	2,00	2,00	2,00	2,00	2,00	2,00	2,00
2,00	2,00	2,00	2,00	2,00	2,00	2,00	2,00	2,00
2,00	2,00	2,00	2,00	2,00	2,00	1,00	2,00	2,00
2,00	2,00	2,00	2,00	2,00	2,00	2,00	1,00	2,00
2,00	2,00	2,00	2,00	2,00	2,00	2,00	2,00	2,00
2,00	2,00	2,00	1,00	2,00	2,00	2,00	2,00	2,00
2,00	2,00	2,00	2,00	2,00	2,00	2,00	1,00	2,00
2,00	2,00	2,00	2,00	2,00	2,00	2,00	2,00	2,00
2,00	1,00	2,00	2,00	2,00	2,00	2,00	1,00	2,00
2,00	2,00	2,00	2,00	2,00	2,00	2,00	2,00	2,00
2,00	2,00	2,00	2,00	2,00	2,00	2,00	2,00	2,00
2,00	2,00	2,00	2,00	2,00	2,00	2,00	2,00	2,00
2,00	2,00	2,00	1,00	2,00	2,00	2,00	2,00	2,00
2,00	2,00	2,00	2,00	2,00	2,00	2,00	2,00	2,00

Slappedyou	Pushedyou	Hityou	Kickedyou	Chokedorb	Threatened	TOT_physic	Whodidthi	Howoldfro
0	0	0	0	1	0	1	99,00	4,00
0	0	0	0	0	0	0	99,00	99,00
0	0	0	0	0	0	0	1000,00	1000,00
0	0	0	0	0	0	0	99,00	99,00
1	1	1	0	1	0	4	2,00	1,00
0	0	0	0	0	0	0	1000,00	1000,00
0	0	0	0	0	0	0	1000,00	1000,00
0	0	0	0	0	0	0	1000,00	1000,00
0	0	0	0	0	0	0	1000,00	1000,00
0	1	0	0	0	0	1	1,00	1,00
0	0	0	0	0	0	0	1000,00	1000,00
0	0	0	0	0	0	0	1000,00	1000,00
1	1	1	1	0	0	4	2,00	1,00
1	1	0	0	0	0	2	2,00	1,00
1	1	0	1	1	0	4	2,00	1,00
0	0	0	0	0	0	0	1000,00	1000,00
0	0	0	0	0	0	0	1000,00	1000,00
0	0	0	0	0	0	0	1000,00	1000,00
0	0	0	0	1	0	1	1,00	3,00
1	1	0	0	0	0	2	2,00	1,00
1	0	0	0	0	0	1	1,00	1,00
0	1	0	0	0	0	1	1,00	1,00
0	0	0	0	0	0	0	1000,00	1000,00
0	0	0	0	0	0	0	1000,00	1000,00
0	0	0	0	0	0	0	1000,00	1000,00
0	0	99	0	0	0	#NULL!	1000,00	1000,00
0	0	0	0	0	0	0	1000,00	1000,00
0	0	0	0	0	0	0	1000,00	1000,00
0	0	0	0	0	#NULL!	#NULL!	1000,00	1000,00
0	0	0	0	0	0	0	1000,00	1000,00
0	0	0	0	0	0	0	1000,00	1000,00
0	0	0	0	0	0	0	1000,00	1000,00
1	1	1	1	99	99	#NULL!	2,00	1,00
1	1	1	1	0	0	4	2,00	1,00
0	0	0	0	0	0	0	1000,00	1000,00
1	1	1	1	0	0	4	2,00	1,00
1	1	0	0	0	0	2	1,00	1,00
99	99	99	99	99	99	#NULL!	1000,00	1000,00
0	0	0	0	0	0	0	1000,00	1000,00
1	0	0	1	0	0	2	1,00	2,00
0	0	0	0	0	0	0	1000,00	1000,00
0	0	0	0	0	0	0	1000,00	1000,00
0	1	0	0	0	0	1	1,00	2,00
0	0	0	0	0	0	0	1000,00	1000,00
0	0	0	0	0	0	0	1000,00	1000,00
0	0	0	0	0	0	0	1000,00	1000,00
1	1	1	0	0	0	3	1,00	1,00
0	0	0	0	0	0	0	1000,00	1000,00
0	0	0	0	0	0	0	1000,00	1000,00

0	1	0	0	0	0	1	2,00	3,00
0	0	0	0	0	0	0	1000,00	1000,00
0	0	0	0	0	0	0	1000,00	1000,00
0	0	0	0	0	0	0	1000,00	1000,00
0	0	0	0	0	0	0	1000,00	1000,00
1	0	0	0	0	0	1	2,00	99,00
1	1	1	0	0	0	3	2,00	1,00
0	0	0	0	0	0	0	1000,00	1000,00
1	1	1	1	0	0	4	1,00	2,00
0	0	0	0	0	0	0	1000,00	1000,00
0	0	0	0	0	0	0	1000,00	1000,00
0	0	0	0	0	0	0	1000,00	1000,00
0	0	0	0	0	0	0	1000,00	1000,00
0	1	1	1	0	0	3	2,00	1,00
0	0	0	0	0	0	0	1000,00	1000,00
1	0	0	0	0	0	1	2,00	1,00
0	1	0	0	0	0	1	2,00	1,00
0	0	0	0	0	0	0	1000,00	1000,00
0	0	0	0	0	0	0	1000,00	1000,00
0	1	0	0	0	0	1	2,00	1,00
0	0	0	0	0	0	0	1000,00	1000,00
0	1	1	0	0	0	2	1,00	3,00
0	0	0	0	0	0	0	1000,00	1000,00
0	0	0	0	1	0	1	2,00	1,00
0	0	0	0	0	0	0	1000,00	1000,00
0	0	0	0	0	0	0	1000,00	1000,00
0	1	0	0	0	0	1	2,00	1,00
0	0	0	0	0	0	0	1000,00	1000,00
1	0	0	0	0	0	1	1,00	1,00
1	1	0	0	0	0	2	1,00	1,00
0	0	0	0	0	0	0	1000,00	1000,00
0	0	0	0	0	0	0	1000,00	1000,00
0	0	0	0	0	0	0	1000,00	1000,00
1	1	1	0	0	0	3	2,00	3,00
0	1	0	0	0	0	1	2,00	1,00
0	0	0	0	0	0	0	1000,00	1000,00
0	1	0	1	0	0	2	3,00	1,00
0	0	0	0	0	0	0	1000,00	1000,00
0	0	0	0	0	0	0	1000,00	1000,00
0	1	0	0	0	0	1	3,00	1,00
0	0	0	0	0	0	0	1000,00	1000,00
0	0	0	0	0	0	0	1000,00	1000,00
0	0	0	0	0	0	0	1000,00	1000,00
0	0	0	0	0	0	0	1000,00	1000,00
0	0	0	0	0	0	0	1000,00	1000,00
1	1	0	0	0	0	2	2,00	1,00
0	0	0	0	0	0	0	1000,00	1000,00
0	0	1	0	0	0	1	1,00	1,00
0	0	0	0	0	0	0	1000,00	1000,00
0	1	0	0	0	0	1	1,00	4,00

0	0	0	0	0	0	0	1000,00	1000,00
0	0	0	0	0	0	0	1000,00	1000,00
0	0	0	0	0	0	0	1000,00	1000,00
0	0	0	0	0	0	0	1000,00	1000,00
0	0	0	0	0	0	0	1000,00	1000,00
0	0	0	0	0	0	0	1000,00	1000,00
0	0	0	0	0	0	0	1000,00	1000,00
0	0	0	0	0	0	0	1000,00	1000,00
0	0	0	0	0	0	0	1000,00	1000,00
0	0	0	0	0	0	0	1000,00	1000,00
0	0	0	0	0	0	0	1000,00	1000,00
0	0	0	0	0	0	0	1000,00	1000,00
0	0	0	0	0	0	0	1000,00	1000,00
1	1	0	1	0	0	3	1,00	2,00
0	0	0	0	0	0	0	1000,00	1000,00
0	0	0	0	0	0	0	1000,00	1000,00
0	0	0	0	0	0	0	1000,00	1000,00
0	0	0	0	0	0	0	1000,00	1000,00
0	0	0	0	0	0	0	1000,00	1000,00
0	0	0	0	0	0	0	1000,00	1000,00
0	0	0	0	0	0	0	1000,00	1000,00
0	0	0	0	0	0	0	1000,00	1000,00
0	0	0	0	0	0	0	1000,00	1000,00
0	0	0	0	0	0	0	1000,00	1000,00
1	1	0	1	0	0	3	2,00	1,00
0	0	0	0	0	0	0	1000,00	1000,00
1	0	0	0	0	0	1	1,00	1,00
1	0	0	0	0	0	1	2,00	1,00
0	0	0	0	0	0	0	1000,00	1000,00
0	0	0	0	0	0	0	1000,00	1000,00
0	0	0	0	0	0	0	1000,00	1000,00
0	1	0	0	0	0	1	1,00	1,00
0	0	0	0	0	0	0	1000,00	1000,00
0	0	0	0	0	0	0	1000,00	1000,00
0	0	0	0	0	0	0	1000,00	1000,00
0	0	0	0	0	0	0	1000,00	1000,00
0	1	0	0	0	0	1	1000,00	1000,00
0	0	0	0	0	0	0	1000,00	1000,00
0	0	0	0	0	0	0	1000,00	1000,00
0	0	0	0	0	0	0	1000,00	1000,00
0	0	0	0	0	0	0	1000,00	1000,00
0	0	0	0	0	0	0	1000,00	1000,00
0	0	0	0	0	0	0	1000,00	1000,00
0	0	0	0	0	0	0	1000,00	1000,00
0	0	0	0	0	0	0	1000,00	1000,00
0	1	0	0	0	0	1	1000,00	1000,00
0	0	0	0	0	0	0	1000,00	1000,00
0	0	0	0	0	0	0	1000,00	1000,00
0	0	1	0	0	0	1	2,00	1,00
0	0	0	0	0	0	0	1000,00	1000,00

0	0	0	0	0	0	0	1000,00	1000,00
0	0	0	0	0	0	0	1000,00	1000,00
0	1	0	0	0	0	1	1,00	1,00
0	0	0	0	0	0	0	1000,00	1000,00
0	0	0	0	0	0	0	1000,00	1000,00
0	0	0	0	0	0	0	1000,00	1000,00
0	0	0	0	0	0	0	1000,00	1000,00
0	0	0	0	0	0	0	1000,00	1000,00
0	0	0	0	0	0	0	1000,00	1000,00
0	0	0	0	0	0	0	1000,00	1000,00
1	0	0	0	0	0	1	2,00	3,00
0	0	0	0	0	0	0	1000,00	1000,00
0	0	0	0	0	0	0	1000,00	1000,00
0	0	0	0	0	0	0	1000,00	1000,00
0	0	0	0	0	0	0	1000,00	1000,00
0	0	0	0	0	0	0	1000,00	1000,00
0	0	0	0	0	0	0	1000,00	1000,00
0	0	0	0	0	0	0	1000,00	1000,00
0	0	0	0	0	0	0	1000,00	1000,00
0	0	0	0	0	0	0	1000,00	1000,00
0	0	0	0	0	0	0	1000,00	1000,00
0	0	0	0	0	0	0	1000,00	1000,00
1	0	0	0	0	0	1	2,00	1,00
1	0	0	0	0	0	1	2,00	4,00
0	0	0	0	0	0	0	1000,00	1000,00
0	0	0	0	0	0	0	1000,00	1000,00
0	0	0	0	0	0	0	1000,00	1000,00
0	0	0	0	0	0	0	1000,00	1000,00
0	1	0	0	0	0	1	1,00	1,00
0	0	0	0	0	0	0	1000,00	1000,00
0	0	0	0	0	0	0	1000,00	1000,00
0	0	0	0	0	0	0	1000,00	1000,00
0	0	0	0	0	0	0	1000,00	1000,00
1	1	1	0	0	0	3	2,00	3,00
0	0	0	0	0	0	0	1000,00	1000,00
0	0	0	0	0	0	0	1000,00	1000,00
0	0	0	0	1	0	1	1000,00	1000,00
0	0	0	0	0	0	0	1000,00	1000,00
0	0	0	0	0	0	0	1000,00	1000,00
0	0	0	0	0	0	0	1000,00	1000,00
0	0	0	0	0	0	0	1000,00	1000,00
0	0	0	0	0	0	0	1000,00	1000,00
0	0	0	0	0	0	0	1000,00	1000,00
0	0	0	0	0	0	0	1000,00	1000,00
1	0	0	0	0	0	1	3,00	1,00
0	0	0	0	0	0	0	1000,00	1000,00
0	0	0	0	0	0	0	1000,00	1000,00
0	0	0	0	0	0	0	1000,00	1000,00
0	0	0	0	0	0	0	1000,00	1000,00
0	0	0	0	0	0	0	1000,00	1000,00

0	0	0	0	0	0	0	1000,00	1000,00
0	0	0	0	0	0	0	1000,00	1000,00
0	0	0	0	0	0	0	1000,00	1000,00
0	0	0	0	0	0	0	1000,00	1000,00
0	0	0	0	0	0	0	1000,00	1000,00
0	0	0	0	0	0	0	1000,00	1000,00
0	0	0	0	0	0	0	1000,00	1000,00
1	0	0	0	0	0	1	1,00	1,00
0	0	0	0	0	0	0	1000,00	1000,00
0	0	0	0	0	0	0	1000,00	1000,00
0	0	0	0	0	0	0	1000,00	1000,00
0	0	0	0	0	0	0	1000,00	1000,00
0	0	0	0	0	0	0	1000,00	1000,00
0	0	0	0	0	0	0	1000,00	1000,00
0	0	0	0	0	0	0	1000,00	1000,00
0	0	0	0	0	0	0	1000,00	1000,00
0	0	0	0	0	0	0	1000,00	1000,00
0	1	0	1	0	0	2	1,00	1,00
0	0	0	0	0	0	0	1000,00	1000,00
0	0	0	0	0	0	0	1000,00	1000,00
0	0	0	0	0	0	0	1000,00	1000,00
0	0	0	0	0	0	0	1000,00	1000,00
0	0	0	0	0	0	0	1000,00	1000,00
0	1	0	0	0	0	1	1,00	2,00
0	0	0	0	0	0	0	1000,00	1000,00
0	0	0	0	0	0	0	1000,00	1000,00
0	0	0	0	0	0	0	1000,00	1000,00
1	1	1	0	0	0	3	2,00	1,00
1	1	0	0	0	0	2	1,00	3,00
0	0	0	0	0	0	0	1000,00	1000,00
0	0	0	0	0	0	0	1000,00	1000,00
0	0	0	0	0	0	0	1000,00	1000,00
0	0	0	0	0	0	0	1000,00	1000,00
0	1	0	0	0	0	1	3,00	3,00
1	1	1	0	0	0	3	2,00	2,00
0	0	0	0	0	0	0	2,00	1,00
0	0	0	0	0	0	0	1000,00	1000,00
0	0	0	0	0	0	0	1000,00	1000,00
0	1	0	0	0	0	1	2,00	1,00
0	0	0	0	0	0	0	1000,00	1000,00
1	0	1	0	0	0	2	2,00	1,00
1	0	0	0	0	0	1	1,00	1,00
0	0	0	0	0	0	0	1000,00	1000,00
0	0	0	0	0	0	0	1000,00	1000,00
0	0	0	0	0	0	0	1000,00	1000,00
0	0	0	0	0	0	0	1000,00	1000,00
0	0	0	0	0	0	0	1000,00	1000,00
0	0	0	0	0	0	0	1000,00	1000,00
0	0	0	0	0	0	0	1000,00	1000,00
0	0	0	0	0	0	0	1000,00	1000,00

0	0	0	0	0	0	0	1000,00	1000,00
0	0	0	0	0	0	0	1000,00	1000,00
0	0	0	0	0	0	0	1000,00	1000,00
0	0	0	0	0	0	0	1000,00	1000,00
0	0	0	0	0	0	0	1000,00	1000,00
0	0	0	0	0	0	0	1000,00	1000,00
0	0	0	0	0	0	0	1000,00	1000,00
0	0	0	0	0	0	0	1000,00	1000,00
0	0	0	0	0	0	0	1000,00	1000,00
0	0	0	0	0	0	0	1000,00	1000,00
1	0	0	0	0	0	1	3,00	1,00
0	0	0	0	0	0	0	1000,00	1000,00
0	0	0	0	0	0	0	1000,00	1000,00
1	1	0	0	0	0	2	1,00	1,00
0	0	0	0	0	0	0	1000,00	1000,00
0	0	0	0	0	0	0	1000,00	1000,00
0	0	0	0	0	0	0	1000,00	1000,00
0	0	0	0	0	0	0	1000,00	1000,00
0	0	0	0	0	0	0	1000,00	1000,00
0	0	0	0	0	0	0	1000,00	1000,00
0	0	0	0	0	0	0	1000,00	1000,00
0	0	0	0	0	0	0	1000,00	1000,00
1	0	1	1	0	0	3	2,00	1,00
0	0	0	0	0	0	0	1000,00	1000,00
0	0	0	0	0	0	0	1000,00	1000,00
0	0	0	0	0	0	0	1000,00	1000,00
0	0	0	0	0	0	0	1000,00	1000,00
0	0	0	0	0	0	0	1000,00	1000,00
0	0	0	0	0	0	0	1000,00	1000,00
0	1	0	0	0	0	1	2,00	3,00
0	0	0	0	0	0	0	1000,00	1000,00
0	0	0	0	0	0	0	1000,00	1000,00
0	0	0	0	0	0	0	1000,00	1000,00
0	0	0	0	0	0	0	1000,00	1000,00
0	0	0	0	0	0	0	1000,00	1000,00
0	1	0	0	0	0	1	2,00	1,00
0	0	0	0	0	0	0	1000,00	1000,00
0	0	0	0	0	0	0	1000,00	1000,00
0	1	1	0	0	0	2	1,00	4,00
0	0	0	0	0	0	0	1000,00	1000,00
0	0	0	0	0	0	0	1000,00	1000,00
0	0	0	0	0	0	0	1000,00	1000,00
0	0	0	0	0	0	0	1000,00	1000,00
0	0	0	0	0	0	0	1000,00	1000,00
0	0	0	0	0	0	0	1000,00	1000,00
0	0	0	0	0	0	0	1000,00	1000,00
1	1	0	0	0	0	2	1,00	1,00
0	0	0	0	0	0	0	1000,00	1000,00
0	0	0	0	0	0	0	1000,00	1000,00
0	0	0	0	0	0	0	1000,00	1000,00

0	0	0	0	0	0	0	1000,00	1000,00
0	0	0	0	0	0	0	1000,00	1000,00
1	1	0	0	0	0	2	2,00	1,00
0	1	1	#NULL!	0	0	#NULL!	2,00	4,00
0	0	0	0	0	0	0	1000,00	1000,00
0	0	0	0	0	0	0	1000,00	1000,00
0	0	0	0	0	0	0	1000,00	1000,00
0	1	0	0	0	0	1	2,00	1,00
0	0	0	0	0	0	0	1000,00	1000,00
0	0	0	0	0	0	0	1000,00	1000,00
0	0	0	0	0	0	0	1000,00	1000,00
0	0	0	0	0	0	0	1000,00	1000,00
0	0	0	0	0	0	0	1000,00	1000,00
0	0	0	0	0	0	0	1000,00	1000,00
0	0	0	0	0	0	0	1000,00	1000,00
0	0	0	0	0	0	0	1000,00	1000,00
0	0	0	0	0	0	0	1000,00	1000,00
0	0	0	0	0	0	0	1000,00	1000,00
0	0	0	0	0	0	0	1000,00	1000,00
0	0	0	0	0	0	0	1000,00	1000,00
1	0	0	0	0	0	1	2,00	1,00
0	0	0	0	0	0	0	1000,00	1000,00
0	0	0	0	0	0	0	1000,00	1000,00
0	0	0	0	0	0	0	1000,00	1000,00
0	0	0	0	0	0	0	1000,00	1000,00
0	0	0	0	0	0	0	1000,00	1000,00
0	99	0	0	0	0	#NULL!	1,00	3,00
0	0	0	0	0	0	0	1000,00	1000,00
1	1	0	0	0	0	2	2,00	2,00
0	1	0	0	0	0	1	1,00	1,00
0	0	0	0	0	0	0	1000,00	1000,00
0	1	0	0	0	0	1	2,00	3,00
0	0	0	0	0	0	0	1000,00	1000,00
0	0	0	0	0	0	0	1000,00	1000,00
0	0	0	0	0	0	0	1000,00	1000,00
0	0	0	0	0	0	0	1000,00	1000,00
0	0	0	0	0	0	0	1000,00	1000,00
0	0	0	0	0	0	0	1000,00	1000,00
0	1	0	0	0	0	1	2,00	3,00
0	0	0	0	0	0	0	1000,00	1000,00
0	0	0	0	0	0	0	1000,00	1000,00
0	0	0	0	0	0	0	2,00	1000,00
0	0	0	0	0	0	0	1000,00	1000,00
#NULL!	#NULL!	#NULL!	#NULL!	#NULL!	#NULL!	#NULL!	1000,00	1000,00
0	0	0	0	0	0	0	1000,00	1000,00
0	0	0	0	0	0	0	1000,00	1000,00
0	0	0	0	0	0	0	1000,00	1000,00
0	0	0	0	0	0	0	1000,00	1000,00
0	0	0	0	0	0	0	1000,00	1000,00
0	0	0	0	0	0	0	1000,00	1000,00

0	0	0	0	0	0	0	1000,00	1000,00
0	0	0	0	0	0	0	1000,00	1000,00
0	0	0	0	0	0	0	1000,00	1000,00
0	0	0	0	0	0	0	1000,00	1000,00
0	0	0	0	0	0	0	1000,00	1000,00
0	0	0	0	0	0	0	1000,00	1000,00
0	0	0	0	0	0	0	1000,00	1000,00
0	0	0	0	0	0	0	1000,00	1000,00
0	0	0	0	0	0	0	1000,00	1000,00
0	0	0	0	0	0	0	1000,00	1000,00
0	0	0	0	0	0	0	1000,00	1000,00
0	0	0	0	0	0	0	1000,00	1000,00
0	0	0	0	0	0	0	1000,00	1000,00
0	0	0	0	0	0	0	1000,00	1000,00
0	0	0	0	0	0	0	1000,00	1000,00
0	0	0	0	0	0	0	1000,00	1000,00
0	0	0	0	0	0	0	1000,00	1000,00
0	0	0	0	0	0	0	1000,00	1000,00
0	0	0	0	0	0	0	1000,00	1000,00
0	0	0	0	0	0	0	1000,00	1000,00
0	0	0	0	0	0	0	1000,00	1000,00
0	0	0	0	0	0	0	1000,00	1000,00
0	0	0	0	0	0	0	1000,00	1000,00
0	0	0	0	0	0	0	1000,00	1000,00
0	0	0	0	0	0	0	1000,00	1000,00
1	0	0	0	0	0	1	2,00	1,00
1	1	0	1	0	0	3	2,00	1,00
0	0	0	0	0	0	0	1000,00	1000,00
0	0	0	0	0	0	0	1000,00	1000,00
0	0	0	0	0	0	0	1000,00	1000,00
1	1	1	0	0	0	3	1,00	1,00
0	0	0	0	0	0	0	1000,00	1000,00
0	1	0	0	0	0	1	1,00	1,00
0	0	0	0	0	0	0	1000,00	1000,00
0	1	0	0	0	0	1	1,00	1,00
0	0	0	0	0	0	0	1000,00	1000,00
0	0	0	0	0	0	0	1000,00	1000,00
0	0	0	0	0	0	0	1000,00	1000,00
0	0	0	0	0	0	0	1000,00	1000,00
1	1	0	0	0	0	2	2,00	1,00
1	1	0	0	0	0	2	1,00	1,00
0	1	0	0	0	0	1	1,00	1,00
0	0	0	0	0	0	0	1000,00	1000,00
0	0	0	0	0	0	0	1000,00	1000,00
0	0	0	0	0	0	0	1000,00	1000,00
0	0	0	0	0	0	0	1000,00	1000,00
0	0	0	0	0	0	0	1000,00	1000,00
1	0	0	0	0	0	1	1,00	1,00
0	0	0	0	0	0	0	1000,00	1000,00
0	0	0	0	0	0	0	1000,00	1000,00

0	0	0	0	0	0	0	1000,00	1000,00
1	0	0	0	0	0	1	1,00	1,00
1	1	0	0	0	0	2	3,00	3,00
0	0	0	0	0	0	0	1000,00	1000,00
0	0	0	0	0	0	0	1000,00	1000,00
0	0	0	0	0	0	0	1000,00	1000,00
0	0	0	0	0	0	0	1000,00	1000,00
0	0	0	0	0	0	0	1000,00	1000,00
0	0	0	0	0	0	0	1000,00	1000,00
0	0	0	0	0	0	0	1000,00	1000,00
0	0	0	0	0	0	0	1000,00	1000,00
0	0	0	0	0	0	0	1000,00	1000,00
0	0	0	0	0	0	0	1000,00	1000,00
1	1	1	1	0	0	4	2,00	1,00
0	0	0	0	0	0	0	1000,00	1000,00
1	0	0	0	0	0	1	1000,00	1000,00
0	0	0	0	0	0	0	1000,00	1000,00
0	0	0	0	0	0	0	1000,00	1000,00
1	0	0	1	0	0	2	2,00	1,00
1	1	1	1	0	0	4	2,00	1,00
1	0	0	0	0	0	1	2,00	1,00
0	0	0	0	0	0	0	1000,00	1000,00
0	0	0	0	0	0	0	1000,00	1000,00
0	0	0	0	0	0	0	1000,00	1000,00
0	1	0	0	0	0	1	2,00	3,00
0	0	0	0	0	0	0	1000,00	1000,00
0	0	0	0	0	0	0	1000,00	1000,00
0	0	0	0	0	0	0	1000,00	1000,00
0	0	0	0	0	0	0	1000,00	1000,00
0	0	0	0	0	0	0	1000,00	1000,00
0	0	0	0	0	0	0	1000,00	1000,00
0	0	0	0	0	0	0	1000,00	1000,00

istuisactsha	physicalfor	Physicalfor	Everforced	Sexualinter	sexualinter	partneruse	partnerma	partnerforc
2,00	0	0	1	0	0	0	0	0
2,00	0	0	0	0	0	0	0	0
2,00	0	0	0	0	0	0	1	0
99,00	#NULL!	#NULL!	#NULL!	#NULL!	#NULL!	#NULL!	#NULL!	#NULL!
99,00	1	1	1	1	1	1	1	1
1000,00	0	0	0	0	0	0	0	0
1000,00	0	0	0	1	0	0	1	1
1000,00	1	1	1	0	1	1	1	0
1000,00	1	0	1	0	1	1	1	1
2,00	0	0	0	1	1	1	0	0
1000,00	0	0	0	0	0	0	0	0
1000,00	0	0	1	0	0	0	0	0
2,00	1	1	0	0	0	0	0	0
2,00	1	0	0	0	1	0	0	0
2,00	0	0	0	1	0	0	0	1
1000,00	0	0	0	0	0	0	0	0
1000,00	0	0	1	0	0	1	0	0
1000,00	0	0	0	0	0	1	1	0
2,00	0	0	0	0	0	0	0	0
1,00	0	0	0	1	1	1	0	0
1,00	1	1	1	0	1	1	0	1
1,00	1	0	0	0	0	0	0	0
1000,00	0	0	0	0	0	1	0	0
1000,00	0	0	0	0	0	0	0	1
1000,00	0	0	1	1	1	0	0	0
1000,00	1	0	1	0	1	1	0	0
1000,00	0	0	0	0	0	0	0	0
1000,00	0	0	0	0	0	0	0	0
1,00	0	1	0	0	1	1	1	1
1000,00	0	0	1	1	1	1	1	1
1000,00	0	0	0	0	0	0	0	0
1000,00	0	0	0	0	0	0	0	0
1,00	1	1	1	#NULL!	#NULL!	1	0	1
2,00	1	1	1	1	1	1	0	0
1000,00	1	0	1	0	0	0	0	1
2,00	1	0	1	0	1	1	1	0
2,00	0	0	0	0	0	0	0	0
1000,00	1	0	#NULL!	0	1	0	1	0
1000,00	0	0	0	0	1	0	0	0
2,00	0	0	0	0	0	0	0	1
1000,00	0	0	0	0	0	0	0	0
1000,00	1	0	1	0	0	0	0	0
1,00	0	0	1	1	1	0	0	#NULL!
1000,00	0	0	0	0	0	0	0	0
1000,00	1	1	1	1	1	1	0	0
1000,00	0	0	0	0	0	0	0	0
1,00	0	0	0	1	1	0	0	0
1000,00	1	1	1	0	0	0	0	0
1000,00	0	0	0	0	0	0	0	0

99,00	0	0	0	0	0	0	1	0
1000,00	0	0	0	0	0	0	0	0
1000,00	0	0	0	0	0	0	1	1
1000,00	0	0	0	0	0	0	0	0
1000,00	0	0	0	0	0	0	0	0
1,00	0	0	0	1	0	0	0	0
2,00	0	0	0	0	0	0	0	0
1000,00	0	0	0	0	0	0	0	0
1,00	0	0	0	0	0	0	0	0
1000,00	0	0	0	0	0	0	0	0
1000,00	0	0	0	0	0	0	0	0
1000,00	0	0	0	0	0	0	0	0
1000,00	0	0	0	0	0	0	0	0
1,00	0	0	1	0	0	1	1	0
1000,00	0	0	0	0	1	0	0	0
1,00	0	0	0	0	0	0	0	0
1,00	0	0	0	0	1	0	0	0
1000,00	0	0	0	0	0	0	0	0
1000,00	0	0	0	0	0	0	0	0
2,00	0	0	0	1	0	0	0	0
1000,00	0	0	0	0	0	0	0	0
2,00	0	0	0	1	1	0	0	0
1000,00	0	0	0	0	0	0	0	0
1,00	0	0	0	1	0	0	0	0
1000,00	0	0	0	0	0	0	0	0
1000,00	0	0	0	0	0	0	0	0
2,00	0	0	0	0	0	0	0	0
1000,00	0	0	0	0	0	0	0	0
2,00	0	0	0	1	0	0	0	0
2,00	0	1	1	0	0	1	0	0
1000,00	0	0	0	1	0	0	0	0
1000,00	0	0	0	0	0	0	0	0
1000,00	0	0	0	0	0	0	0	0
2,00	0	0	0	0	1	0	1	0
1,00	0	0	0	0	0	0	0	0
1000,00	0	0	0	0	0	0	0	0
1,00	0	0	0	0	0	0	0	0
1000,00	0	0	0	0	0	0	0	0
1000,00	1	0	1	1	1	0	0	0
1,00	0	0	0	0	0	0	0	0
1000,00	0	0	0	0	0	0	0	0
1000,00	0	0	0	0	0	0	0	0
1000,00	0	0	0	0	0	0	0	0
1000,00	0	0	0	0	0	0	0	0
1000,00	0	0	0	0	0	0	0	0
2,00	0	0	0	0	0	0	0	0
1000,00	0	0	0	0	0	0	0	0
1,00	0	0	0	0	0	0	0	0
1000,00	0	0	0	0	0	0	0	0
2,00	0	0	0	0	0	0	0	0

1000,00	0	0	0	0	0	0	0	0
1000,00	0	0	0	0	0	0	0	0
1000,00	1	0	1	0	0	0	0	0
1000,00	0	0	0	0	0	0	0	0
1000,00	0	0	0	0	0	0	0	0
1000,00	0	0	0	0	0	0	0	0
1000,00	0	0	0	0	0	0	0	0
1000,00	0	0	0	0	0	0	0	0
1000,00	0	0	0	0	0	0	0	0
1000,00	0	0	0	0	0	0	0	0
1000,00	0	0	0	0	0	0	0	0
1000,00	0	0	0	0	0	0	0	0
1000,00	0	0	0	0	0	0	0	0
1,00	1	1	1	0	1	0	0	1
1000,00	0	0	1	1	1	1	0	1
1000,00	0	0	0	0	0	0	0	0
1000,00	0	0	0	0	0	0	0	0
1000,00	0	0	0	0	0	0	0	0
1000,00	0	0	0	0	0	0	0	0
1000,00	0	0	0	0	0	0	0	0
1000,00	0	0	0	0	0	0	0	0
1000,00	0	0	0	0	0	0	0	0
1000,00	0	0	0	0	0	0	0	0
1000,00	0	0	0	0	0	0	0	0
2,00	0	0	0	0	0	0	0	0
1000,00	0	0	0	0	0	0	0	0
1,00	0	0	0	1	0	1	0	0
2,00	0	0	1	0	0	0	0	0
1000,00	0	0	0	0	0	0	0	0
1000,00	0	0	0	0	0	0	0	0
1000,00	1	0	1	1	0	0	0	0
1,00	0	0	0	0	0	0	0	0
1000,00	0	0	0	0	0	1	0	0
1000,00	0	0	0	0	0	0	0	0
1000,00	0	0	0	0	0	0	0	0
1000,00	0	0	0	0	1	0	0	0
1000,00	0	0	0	0	0	0	0	0
1000,00	0	0	0	0	0	0	0	0
1000,00	0	0	0	0	0	0	0	0
1000,00	0	0	0	0	0	0	0	0
1000,00	0	0	0	1	0	0	0	1
1000,00	0	0	0	0	0	0	0	0
1000,00	0	1	0	0	0	0	0	0
1000,00	0	0	0	0	0	0	0	0
1000,00	0	0	0	0	0	1	0	0
1000,00	0	0	0	0	0	0	0	0
1000,00	0	0	0	1	0	0	0	1
1000,00	0	0	0	0	0	0	0	0
1,00	0	0	0	0	0	0	1	0
1000,00	0	0	0	0	0	0	0	0

1000,00	0	0	0	1	1	0	0	0
1000,00	0	0	0	0	0	0	0	0
1,00	0	0	0	1	0	0	0	0
1000,00	0	0	0	0	0	0	0	0
1000,00	0	0	0	0	0	0	0	0
1000,00	0	0	0	0	0	0	0	0
1000,00	0	0	0	0	0	0	0	0
1000,00	1	0	1	1	0	1	1	1
1000,00	0	0	0	0	0	0	0	0
1000,00	0	0	0	0	0	0	0	0
1,00	0	0	0	1	1	1	0	1
1000,00	0	0	0	0	0	0	0	0
1000,00	0	0	0	0	0	0	0	0
1000,00	0	0	0	0	0	0	0	0
1000,00	0	0	0	0	0	0	0	0
1000,00	0	0	1	0	0	0	0	0
1000,00	0	0	0	0	0	0	0	0
1000,00	0	0	0	0	0	0	0	0
1000,00	0	0	0	0	0	0	0	0
1000,00	0	0	0	0	0	0	0	0
1000,00	0	0	0	0	0	0	0	0
1000,00	0	0	0	0	0	0	0	0
2,00	0	0	0	0	0	0	0	0
2,00	0	0	0	0	0	0	0	0
1000,00	0	0	0	0	0	0	0	0
1000,00	0	0	0	0	0	0	0	0
1000,00	0	0	0	0	0	0	0	0
1000,00	0	0	0	0	0	0	0	0
1,00	0	0	0	1	0	0	0	0
1000,00	0	0	0	0	0	0	0	0
1000,00	0	0	0	0	0	0	0	0
1000,00	0	0	0	0	0	0	0	0
1000,00	0	0	0	0	0	0	0	0
2,00	1	1	1	1	1	0	0	0
1000,00	0	0	0	0	0	0	0	0
1000,00	0	0	0	0	0	0	0	0
1000,00	0	0	0	0	0	0	0	0
1000,00	0	0	0	0	0	0	0	0
1000,00	0	0	0	0	0	0	0	0
1000,00	1	1	1	1	1	0	1	0
1000,00	0	0	0	0	0	0	0	0
1000,00	0	0	0	0	0	0	0	0
1000,00	0	0	0	0	0	0	0	0
1000,00	0	0	0	0	0	0	0	0
1,00	0	0	0	0	0	0	0	0
1000,00	0	0	0	0	0	0	0	0
1000,00	0	0	0	0	0	0	0	0
1000,00	0	0	0	0	0	0	0	0
1000,00	0	0	0	0	0	0	0	#NULL!
1000,00	0	0	0	0	0	0	0	0

1000,00	0	0	0	0	0	0	0	0
1000,00	0	0	0	0	0	0	0	0
1000,00	0	0	0	0	0	0	0	0
1000,00	0	0	0	0	0	0	0	0
1000,00	0	0	0	0	0	0	0	0
1000,00	0	0	0	0	0	0	0	0
1000,00	0	0	0	0	0	0	0	0
1,00	0	0	0	0	0	0	0	0
1000,00	0	0	0	0	0	0	0	0
1000,00	0	0	0	0	0	0	0	0
1000,00	0	0	0	0	0	0	0	0
1000,00	0	0	0	0	0	0	0	0
1000,00	0	0	0	0	0	0	0	0
1000,00	0	0	0	0	0	0	0	0
1000,00	0	0	0	0	0	0	0	0
1000,00	1	0	0	0	0	0	0	0
1000,00	0	0	0	0	0	0	0	0
1,00	0	0	0	0	1	1	1	1
1000,00	0	0	0	0	0	0	0	0
1000,00	0	0	0	0	0	0	0	0
1000,00	0	0	0	0	0	0	0	0
1000,00	0	0	0	0	0	0	0	0
1000,00	0	0	0	0	0	0	0	0
1,00	0	0	0	0	0	0	0	0
1000,00	0	0	1	0	0	0	0	0
1000,00	0	0	0	0	0	0	0	0
1000,00	0	0	0	0	0	0	0	0
1,00	0	0	0	1	1	0	0	0
1,00	0	0	0	0	0	0	0	0
1000,00	0	0	0	0	0	0	0	0
1000,00	0	0	0	0	0	0	0	0
1000,00	0	0	0	0	0	0	0	0
1000,00	1	0	1	0	0	0	0	0
1,00	0	0	0	0	0	0	0	0
1,00	1	0	1	0	0	0	1	1
1,00	1	0	0	0	0	0	0	0
1000,00	0	0	0	0	0	0	0	0
1000,00	0	0	0	0	0	0	0	0
2,00	1	0	1	0	0	1	0	0
1000,00	0	0	0	0	0	0	0	0
2,00	0	0	0	0	1	0	0	0
1,00	0	0	1	1	1	0	0	1
1000,00	0	0	1	0	0	0	0	0
1000,00	0	0	0	0	0	0	0	0
1000,00	0	0	0	0	0	0	0	0
1000,00	0	0	0	0	0	0	0	0
1000,00	0	0	0	0	0	0	0	0
1000,00	0	0	0	0	0	0	0	0
1000,00	1	1	1	1	1	1	0	0
1000,00	0	0	0	0	0	1	1	1

1000,00	0	0	0	0	0	0	0	0
1000,00	0	0	0	0	0	0	0	0
1000,00	0	0	0	0	0	0	0	0
1000,00	0	0	0	0	0	0	0	0
1000,00	0	0	0	0	0	0	0	0
1000,00	1	0	0	0	0	0	0	0
1000,00	0	0	1	0	0	0	0	0
1000,00	0	1	1	0	0	0	0	0
1000,00	0	0	0	0	0	0	0	0
1000,00	0	0	1	0	0	0	0	0
1,00	0	0	0	0	0	0	0	0
1000,00	0	0	0	0	0	0	0	0
1000,00	0	0	0	0	0	0	0	0
1,00	0	1	0	0	0	0	0	0
1000,00	1	1	1	1	0	1	0	1
1000,00	0	1	1	1	0	0	0	0
1000,00	0	0	0	0	0	0	0	0
1000,00	0	0	0	0	0	0	0	0
1000,00	0	0	0	0	0	0	0	0
1000,00	0	1	0	0	0	0	0	0
1000,00	0	0	0	0	0	0	0	0
1000,00	0	0	0	0	0	0	0	0
1,00	0	1	0	1	1	0	0	1
1000,00	0	0	0	0	0	0	0	0
1000,00	0	0	0	0	0	0	0	0
1000,00	0	0	0	0	0	0	0	0
1000,00	0	0	0	0	0	0	0	0
1000,00	0	0	0	0	0	0	0	0
1000,00	0	0	0	0	0	0	0	0
2,00	0	1	1	0	0	0	0	0
1000,00	0	0	0	0	0	0	0	0
1000,00	0	0	0	0	0	0	0	0
1000,00	0	0	0	0	0	0	0	0
1000,00	0	0	0	0	0	0	0	0
1000,00	0	0	0	0	0	0	0	0
1,00	0	0	0	0	0	0	0	0
1000,00	0	0	0	0	0	0	0	0
1000,00	0	0	0	0	0	0	0	0
1,00	0	0	1	0	0	0	1	0
1000,00	0	0	0	0	0	0	0	0
1000,00	0	0	0	0	0	0	0	0
1000,00	0	0	0	0	0	0	0	0
1000,00	0	0	0	0	0	0	0	0
1000,00	0	0	0	0	0	0	0	0
1000,00	0	0	0	0	0	0	0	0
1000,00	0	0	0	0	0	0	0	0
1,00	0	0	0	0	0	0	0	0
1000,00	0	0	0	0	0	0	0	0
1000,00	0	0	0	0	0	0	0	0
1000,00	0	0	0	0	0	0	0	0

1000,00	0	0	0	0	0	0	0	0
1000,00	0	0	0	1	0	0	0	0
1,00	0	0	0	0	0	0	0	0
2,00	0	0	0	0	0	0	0	0
1000,00	0	0	0	0	0	0	0	0
1000,00	0	0	0	0	0	0	0	0
1000,00	0	0	0	0	0	0	0	0
2,00	0	0	0	0	0	0	0	0
1000,00	0	0	0	0	0	0	0	0
1000,00	0	0	0	0	0	0	0	0
1000,00	0	0	0	0	0	0	0	0
1000,00	0	0	0	0	0	0	0	0
1000,00	0	0	0	0	0	0	0	0
1000,00	0	0	0	0	0	0	0	0
1000,00	0	0	0	0	0	0	0	0
1000,00	0	0	0	0	0	0	0	0
1000,00	0	0	0	0	0	1	0	0
1000,00	0	0	0	0	0	0	0	0
1000,00	0	0	0	0	0	0	0	0
1000,00	0	0	0	0	0	0	0	0
2,00	0	0	0	0	0	0	0	0
1000,00	0	0	0	0	0	0	0	0
1000,00	0	0	0	0	0	0	0	0
1000,00	0	0	0	0	0	0	0	0
1000,00	0	0	0	0	0	0	0	0
1000,00	0	0	0	0	0	0	0	0
1,00	0	0	0	0	0	0	0	0
1000,00	0	0	0	0	0	0	0	0
1,00	0	0	0	0	0	0	0	0
1,00	0	0	0	0	0	0	0	0
1000,00	0	0	0	0	0	0	0	0
2,00	0	0	0	0	0	0	0	0
1000,00	0	0	0	0	0	0	0	0
1000,00	0	0	0	0	0	0	0	0
1000,00	0	0	0	0	0	0	0	0
1000,00	0	0	0	0	0	0	0	0
1000,00	0	0	0	0	0	0	0	0
1000,00	0	0	0	0	0	0	0	0
2,00	1	1	1	0	0	0	0	0
1000,00	0	0	0	0	0	0	0	0
1000,00	0	0	0	0	0	0	0	0
1000,00	#NULL!	0	0	0	0	0	0	0
1000,00	0	0	0	0	0	0	0	0
1000,00	#NULL!	#NULL!	#NULL!	#NULL!	#NULL!	#NULL!	#NULL!	#NULL!
1000,00	0	0	0	0	0	0	0	0
1000,00	0	0	0	0	0	0	0	0
1000,00	0	0	0	0	0	0	0	0
1000,00	0	0	0	0	0	0	0	0
1000,00	0	0	0	0	0	0	0	0
1000,00	1	0	0	0	0	0	0	0

1000,00	0	0	0	0	0	0	0	0
1000,00	0	0	0	1	0	0	0	0
1000,00	0	0	0	0	0	0	0	0
1000,00	0	0	0	1	0	1	0	0
1000,00	0	0	0	0	0	0	0	0
1000,00	0	0	0	0	0	1	0	0
1000,00	0	0	0	0	0	0	0	0
1000,00	0	0	0	0	0	0	0	0
1000,00	0	0	0	0	0	0	0	0
1000,00	0	0	0	0	0	0	0	0
1000,00	0	0	0	0	0	0	0	0
1000,00	0	0	0	0	0	0	0	0
1000,00	0	0	0	0	0	0	0	0
1000,00	0	0	0	0	0	1	0	1
1000,00	0	0	0	0	0	0	0	0
1000,00	0	0	0	0	0	0	0	0
1000,00	0	0	0	0	0	0	0	0
1000,00	0	0	1	0	0	0	0	0
1000,00	0	0	0	0	0	0	0	0
1000,00	0	0	0	0	0	0	0	0
1000,00	0	0	0	0	0	0	0	0
1000,00	0	0	0	0	0	0	0	0
1000,00	0	0	0	0	0	0	0	0
1000,00	0	0	0	0	0	0	1	0
1000,00	0	0	0	1	1	0	0	0
1,00	0	0	0	0	0	0	0	0
1,00	0	1	1	0	0	0	0	0
1000,00	0	0	0	0	0	0	0	0
1000,00	1	1	1	1	1	1	1	0
1000,00	1	1	1	0	0	0	0	0
2,00	0	0	0	0	0	0	0	0
1000,00	0	0	0	0	0	0	0	0
1,00	1	1	1	1	1	1	0	0
1000,00	1	0	1	1	0	1	1	0
1,00	1	0	1	1	1	0	0	0
1000,00	0	0	0	0	0	0	0	0
1000,00	0	0	0	0	0	0	0	0
1000,00	0	0	0	0	0	0	0	0
1000,00	0	0	0	1	1	0	1	1
1,00	1	0	1	1	1	1	0	0
1,00	0	0	0	0	0	0	0	0
1,00	0	0	0	0	0	0	0	0
1000,00	0	0	0	0	0	1	0	0
1000,00	0	0	0	0	0	0	0	0
1000,00	0	0	0	0	0	0	0	0
1000,00	0	0	0	0	0	0	0	0
1000,00	0	0	0	0	0	0	0	0
2,00	0	1	0	1	0	0	0	0
1000,00	0	0	0	0	0	0	0	0
1000,00	0	0	0	0	0	0	0	0

1000,00	0	0	0	0	0	0	0	0
2,00	0	0	0	0	0	0	0	0
2,00	1	0	1	0	0	0	0	0
1000,00	0	0	0	0	0	0	0	0
1000,00	0	0	0	0	0	0	0	0
1000,00	0	0	0	0	0	0	0	0
1000,00	0	0	0	0	0	0	0	0
1000,00	0	0	0	0	0	0	0	0
1000,00	0	0	0	0	0	0	0	0
1000,00	0	0	0	0	0	0	0	0
1000,00	0	0	0	0	0	0	0	0
1000,00	0	1	1	1	0	0	0	1
1000,00	0	0	0	0	0	0	0	0
1,00	0	0	0	0	0	1	0	1
1000,00	0	0	0	1	1	1	0	0
1000,00	0	0	0	0	0	0	1	1
1000,00	0	0	0	0	0	0	0	0
1000,00	0	0	0	0	0	0	0	0
1,00	0	0	0	0	0	0	0	0
1,00	1	1	0	1	1	0	0	0
2,00	0	0	0	0	0	0	0	0
1000,00	1	0	1	0	0	1	0	1
1000,00	0	0	0	0	0	0	0	0
1000,00	0	0	0	0	0	0	0	0
1,00	1	1	1	0	0	1	0	1
1000,00	0	0	0	1	0	0	0	0
1000,00	0	0	0	0	0	0	0	0
1000,00	0	0	0	0	0	0	0	0
1000,00	0	0	0	0	0	0	0	0
1000,00	0	0	0	0	0	0	0	0
1000,00	0	0	0	0	0	0	0	0
1000,00	0	0	0	0	0	0	0	0

TOT_SEXV	Whodidsex	Howoldisth	istuisactsha	partnercall	Partnerswo	Partneryell	Partnertrea	Partnertolc
1	99	4,00	2,00	0	0	0	0	0
0	99	99,00	2,00	0	0	1	0	0
1	99	99,00	2,00	0	0	0	0	0
#NULL!	99	99,00	99,00	#NULL!	#NULL!	#NULL!	#NULL!	#NULL!
8	2	1,00	1,00	1	1	1	0	0
0	1000	1000,00	1000,00	0	0	0	0	0
3	2	3,00	1,00	0	0	1	0	1
6	2	2,00	1,00	0	0	1	0	0
6	2	1,00	2,00	0	0	0	1	0
3	1	1,00	2,00	0	0	0	0	0
0	1000	1000,00	1000,00	1	1	0	0	1
1	1	99,00	2,00	0	0	0	0	1
2	2	1,00	99,00	0	0	0	0	0
2	2	1,00	1,00	0	0	0	0	0
2	2	1,00	2,00	1	1	1	0	0
0	1000	1000,00	1000,00	0	0	0	0	0
2	2	1,00	1,00	0	0	0	0	0
2	1000	1000,00	1000,00	0	0	#NULL!	0	0
0	1000	1000,00	1000,00	0	0	0	0	0
3	2	1,00	2,00	1	1	1	1	0
6	2	1,00	2,00	0	0	1	1	0
1	1	1,00	1,00	0	0	1	0	0
1	2	1,00	1,00	0	0	0	0	0
1	1	1,00	1,00	0	0	1	0	0
3	1	1,00	2,00	0	0	0	1	0
4	1	3,00	1,00	0	1	0	0	0
0	1000	1000,00	1000,00	0	0	0	0	0
0	1000	1000,00	1000,00	0	0	#NULL!	0	0
5	1000	3,00	1,00	0	0	1	0	0
6	1	1,00	1,00	0	0	1	1	0
0	1000	1000,00	1000,00	0	#NULL!	0	0	0
0	1000	1000,00	1000,00	1	0	#NULL!	0	0
#NULL!	2	1,00	1,00	1	1	1	0	0
6	2	2,00	2,00	1	1	1	1	1
3	2	1,00	2,00	0	0	0	0	0
5	2	1,00	2,00	1	1	1	1	1
0	1000	1000,00	1000,00	1	0	1	0	0
#NULL!	99	99,00	99,00	0	0	0	0	0
1	1	1,00	1,00	0	0	0	0	0
1	3	1,00	2,00	1	0	1	0	0
0	1000	1000,00	1000,00	0	0	0	0	0
2	1	1,00	1,00	0	0	1	0	0
#NULL!	1	2,00	1,00	1	1	1	#NULL!	0
0	1000	1000,00	1000,00	0	0	0	0	0
6	2	3,00	2,00	1	0	1	0	1
0	1000	1000,00	1000,00	0	0	0	0	0
2	1	1,00	1,00	0	0	1	0	1
3	2	1,00	1,00	0	0	0	0	0
0	1000	1000,00	1000,00	1	0	1	0	0

1	2	3,00	1,00	1	1	1	1	0
0	1000	1000,00	1000,00	0	0	1	0	0
2	1	1,00	1,00	0	0	0	0	0
0	1000	1000,00	1000,00	0	0	0	0	0
0	1000	1000,00	1000,00	1	1	1	0	0
1	1	99,00	1,00	0	0	0	0	0
0	1000	1000,00	1000,00	1	1	1	1	1
0	1000	1000,00	1000,00	0	1	0	1	1
0	1000	1000,00	1000,00	0	0	1	0	0
0	1000	1000,00	1000,00	0	0	0	0	0
0	1000	1000,00	1000,00	0	0	0	0	0
0	1000	1000,00	1000,00	0	0	1	1	0
0	1000	1000,00	1000,00	0	0	1	0	0
3	3	3,00	2,00	1	0	0	1	0
1	1000	1000,00	1000,00	0	0	0	0	0
0	1000	1000,00	1000,00	0	0	0	0	0
1	2	1,00	2,00	1	0	1	0	0
0	1000	1000,00	1000,00	0	0	0	0	0
0	1000	1000,00	1000,00	0	0	0	0	0
1	2	1,00	2,00	1	0	1	0	0
0	1000	1000,00	1000,00	0	0	1	0	0
2	1	3,00	2,00	0	0	0	1	1
0	1000	1000,00	1000,00	0	0	0	0	0
1	2	1,00	2,00	0	0	0	0	0
0	1000	1000,00	1000,00	0	0	0	0	0
0	1000	1000,00	1000,00	0	0	0	0	0
0	1000	1000,00	1000,00	0	0	0	0	0
0	1000	1000,00	1000,00	0	0	0	0	0
1	1	1,00	2,00	1	1	0	0	0
3	1	1,00	1,00	0	0	0	0	0
1	1000	1000,00	1000,00	0	0	1	0	0
0	1000	1000,00	1000,00	0	0	0	0	0
0	1000	1000,00	1000,00	0	0	0	0	0
2	2	3,00	2,00	1	1	1	1	1
0	1000	1000,00	1000,00	0	0	1	0	1
0	1000	1000,00	1000,00	0	0	0	0	0
0	1000	1000,00	1000,00	1	0	1	0	0
0	1000	1000,00	1000,00	1	0	1	0	1
4	2	3,00	1,00	1	0	1	0	0
0	1000	1000,00	1000,00	0	0	1	0	0
0	1000	1000,00	1000,00	0	0	0	0	0
0	1000	1000,00	1000,00	0	0	0	0	0
0	1000	1000,00	1000,00	0	0	1	0	0
0	1000	1000,00	1000,00	0	0	0	0	0
0	1000	1000,00	1000,00	1	1	0	0	0
0	1000	1000,00	1000,00	1	1	1	1	1
0	1000	1000,00	1000,00	0	0	0	0	0
0	1000	1000,00	1000,00	0	0	1	1	0
0	1000	1000,00	1000,00	0	0	0	0	0
0	1000	1000,00	1000,00	0	0	1	0	1

0	1000	1000,00	1000,00	0	0	0	0	0
0	1000	1000,00	1000,00	0	0	1	0	0
2	2	3,00	2,00	0	0	0	0	0
0	1000	1000,00	1000,00	0	0	0	0	0
0	1000	1000,00	1000,00	0	0	1	0	0
0	1000	1000,00	1000,00	0	0	0	0	0
0	1000	1000,00	1000,00	0	0	0	0	0
0	1000	1000,00	1000,00	0	0	0	0	0
0	1000	1000,00	1000,00	0	1	0	0	0
0	1000	1000,00	1000,00	0	0	0	0	0
0	1000	1000,00	1000,00	0	0	1	0	1
0	1000	1000,00	1000,00	0	0	1	0	0
0	1000	1000,00	1000,00	0	0	0	0	0
5	1	2,00	1,00	1	1	1	0	1
5	1	1,00	1,00	1	0	0	0	0
0	1000	1000,00	1000,00	0	0	0	0	0
0	1000	1000,00	1000,00	0	0	1	0	0
0	1000	1000,00	1000,00	0	0	0	0	0
0	1000	1000,00	1000,00	0	0	0	0	0
0	1000	1000,00	1000,00	0	0	0	0	0
0	1000	1000,00	1000,00	0	0	0	0	0
0	1000	1000,00	1000,00	0	0	1	1	0
0	1000	1000,00	1000,00	0	0	0	0	0
0	1000	1000,00	1000,00	0	0	0	0	0
0	1000	1000,00	1000,00	0	0	1	0	0
0	1000	1000,00	1000,00	0	0	0	0	0
2	1	1,00	1,00	1	1	1	1	0
1	2	1,00	2,00	1	0	1	0	0
0	1000	1000,00	1000,00	0	0	0	0	0
0	1000	1000,00	1000,00	0	0	0	0	0
3	2	1,00	1,00	0	0	0	0	0
0	1000	1000,00	1000,00	0	0	0	0	0
1	2	1,00	2,00	0	0	1	0	0
0	1000	1000,00	1000,00	0	0	0	0	0
0	1000	1000,00	1000,00	0	0	0	0	0
1	2	1,00	2,00	0	0	0	0	0
0	1000	1000,00	1000,00	0	0	1	0	1
0	1000	1000,00	1000,00	0	0	1	1	0
0	1000	1000,00	1000,00	0	0	1	0	0
0	1000	1000,00	1000,00	0	0	0	0	0
2	3	3,00	2,00	0	0	1	0	0
0	1000	1000,00	1000,00	0	0	0	0	0
1	1000	1000,00	1000,00	0	0	0	0	0
0	1000	1000,00	1000,00	0	0	0	0	0
1	1	1,00	1,00	0	0	1	1	0
0	1000	1000,00	1000,00	1	0	1	0	0
2	1	2,00	1,00	0	0	1	0	0
0	1000	1000,00	1000,00	0	0	0	0	0
1	2	1,00	1,00	0	0	0	1	0
0	1000	1000,00	1000,00	0	0	0	0	0

2	1	1,00	1,00	0	0	1	1	0
0	1000	1000,00	1000,00	0	0	0	0	0
1	1	1,00	1,00	0	0	0	0	0
0	1000	1000,00	1000,00	0	0	0	0	0
0	1000	1000,00	1000,00	0	0	0	1	0
0	1000	1000,00	1000,00	0	0	0	1	0
0	1000	1000,00	1000,00	0	0	0	0	0
6	2	1,00	1,00	0	0	1	0	1
0	1000	1000,00	1000,00	0	0	0	0	0
0	1000	1000,00	1000,00	0	0	1	0	0
4	2	1,00	2,00	1	0	0	1	0
0	1000	1000,00	1000,00	0	0	0	0	0
0	1000	1000,00	1000,00	0	0	0	0	0
0	1000	1000,00	1000,00	1	1	1	1	0
0	1000	1000,00	1000,00	0	0	0	0	0
1	1000	1000,00	1000,00	0	0	0	0	0
0	1000	1000,00	1000,00	0	0	0	0	0
0	1000	1000,00	1000,00	0	0	0	0	0
0	1000	1000,00	1000,00	0	0	0	0	0
0	1000	1000,00	1000,00	0	0	0	0	0
0	1000	1000,00	1000,00	0	0	0	0	0
0	1000	1000,00	1000,00	0	0	0	0	0
0	1000	1000,00	1000,00	0	0	1	0	0
0	1000	1000,00	1000,00	0	0	1	0	0
0	1000	1000,00	1000,00	0	0	1	0	0
0	1000	1000,00	1000,00	0	0	0	0	0
0	1000	1000,00	1000,00	0	0	0	0	0
0	1000	1000,00	1000,00	0	0	0	0	0
1	1	1,00	1,00	0	1	1	0	0
0	1000	1000,00	1000,00	0	0	0	0	0
0	1000	1000,00	1000,00	0	0	1	0	0
0	1000	1000,00	1000,00	0	0	1	0	0
0	1000	1000,00	1000,00	0	0	0	0	0
5	2	3,00	2,00	1	0	1	0	1
0	1000	1000,00	1000,00	0	0	0	0	0
0	1000	1000,00	1000,00	0	0	0	0	0
0	1000	1000,00	1000,00	0	0	0	0	0
0	1000	1000,00	1000,00	0	0	0	0	0
0	1000	1000,00	1000,00	0	0	0	0	0
6	2	1,00	2,00	0	0	0	0	0
0	1000	1000,00	1000,00	0	0	0	0	0
0	1000	1000,00	1000,00	0	0	1	0	0
0	1000	1000,00	1000,00	0	0	1	0	0
0	1000	1000,00	1000,00	0	0	1	0	0
0	1000	1000,00	1000,00	0	0	0	0	0
0	1000	1000,00	1000,00	0	0	0	0	0
0	1000	1000,00	1000,00	0	0	0	0	0
0	1000	1000,00	1000,00	0	0	0	0	0
#NULL!	1000	1000,00	1000,00	0	0	0	0	0
0	1000	1000,00	1000,00	0	0	0	0	0

0	1000	1000,00	1000,00	0	0	0	0	0
0	1000	1000,00	1000,00	0	0	0	0	0
0	1000	1000,00	1000,00	0	0	0	0	0
0	1000	1000,00	1000,00	0	0	0	0	0
0	1000	1000,00	1000,00	0	0	1	0	0
0	1000	1000,00	1000,00	0	0	1	0	0
0	1000	1000,00	1000,00	0	0	1	0	0
0	1000	1000,00	1000,00	1	0	0	0	0
0	1000	1000,00	1000,00	0	0	0	0	0
0	1000	1000,00	1000,00	0	0	0	0	0
0	1000	1000,00	1000,00	0	0	1	0	0
0	1000	1000,00	1000,00	0	0	0	0	0
0	1000	1000,00	1000,00	0	0	0	0	0
0	1000	1000,00	1000,00	0	0	0	0	0
0	1000	1000,00	1000,00	0	0	0	0	0
1	2	1,00	1,00	0	0	0	0	0
0	1000	1000,00	1000,00	0	0	0	0	0
4	1	1,00	1,00	1	1	1	0	0
0	1000	1000,00	1000,00	0	0	0	0	0
0	1000	1000,00	1000,00	0	0	1	1	0
0	1000	1000,00	1000,00	0	0	0	0	0
0	1000	1000,00	1000,00	0	0	0	0	0
0	1000	1000,00	1000,00	1	0	0	0	0
0	1	2,00	1,00	1	1	1	1	0
1	2	1,00	2,00	0	0	0	0	0
0	1000	1000,00	1000,00	0	0	1	0	0
0	1000	1000,00	1000,00	0	0	0	0	0
2	2	1,00	1,00	1	1	1	0	0
0	1000	1000,00	1000,00	0	0	1	0	0
0	1000	1000,00	1000,00	0	0	0	0	0
0	1000	1000,00	1000,00	0	0	1	0	0
0	1000	1000,00	1000,00	0	0	0	0	0
2	2	1,00	1,00	0	1	1	1	1
0	1000	1000,00	1000,00	0	0	1	0	1
4	2	2,00	1,00	1	1	1	0	1
1	2	1,00	1,00	0	0	1	1	1
0	1000	1000,00	1000,00	0	0	1	1	0
0	1000	1000,00	1000,00	0	1	1	0	0
3	2	1,00	2,00	0	1	1	0	0
0	1000	1000,00	1000,00	0	0	1	0	0
1	1	1,00	2,00	0	0	1	0	0
4	1	1,00	1,00	1	0	1	0	0
1	3	1,00	1,00	1	0	0	0	0
0	1000	1000,00	1000,00	0	0	0	0	0
0	1000	1000,00	1000,00	0	0	0	0	0
0	1000	1000,00	1000,00	0	0	1	0	0
0	1000	1,00	1000,00	0	0	1	0	0
0	1000	1000,00	1000,00	0	0	0	0	0
6	2	1,00	1,00	1	1	1	1	0
3	2	1,00	1,00	1	0	1	0	1

0	1000	1000,00	1000,00	0	0	1	0	0
0	1000	1000,00	1000,00	0	0	1	0	0
0	1000	1000,00	1000,00	0	0	0	0	0
0	1000	1000,00	1000,00	0	0	0	0	0
0	1000	1000,00	1000,00	0	0	0	0	0
1	1	1,00	1,00	0	0	1	0	0
1	2	1,00	2,00	0	0	1	0	1
2	2	1,00	1,00	0	0	0	0	0
0	1000	1000,00	1000,00	1	1	1	1	0
1	1	1,00	1,00	0	0	0	1	0
0	1000	1000,00	1000,00	0	0	1	0	0
0	1000	1000,00	1000,00	0	0	1	0	0
0	1000	1000,00	1000,00	0	0	0	0	0
1	1	1,00	1,00	0	0	0	0	0
6	2	3,00	1,00	1	0	1	1	1
3	2	2,00	2,00	0	1	1	1	0
0	1000	1000,00	1000,00	0	0	1	0	0
0	1000	1000,00	1000,00	0	0	0	0	0
0	1000	1000,00	1000,00	0	0	0	0	0
1	2	1,00	2,00	1	1	1	1	0
0	1000	1000,00	1000,00	0	0	0	0	0
0	1000	1000,00	1000,00	0	0	0	0	0
4	2	1,00	2,00	0	1	0	0	1
0	1000	1000,00	1000,00	0	0	0	0	0
0	1000	1000,00	1000,00	0	0	0	0	0
0	1000	1000,00	1000,00	0	0	0	0	0
0	1000	1000,00	1000,00	0	0	0	0	0
0	1000	1000,00	1000,00	0	0	0	0	0
0	1000	1000,00	1000,00	0	0	0	0	0
2	2	3,00	1,00	1	1	1	1	1
0	1000	1000,00	1000,00	0	0	0	0	0
0	2	2,00	1000,00	0	0	0	0	0
0	1000	1000,00	1000,00	0	0	0	0	0
0	1000	1000,00	1000,00	0	0	0	0	0
0	1000	1000,00	1000,00	0	0	0	0	0
0	1000	1000,00	1000,00	0	0	1	0	0
0	1000	1000,00	1000,00	0	0	0	0	0
0	1000	1000,00	1000,00	0	0	0	0	0
2	3	1,00	1,00	0	1	0	1	1
0	1000	1000,00	1000,00	0	0	0	0	0
0	1000	1000,00	1000,00	0	0	1	0	0
0	1000	1000,00	1000,00	1	1	1	0	1
0	1000	1000,00	1000,00	0	0	0	0	0
0	1000	1000,00	1000,00	0	0	0	0	0
0	1000	1000,00	1000,00	0	0	0	0	0
0	1000	1000,00	1000,00	0	0	0	0	0
0	1000	1000,00	1000,00	0	0	0	0	0
0	1000	1000,00	1000,00	0	0	1	0	0
0	1000	1000,00	1000,00	0	0	0	0	0
0	1000	1000,00	1000,00	0	0	0	0	0

0	1000	1000,00	1000,00	0	0	0	0	0
1	1	1,00	1,00	0	0	1	0	0
0	1000	1000,00	1000,00	0	0	0	0	0
0	1000	1000,00	1000,00	0	0	1	0	0
0	1000	1000,00	1000,00	0	0	1	0	0
0	1000	1000,00	1000,00	0	0	0	0	0
0	1000	1000,00	1000,00	0	0	0	0	0
0	1000	1000,00	1000,00	0	0	0	0	0
0	1000	1000,00	1000,00	0	0	1	0	0
0	1000	1000,00	1000,00	0	0	0	0	0
0	1000	1000,00	1000,00	0	0	0	0	0
0	1000	1000,00	1000,00	0	0	0	0	0
0	1000	1000,00	1000,00	0	0	0	0	0
0	1000	1000,00	1000,00	0	0	0	0	0
0	1000	1000,00	1000,00	0	0	1	0	0
0	1000	1000,00	1000,00	0	0	0	0	0
1	1	2,00	1,00	0	0	0	0	0
0	1000	1000,00	1000,00	0	0	0	0	0
0	1000	1000,00	1000,00	0	0	0	0	0
0	1000	1000,00	1000,00	0	0	0	0	0
0	1000	1000,00	1000,00	0	0	1	0	0
0	1000	1000,00	1000,00	0	0	0	0	0
0	1000	1000,00	1000,00	0	0	0	0	0
0	1000	1000,00	1000,00	0	0	0	0	0
0	1000	1000,00	1000,00	0	0	0	0	0
0	1000	1000,00	1000,00	0	0	0	0	0
0	1000	1000,00	1000,00	1	0	0	0	0
0	1000	1000,00	1000,00	0	0	1	0	0
0	1000	1000,00	1000,00	0	1	1	0	0
0	1000	1000,00	1000,00	0	0	1	0	0
0	1000	1000,00	1000,00	0	1	0	1	0
0	1000	1000,00	1000,00	0	0	1	0	0
0	1000	1000,00	1000,00	0	0	0	0	0
0	1000	1000,00	1000,00	0	0	0	0	0
0	1000	1000,00	1000,00	0	0	0	0	0
0	1000	1000,00	1000,00	0	0	0	0	0
0	1000	1000,00	1000,00	0	0	0	0	0
0	1000	1000,00	1000,00	0	0	0	0	0
3	2	3,00	2,00	1	0	0	0	0
0	1000	1000,00	1000,00	0	0	0	0	0
0	1000	1000,00	1000,00	0	0	0	0	0
#NULL!	1000	1000,00	1000,00	0	0	0	0	0
0	1000	1000,00	1000,00	0	0	1	0	0
#NULL!	1000	1000,00	1000,00	#NULL!	#NULL!	#NULL!	#NULL!	#NULL!
0	1000	1000,00	1000,00	0	0	0	0	0
0	1000	1000,00	1000,00	0	0	0	0	0
0	1000	1000,00	1000,00	0	0	0	0	0
0	1000	1000,00	1000,00	0	0	0	0	0
0	1000	1000,00	1000,00	0	0	0	0	0
1	3	1,00	1,00	1	0	0	0	0

0	1000	1000,00	1000,00	0	0	0	0	0
1	2	1,00	1,00	0	0	0	0	0
0	1000	1000,00	1000,00	0	0	0	0	0
2	1	1,00	1,00	1	1	1	0	0
0	1000	1000,00	1000,00	0	0	0	0	0
1	1	1,00	1,00	1	1	1	0	0
0	1000	1000,00	1000,00	0	0	0	0	0
0	1000	1000,00	1000,00	0	0	0	0	0
0	1000	1000,00	1000,00	0	0	0	0	0
0	1000	1000,00	1000,00	0	0	0	0	0
0	1000	1000,00	1000,00	0	0	0	0	0
0	1000	1000,00	1000,00	0	0	0	0	0
0	1000	1000,00	1000,00	0	0	0	0	0
2	1	3,00	1,00	0	0	1	0	1
0	1000	1000,00	1000,00	1	0	1	1	0
0	1000	1000,00	1000,00	0	0	0	0	0
0	1000	1000,00	1000,00	0	0	0	0	0
1	1	3,00	1,00	0	0	1	0	0
0	1000	1000,00	1000,00	1	1	1	0	1
0	1000	1000,00	1000,00	0	0	0	0	0
0	1000	1000,00	1000,00	0	0	1	0	0
0	1000	1000,00	1000,00	0	0	0	0	0
0	1000	1000,00	1000,00	0	0	0	0	0
1	2	1,00	1,00	0	0	1	0	0
2	1	1,00	2,00	1	1	1	0	0
0	1000	1000,00	1000,00	0	0	1	0	0
2	2	1,00	1,00	1	0	0	0	0
0	1000	1000,00	1000,00	0	0	0	0	0
7	2	1,00	2,00	0	0	1	0	0
3	2	1,00	2,00	1	0	1	1	0
0	1000	1000,00	1000,00	0	0	0	0	0
0	1000	1000,00	1000,00	0	0	0	0	0
6	2	3,00	2,00	1	0	1	1	0
5	2	1,00	2,00	0	0	1	1	0
4	1	1,00	1,00	1	0	1	0	0
0	1000	1000,00	1000,00	0	0	0	0	0
0	1000	1000,00	1000,00	0	0	0	0	0
0	1000	1000,00	1000,00	0	0	0	0	0
4	2	1,00	1,00	0	0	0	0	0
5	2	1,00	1,00	1	0	1	0	0
0	1000	1000,00	1000,00	1	1	1	1	1
0	1000	1000,00	1000,00	0	0	1	0	0
1	2	3,00	2,00	0	0	1	1	0
0	1000	1000,00	1000,00	0	0	0	0	0
0	1000	1000,00	1000,00	0	0	0	0	0
0	1000	1000,00	1000,00	0	0	0	0	0
0	1000	1000,00	1000,00	0	0	0	0	0
2	1	1,00	2,00	1	0	1	1	0
0	1000	1000,00	1000,00	0	0	1	0	1
0	1000	1000,00	1000,00	0	0	0	0	0

0	1000	1000,00	1000,00	0	0	0	0	0
0	1000	1000,00	1000,00	0	0	1	1	0
2	2	3,00	1,00	1	1	1	0	0
0	1000	1000,00	1000,00	0	0	0	0	0
0	1000	1000,00	1000,00	0	0	0	0	0
0	1000	1000,00	1000,00	0	0	0	0	0
0	1000	1000,00	1000,00	0	0	0	0	0
0	1000	1000,00	1000,00	0	0	1	0	0
0	1000	1000,00	1000,00	0	0	0	0	0
0	1000	1000,00	1000,00	0	0	0	0	0
0	1000	1000,00	1000,00	1	1	1	0	0
4	2	1,00	2,00	0	0	0	0	0
0	1000	1000,00	1000,00	0	0	1	0	0
2	2	1,00	2,00	1	1	1	1	1
3	2	3,00	1,00	1	0	1	1	0
2	2	1,00	1,00	0	1	0	0	1
0	1000	1000,00	1000,00	0	0	0	0	0
0	1000	1000,00	1000,00	0	0	0	0	0
0	1000	1000,00	1000,00	0	0	1	1	0
4	2	1,00	1,00	1	1	1	1	1
0	1000	1000,00	1000,00	0	0	0	0	0
4	1	1,00	1,00	1	1	1	1	1
0	1000	1000,00	1000,00	0	0	1	0	0
0	1000	1000,00	1000,00	0	0	1	0	0
5	2	3,00	1,00	0	0	1	0	0
1	1	1,00	1,00	0	0	1	0	0
0	1000	1000,00	1000,00	1	0	1	0	0
0	1000	1000,00	1000,00	0	0	0	0	0
0	1000	1000,00	1000,00	0	0	0	0	0
0	1000	1000,00	1000,00	0	0	1	0	0
0	1000	1000,00	1000,00	0	0	0	0	0
0	1000	1000,00	1000,00	0	0	0	0	0

Partnerblaı	Partnertrie	Partnermo	Partneruse	Partnerjeoı	Partnerresı	TOT_EMOT	Whodidthiı	Howoldthe
0	0	0	0	0	0	0	99,00	3,00
0	1	0	0	0	1	3	3,00	1,00
0	0	0	0	1	0	1	99,00	1,00
#NULL!	#NULL!	#NULL!	#NULL!	#NULL!	#NULL!	#NULL!	99,00	99,00
0	1	0	1	1	1	7	2,00	1,00
0	0	0	0	0	0	0	1000,00	1000,00
0	0	0	0	1	0	3	1,00	3,00
0	0	0	0	1	1	3	2,00	2,00
0	0	0	0	0	0	1	1,00	1,00
1	0	0	0	1	1	3	1,00	1,00
0	0	0	1	1	#NULL!	#NULL!	2,00	1,00
0	0	1	0	1	0	3	2,00	1,00
0	0	0	0	0	0	0	1000,00	1000,00
0	0	0	0	1	1	2	2,00	1,00
1	1	1	1	0	1	8	2,00	1,00
0	0	0	0	0	0	0	1000,00	1000,00
0	1	0	0	0	0	1	2,00	3,00
1	0	0	0	0	0	#NULL!	3,00	1,00
0	0	0	0	0	0	0	1000,00	1000,00
0	0	0	1	1	1	7	2,00	2,00
0	1	0	0	1	1	5	2,00	1,00
0	0	0	0	0	0	1	1,00	1,00
0	0	0	0	0	0	0	1000,00	1000,00
0	0	0	0	0	0	1	1,00	1,00
0	0	1	0	1	1	4	1,00	1,00
0	1	0	0	0	0	2	1,00	3,00
0	0	0	0	0	0	0	1000,00	1000,00
0	0	0	0	0	0	#NULL!	1000,00	1000,00
0	1	1	0	1	0	4	1,00	3,00
1	0	1	0	1	1	6	1,00	1,00
0	0	#NULL!	0	1	0	#NULL!	99,00	99,00
0	0	0	1	0	0	#NULL!	2,00	1,00
0	0	1	1	1	1	7	2,00	1,00
1	1	1	1	1	1	11	2,00	1,00
0	0	1	1	0	1	3	1,00	1,00
1	1	1	1	0	1	10	2,00	1,00
0	0	0	0	0	0	2	1,00	1,00
0	0	0	0	0	0	0	1000,00	1000,00
0	0	0	0	1	0	1	1,00	1,00
0	0	1	1	1	#NULL!	#NULL!	1,00	1,00
0	0	0	0	0	0	0	1000,00	1000,00
0	0	0	0	1	0	2	1,00	1,00
0	0	0	0	1	1	#NULL!	1,00	2,00
0	0	0	1	1	0	2	99,00	99,00
0	0	0	1	1	0	5	3,00	3,00
0	0	0	0	0	0	0	1000,00	1000,00
1	0	0	0	1	0	4	1,00	1,00
0	0	1	0	1	0	2	1,00	1,00
0	1	1	0	1	0	5	1,00	3,00

0	0	0	0	0	0	4	2,00	3,00
0	0	0	0	0	0	1	99,00	99,00
0	0	0	0	0	0	0	1000,00	1000,00
0	0	0	0	0	0	0	1000,00	1000,00
0	0	0	0	0	0	3	3,00	1,00
0	0	0	0	0	0	0	1000,00	1000,00
1	0	1	0	1	1	9	2,00	1,00
0	0	0	0	1	0	4	1,00	1,00
0	0	1	0	1	0	3	1,00	3,00
0	0	0	0	0	0	0	1000,00	1000,00
0	0	0	0	1	0	1	1,00	1,00
0	1	0	0	0	0	3	1,00	1,00
0	0	0	1	1	0	3	2,00	1,00
0	0	0	1	1	0	4	2,00	3,00
0	0	1	0	0	1	2	3,00	3,00
0	0	0	0	0	0	0	1000,00	1000,00
1	0	0	0	1	0	4	2,00	1,00
0	0	0	0	0	0	0	1000,00	1000,00
0	0	0	0	0	0	0	1000,00	1000,00
0	1	0	0	0	1	4	1,00	1,00
0	0	0	0	1	0	2	99,00	99,00
1	1	0	0	1	1	6	1,00	3,00
0	0	0	0	0	0	0	1000,00	1000,00
0	0	0	0	0	0	0	1000,00	1000,00
0	0	0	0	0	0	0	1000,00	1000,00
0	0	0	0	0	0	0	1000,00	1000,00
0	0	0	0	1	0	1	2,00	1,00
1	0	0	0	0	0	1	99,00	99,00
1	0	0	0	1	1	5	1,00	1,00
0	0	1	1	1	0	3	2,00	1,00
0	0	0	0	1	0	2	3,00	1,00
0	0	0	0	0	0	0	1000,00	1000,00
0	0	0	0	1	0	1	1,00	99,00
0	0	1	1	1	1	9	2,00	3,00
1	0	0	0	1	0	4	1,00	2,00
0	0	0	0	0	0	0	1000,00	1000,00
1	0	1	0	0	0	4	1,00	1,00
0	0	1	0	1	1	6	3,00	1,00
0	0	0	0	1	0	3	2,00	3,00
0	0	1	0	0	0	2	1,00	3,00
0	0	1	0	0	0	1	1,00	1,00
0	0	0	0	0	1	1	3,00	1,00
0	0	0	0	1	0	2	1,00	1,00
0	0	0	0	0	0	0	1000,00	1000,00
0	0	0	0	0	0	2	1,00	1,00
1	1	1	0	1	1	10	2,00	1,00
0	0	0	0	0	0	0	1000,00	1000,00
0	0	0	0	0	0	2	1,00	1,00
0	0	0	0	0	0	0	1000,00	1000,00
1	0	0	0	1	0	4	2,00	1,00

0	0	0	0	0	0	0	1000,00	1000,00
0	0	0	0	0	0	1	2,00	1,00
0	0	0	0	0	0	0	1000,00	1000,00
0	0	0	0	0	0	0	1000,00	1000,00
0	0	1	0	1	0	3	3,00	1,00
0	0	0	0	0	0	0	1000,00	1000,00
0	0	0	0	0	0	0	1000,00	1000,00
0	0	0	0	0	0	0	1000,00	1000,00
0	0	0	0	1	1	3	2,00	1,00
0	0	0	0	0	0	0	1000,00	1000,00
1	0	1	0	0	0	4	3,00	1,00
0	0	0	0	1	0	2	1,00	1,00
0	0	0	0	1	0	1	1,00	3,00
1	0	0	0	1	1	7	1,00	2,00
0	0	0	0	0	1	2	1,00	1,00
0	0	0	0	0	0	0	1000,00	1000,00
0	0	0	0	0	0	1	1,00	1,00
0	0	0	0	0	0	0	1000,00	1000,00
0	0	0	0	0	0	0	1000,00	1000,00
0	0	0	0	0	0	0	1000,00	1000,00
0	0	0	0	0	0	0	1000,00	1000,00
0	0	1	0	0	0	3	3,00	2,00
0	0	0	0	0	0	0	1000,00	1000,00
0	0	0	0	0	0	0	1000,00	1000,00
0	0	0	0	0	0	1	1,00	1,00
0	0	0	0	0	0	0	1000,00	1000,00
1	1	1	0	1	0	8	1,00	1,00
1	1	0	0	1	0	5	1,00	1,00
0	0	0	0	0	0	0	1000,00	1000,00
0	0	0	0	0	0	0	1000,00	1000,00
0	0	0	0	0	0	0	1000,00	1000,00
0	0	0	0	0	0	0	1000,00	1000,00
0	0	0	0	0	0	1	2,00	1,00
0	0	0	0	0	0	0	1000,00	1000,00
0	0	0	0	0	0	0	1000,00	1000,00
0	0	0	0	0	0	0	1000,00	1000,00
0	0	0	0	1	0	3	2,00	3,00
1	1	0	1	0	1	6	2,00	1,00
0	0	0	0	0	0	1	1,00	1,00
0	0	0	0	0	0	0	1000,00	1000,00
1	0	0	0	0	0	2	3,00	3,00
0	0	0	0	0	0	0	1000,00	1000,00
0	0	0	0	0	0	0	1000,00	1000,00
0	0	0	0	0	0	0	1000,00	1000,00
0	0	0	1	0	0	3	1,00	1,00
0	0	0	0	0	0	2	1,00	1,00
1	1	1	0	1	0	5	1,00	2,00
0	0	0	0	0	0	0	1000,00	1000,00
1	0	1	0	1	0	4	2,00	1,00
0	0	0	0	0	0	0	1000,00	1000,00

0	0	0	0	0	1	3	1,00	1,00
0	0	0	0	0	0	0	1000,00	1000,00
0	0	1	0	0	0	1	1,00	1,00
0	0	0	0	0	0	0	1000,00	1000,00
0	0	1	0	0	0	2	1,00	1,00
0	0	0	0	1	0	2	2,00	1,00
0	0	0	0	0	0	0	1000,00	1000,00
0	0	0	0	1	1	4	2,00	1,00
0	0	0	0	0	0	0	1000,00	1000,00
0	0	0	0	1	0	2	1,00	1,00
0	0	0	1	0	1	4	2,00	1,00
0	0	0	0	0	0	0	1000,00	1000,00
0	0	0	0	0	0	0	1000,00	1000,00
0	0	0	0	0	0	4	3,00	1,00
0	0	0	0	0	0	0	1000,00	1000,00
0	0	0	0	0	0	0	1000,00	1000,00
0	0	0	0	0	0	0	1000,00	1000,00
0	0	0	0	0	0	0	1000,00	1000,00
0	0	0	0	0	0	0	1000,00	1000,00
0	0	0	0	0	0	0	1000,00	1000,00
0	0	0	0	0	0	0	1000,00	1000,00
0	0	0	0	0	0	0	1000,00	1000,00
0	0	0	0	1	0	2	1,00	1,00
0	0	0	0	1	0	2	2,00	3,00
0	0	0	0	0	0	1	2,00	1,00
0	0	0	0	0	0	0	1000,00	1000,00
0	0	0	0	0	0	0	1000,00	1000,00
0	0	0	0	0	0	0	1000,00	1000,00
0	1	0	0	1	0	4	1,00	1,00
0	0	0	0	0	0	0	1000,00	1000,00
0	0	0	0	0	0	1	1,00	3,00
0	0	0	0	1	0	2	1,00	3,00
0	0	0	0	0	0	0	1000,00	1000,00
1	1	0	0	1	0	6	2,00	3,00
0	0	0	0	0	0	0	1000,00	1000,00
1	1	0	1	0	0	3	1,00	1,00
1	0	1	1	1	0	4	2,00	1,00
0	0	0	0	0	0	0	1000,00	1000,00
0	0	0	0	0	0	0	1000,00	1000,00
0	1	1	0	1	1	4	2,00	1,00
0	0	0	1	0	0	1	3,00	1,00
0	0	0	0	0	0	1	1,00	1,00
0	0	0	0	0	0	1	1,00	1,00
0	0	0	0	0	1	2	2,00	1,00
0	0	0	0	0	0	0	1000,00	1000,00
0	0	0	0	0	0	0	1000,00	1000,00
0	0	0	0	0	0	0	1000,00	1000,00
0	0	0	0	0	0	0	1000,00	1000,00
0	0	0	0	0	0	0	1000,00	1000,00
0	0	0	0	0	0	0	1000,00	1000,00

0	0	0	0	0	0	0	1000,00	1000,00
0	0	0	0	0	0	0	1000,00	1000,00
0	0	0	0	0	0	0	1000,00	1000,00
0	0	0	0	0	0	0	1000,00	1000,00
0	0	0	0	0	0	1	1,00	3,00
0	0	0	0	1	1	3	3,00	1,00
0	0	0	0	0	0	1	1,00	1,00
0	0	1	0	1	0	3	1,00	1,00
0	0	0	0	0	0	0	1000,00	1000,00
0	0	0	0	0	0	0	1000,00	1000,00
0	0	0	0	0	1	2	1,00	1,00
0	0	0	0	0	0	0	1000,00	1000,00
0	0	0	0	0	0	0	1000,00	1000,00
0	0	0	0	0	0	0	1000,00	1000,00
0	0	0	0	0	0	0	1000,00	1000,00
0	0	0	0	0	0	0	1000,00	1000,00
0	0	0	0	0	0	0	1000,00	1000,00
1	1	0	1	0	1	7	1,00	1,00
0	0	0	0	0	0	0	1000,00	1000,00
1	0	0	0	0	0	3	1,00	1,00
0	0	0	0	0	0	0	1000,00	1000,00
0	0	0	1	0	0	1	1,00	3,00
0	0	0	0	0	0	1	2,00	1,00
0	1	0	0	0	1	6	3,00	2,00
0	0	0	1	1	1	3	2,00	2,00
0	0	0	0	0	0	1	2,00	1,00
0	0	0	0	0	0	0	1000,00	1000,00
0	0	0	0	0	0	3	2,00	1,00
1	1	0	0	1	1	5	2,00	1,00
0	0	0	0	0	0	0	1000,00	1000,00
0	0	0	0	0	0	1	1,00	1,00
0	0	0	0	0	0	0	1000,00	1000,00
0	1	0	0	1	0	6	2,00	1,00
0	0	0	0	0	1	3	1,00	3,00
1	1	0	0	0	1	7	2,00	2,00
0	1	0	0	1	0	5	2,00	1,00
0	0	0	0	1	1	4	1,00	3,00
1	0	0	0	1	1	5	2,00	1,00
1	1	0	0	0	0	4	2,00	1,00
1	1	0	1	1	1	6	1,00	1,00
0	0	1	0	1	1	4	1,00	1,00
#NULL!	0	0	0	0	0	#NULL!	1,00	1,00
0	1	0	1	0	0	3	1,00	1,00
0	0	0	0	1	0	1	1,00	1,00
1	0	0	0	0	0	1	1000,00	1000,00
0	0	0	0	0	0	1	1000,00	1000,00
0	0	0	1	0	0	2	1,00	3,00
0	0	0	0	0	0	0	1000,00	1000,00
0	1	0	1	1	0	7	2,00	1,00
0	1	0	0	1	1	6	3,00	1,00

0	0	0	1	0	1	3	3,00	1,00
0	0	0	0	1	0	2	1,00	1,00
0	0	0	0	0	0	0	1000,00	1000,00
0	0	0	0	0	0	0	1000,00	1000,00
0	0	0	0	0	0	0	1000,00	1000,00
0	0	0	0	0	0	1	1,00	1,00
0	0	0	0	0	1	3	2,00	1,00
0	0	0	0	0	0	0	1000,00	1000,00
1	1	0	0	1	1	8	3,00	1,00
0	0	0	0	0	0	1	1,00	1,00
0	0	1	0	1	1	4	1,00	1,00
1	0	1	0	1	0	4	2,00	3,00
0	0	0	0	0	0	0	1000,00	1000,00
0	0	0	0	0	0	0	1000,00	1000,00
1	1	1	0	1	1	9	2,00	3,00
0	0	0	0	0	0	3	2,00	3,00
0	1	0	1	0	0	3	1,00	1,00
0	0	0	0	0	0	0	1000,00	1000,00
0	0	0	0	0	0	0	1000,00	1000,00
0	0	0	0	1	1	6	2,00	1,00
0	0	0	0	1	0	1	2,00	1,00
0	0	0	0	0	0	0	1000,00	1000,00
0	1	1	1	1	0	6	2,00	1,00
0	0	0	0	0	0	0	1000,00	1000,00
0	0	0	0	0	0	0	1000,00	1000,00
0	0	0	0	0	0	0	1000,00	1000,00
0	0	0	0	0	0	0	1000,00	1000,00
0	0	0	0	0	0	0	1000,00	1000,00
0	0	0	0	0	0	0	1000,00	1000,00
0	1	1	0	1	0	8	2,00	1,00
0	0	0	0	0	0	0	1000,00	1000,00
0	0	0	0	0	0	0	1000,00	1000,00
0	0	0	0	0	0	0	1000,00	1000,00
0	0	0	0	0	0	0	1000,00	1000,00
0	0	0	0	1	1	2	2,00	1,00
0	0	0	0	0	0	1	1000,00	1000,00
0	0	0	0	0	0	0	1000,00	1000,00
0	0	0	0	1	1	2	1,00	1,00
1	1	0	0	1	1	7	1,00	1,00
0	0	0	0	0	0	0	1000,00	1000,00
0	0	1	0	1	1	4	3,00	1,00
0	0	0	0	1	1	6	3,00	2,00
0	0	0	0	0	0	0	1000,00	1000,00
0	0	0	0	0	0	0	1000,00	1000,00
0	0	0	0	1	1	2	3,00	3,00
0	0	0	0	0	0	0	1000,00	1000,00
0	0	1	0	1	1	3	1,00	1,00
0	0	0	0	1	0	2	1,00	1,00
0	0	0	0	0	0	0	1000,00	1000,00
0	0	0	0	0	0	0	1000,00	1000,00

0	0	0	0	0	0	0	1000,00	1000,00
1	0	0	0	1	0	3	3,00	1,00
0	0	0	0	0	0	0	1000,00	1000,00
0	0	0	0	1	0	2	2,00	3,00
0	0	0	1	1	1	4	3,00	3,00
0	0	0	0	0	0	0	1000,00	1000,00
0	0	0	0	0	0	0	1000,00	1000,00
0	0	0	0	0	0	0	1000,00	1000,00
0	0	0	0	0	0	1	2,00	3,00
0	0	0	0	0	0	0	1000,00	1000,00
0	0	0	0	0	0	0	1000,00	1000,00
0	0	0	0	0	0	0	1000,00	1000,00
0	0	0	0	0	0	0	1000,00	1000,00
0	0	0	0	0	0	0	1000,00	1000,00
1	0	0	0	0	1	3	2,00	3,00
0	0	0	0	0	0	0	1000,00	1000,00
0	0	0	1	0	0	1	1,00	2,00
0	0	0	0	0	0	0	1000,00	1000,00
0	0	0	0	0	0	0	1000,00	1000,00
0	0	0	0	0	0	0	1000,00	1000,00
0	0	1	0	1	0	3	1,00	1,00
0	0	0	0	0	0	0	1000,00	1000,00
0	0	0	0	0	0	0	1000,00	1000,00
0	0	0	0	0	0	0	1000,00	1000,00
0	0	0	0	0	0	0	1000,00	1000,00
0	0	0	0	0	0	0	1000,00	1000,00
0	0	0	0	1	0	2	1,00	3,00
0	0	0	0	1	0	2	1,00	1,00
1	0	0	0	0	0	3	2,00	2,00
0	0	1	0	1	0	3	1,00	1,00
0	0	0	0	0	0	2	2,00	3,00
0	0	0	0	1	0	2	2,00	3,00
0	0	0	0	0	0	0	1000,00	1000,00
0	0	0	0	0	0	0	1000,00	1000,00
0	0	0	0	0	0	0	1000,00	1000,00
0	0	0	0	0	0	0	1000,00	1000,00
0	0	0	0	0	0	0	1000,00	1000,00
0	0	0	0	0	0	0	1000,00	1000,00
1	1	0	1	0	0	4	2,00	3,00
0	0	0	0	0	0	0	1000,00	1000,00
0	0	1	0	0	0	1	2,00	3,00
0	0	0	0	0	0	0	1000,00	1000,00
0	0	0	0	0	0	1	1,00	1,00
#NULL!	#NULL!	#NULL!	#NULL!	#NULL!	#NULL!	#NULL!	1000,00	1000,00
0	0	0	0	0	0	0	1000,00	1000,00
0	0	0	0	0	0	0	1000,00	1000,00
0	0	0	0	0	0	0	1000,00	1000,00
0	0	0	0	0	0	0	1000,00	1000,00
0	0	0	0	1	0	1	2,00	3,00
1	1	1	0	1	0	5	1,00	1,00

0	0	0	0	0	0	0	1000,00	1000,00
0	0	0	0	0	0	0	1000,00	1000,00
0	0	0	0	0	0	0	1000,00	1000,00
0	0	1	0	1	0	5	1,00	1,00
0	0	0	0	0	0	0	1000,00	1000,00
0	0	0	1	1	0	5	1,00	1,00
0	0	0	0	0	0	0	1000,00	1000,00
0	0	0	0	0	0	0	1000,00	1000,00
0	0	0	0	0	0	0	1000,00	1000,00
0	0	0	0	0	0	0	1000,00	1000,00
0	0	0	0	0	0	0	1000,00	1000,00
0	0	0	0	0	0	0	1000,00	1000,00
0	0	0	0	0	0	0	1000,00	1000,00
0	1	0	0	1	0	4	1,00	3,00
0	0	0	0	1	0	4	1,00	1,00
0	0	0	0	0	0	0	1000,00	1000,00
0	0	0	0	0	0	0	1000,00	1000,00
0	0	1	0	1	1	4	1,00	3,00
1	1	1	0	1	0	8	2,00	3,00
0	0	0	0	1	1	2	2,00	1,00
0	0	0	0	1	0	2	2,00	3,00
0	0	0	0	0	0	0	1000,00	1000,00
0	0	0	0	0	0	0	1000,00	1000,00
0	0	1	0	1	0	3	3,00	1,00
1	1	0	0	0	1	6	2,00	1,00
0	1	0	0	1	0	3	1,00	1,00
1	0	0	0	1	0	3	3,00	1,00
0	0	0	0	0	0	0	1000,00	1000,00
0	0	0	0	1	0	2	2,00	1,00
0	0	0	0	0	1	4	1,00	1,00
0	0	0	0	0	0	0	1000,00	1000,00
0	0	0	0	1	0	1	1,00	2,00
1	1	1	0	1	1	8	1,00	1,00
0	0	0	1	0	0	3	2,00	1,00
0	0	0	0	1	0	3	1,00	1,00
0	0	0	0	0	0	0	1000,00	1000,00
0	0	0	0	1	0	1	1,00	1,00
0	0	0	0	0	0	0	1000,00	1000,00
1	1	1	0	0	0	3	2,00	1,00
0	1	0	0	1	1	5	2,00	1,00
1	1	1	0	0	1	9	1,00	1,00
0	0	0	0	1	0	2	1,00	1,00
0	1	0	0	1	0	4	2,00	3,00
0	0	0	0	0	0	0	1000,00	1000,00
0	0	0	0	0	0	0	1000,00	1000,00
0	0	0	0	0	0	0	1000,00	1000,00
0	0	0	0	0	0	0	1000,00	1000,00
1	1	0	0	1	1	7	1,00	1,00
0	0	0	0	0	0	2	1,00	1,00
0	0	0	1	0	0	1	2,00	1,00

1	0	0	0	1	1	3	1,00	3,00
1	1	0	0	1	1	6	1,00	1,00
0	0	0	0	0	0	3	1,00	3,00
0	0	0	0	0	0	0	1000,00	1000,00
0	0	0	0	0	0	0	1000,00	1000,00
0	0	0	0	0	0	0	1000,00	1000,00
0	0	0	0	0	0	0	1000,00	1000,00
0	0	1	0	0	0	2	1,00	1,00
0	0	0	0	0	0	0	1000,00	1000,00
0	0	0	0	0	1	1	3,00	3,00
0	0	0	0	0	0	3	1,00	1,00
0	0	0	0	1	1	2	1,00	1,00
0	0	0	0	1	0	2	2,00	3,00
1	1	1	1	1	1	11	2,00	1,00
1	1	0	0	0	0	5	2,00	3,00
0	1	0	1	1	0	5	2,00	1,00
0	0	0	0	0	0	0	1000,00	1000,00
0	0	0	0	0	0	0	1000,00	1000,00
0	0	1	0	0	1	4	2,00	1,00
1	0	0	0	0	0	6	2,00	1,00
0	0	0	0	0	0	0	1000,00	1000,00
1	1	0	0	1	0	8	1,00	1,00
0	0	0	0	0	0	1	1,00	1,00
0	0	0	0	1	0	2	1,00	3,00
0	0	0	0	1	0	2	1,00	3,00
0	0	0	0	0	0	1	1000,00	1000,00
0	0	0	1	1	0	4	2,00	1,00
0	0	0	0	0	0	0	1000,00	1000,00
0	0	0	0	1	0	1	2,00	1,00
0	0	0	0	0	0	1	1,00	1,00
0	0	0	0	0	0	0	1000,00	1000,00
0	0	0	0	0	0	0	1000,00	1000,00

istuisactsha	filter_$	PHYSICAL_	SEX_PREVA	EMOTIONA	ABUSE	ABUSE_PRI	AAAAA	BBBB
2,00	1	1	1	0	2,00	1	1,00	0,00
2,00	1	0	0	1	1,00	1	1,00	0,00
2,00	1	0	1	1	2,00	1	1,00	0,00
99,00	1	0	#NULL!	#NULL!	#NULL!	#NULL!	0,00	1,00
1,00	1	1	1	1	3,00	1	0,00	1,00
1000,00	1	0	0	0	0,00	0	1,00	0,00
1,00	1	0	1	1	2,00	1	1,00	0,00
1,00	1	0	1	1	2,00	1	0,00	1,00
1,00	1	0	1	1	2,00	1	1,00	0,00
1,00	1	1	1	1	3,00	1	1,00	0,00
1,00	1	0	0	#NULL!	#NULL!	#NULL!	1,00	0,00
2,00	1	0	1	1	2,00	1	1,00	0,00
1000,00	1	1	1	0	2,00	1	1,00	0,00
2,00	1	1	1	1	3,00	1	1,00	0,00
2,00	1	1	1	1	3,00	1	0,00	1,00
1000,00	1	0	0	0	0,00	0	1,00	0,00
1,00	1	0	1	1	2,00	1	1,00	0,00
1,00	1	0	1	#NULL!	#NULL!	#NULL!	1,00	0,00
1000,00	1	1	0	0	1,00	1	1,00	0,00
1,00	1	1	1	1	3,00	1	1,00	0,00
1,00	1	1	1	1	3,00	1	0,00	1,00
1,00	1	1	1	1	3,00	1	1,00	0,00
1000,00	1	0	1	0	1,00	1	0,00	1,00
1,00	1	0	1	1	2,00	1	0,00	1,00
2,00	1	0	1	1	2,00	1	0,00	1,00
1,00	1	#NULL!	1	1	#NULL!	#NULL!	1,00	0,00
1000,00	1	0	0	0	0,00	0	1,00	0,00
1000,00	1	0	0	#NULL!	#NULL!	#NULL!	1,00	0,00
2,00	1	#NULL!	1	1	#NULL!	#NULL!	1,00	0,00
1,00	1	0	1	1	2,00	1	0,00	1,00
2,00	1	0	0	#NULL!	#NULL!	#NULL!	1,00	0,00
1,00	1	0	0	#NULL!	#NULL!	#NULL!	1,00	0,00
1,00	1	#NULL!	#NULL!	1	#NULL!	#NULL!	0,00	1,00
2,00	1	1	1	1	3,00	1	0,00	1,00
1,00	1	0	1	1	2,00	1	1,00	0,00
2,00	1	1	1	1	3,00	1	1,00	0,00
2,00	1	1	0	1	2,00	1	0,00	1,00
1000,00	1	#NULL!	#NULL!	0	#NULL!	#NULL!	1,00	0,00
1,00	1	0	1	1	2,00	1	0,00	0,00
1,00	1	1	1	#NULL!	#NULL!	#NULL!	0,00	1,00
1000,00	1	0	0	0	0,00	0	0,00	1,00
1,00	1	0	1	1	2,00	1	1,00	0,00
1,00	1	1	#NULL!	#NULL!	#NULL!	#NULL!	1,00	0,00
2,00	1	0	0	1	1,00	1	1,00	0,00
1,00	1	0	1	1	2,00	1	0,00	1,00
1000,00	1	0	0	0	0,00	0	1,00	0,00
1,00	1	1	1	1	3,00	1	1,00	0,00
1,00	1	0	1	1	2,00	1	1,00	0,00
1,00	1	0	0	1	1,00	1	1,00	0,00

2,00	1	1	1	1	3,00	1	1,00	0,00
99,00	1	0	0	1	1,00	1	1,00	0,00
1000,00	1	0	1	0	1,00	1	1,00	0,00
1000,00	1	0	0	0	0,00	0	0,00	1,00
2,00	1	0	0	1	1,00	1	1,00	0,00
1000,00	1	1	1	0	2,00	1	1,00	0,00
2,00	1	1	0	1	2,00	1	1,00	0,00
1,00	1	0	0	1	1,00	1	1,00	0,00
1,00	1	1	0	1	2,00	1	1,00	0,00
1000,00	1	0	0	0	0,00	0	1,00	0,00
1,00	1	0	0	1	1,00	1	1,00	0,00
2,00	1	0	0	1	1,00	1	0,00	0,00
2,00	1	0	0	1	1,00	1	1,00	0,00
1,00	1	1	1	1	3,00	1	1,00	0,00
1,00	1	0	1	1	2,00	1	1,00	0,00
1000,00	1	1	0	0	1,00	1	1,00	0,00
1,00	1	1	1	1	3,00	1	0,00	1,00
1000,00	1	0	0	0	0,00	0	1,00	0,00
1000,00	1	0	0	0	0,00	0	1,00	0,00
2,00	1	1	1	1	3,00	1	0,00	0,00
99,00	1	0	0	1	1,00	1	1,00	0,00
2,00	1	1	1	1	3,00	1	0,00	1,00
1000,00	1	0	0	0	0,00	0	1,00	0,00
1000,00	1	1	1	0	2,00	1	0,00	1,00
1000,00	1	0	0	0	0,00	0	1,00	0,00
1000,00	1	0	0	0	0,00	0	0,00	1,00
2,00	1	1	0	1	2,00	1	1,00	0,00
2,00	1	0	0	1	1,00	1	1,00	0,00
1,00	1	1	1	1	3,00	1	1,00	0,00
1,00	1	1	1	1	3,00	1	0,00	1,00
1,00	1	0	1	1	2,00	1	1,00	0,00
1000,00	1	0	0	0	0,00	0	1,00	0,00
1,00	1	0	0	1	1,00	1	0,00	1,00
1,00	1	1	1	1	3,00	1	0,00	1,00
1,00	1	1	0	1	2,00	1	1,00	0,00
1000,00	1	0	0	0	0,00	0	1,00	0,00
1,00	1	1	0	1	2,00	1	1,00	0,00
1,00	1	0	0	1	1,00	1	1,00	0,00
1,00	1	0	1	1	2,00	1	1,00	0,00
2,00	1	1	0	1	2,00	1	0,00	1,00
1,00	1	0	0	1	1,00	1	1,00	0,00
2,00	1	0	0	1	1,00	1	1,00	0,00
1,00	1	0	0	1	1,00	1	0,00	1,00
1000,00	1	0	0	0	0,00	0	0,00	1,00
2,00	1	0	0	1	1,00	1	1,00	0,00
2,00	1	1	0	1	2,00	1	1,00	0,00
1000,00	1	0	0	0	0,00	0	1,00	0,00
1,00	1	1	0	1	2,00	1	1,00	0,00
1000,00	1	0	0	0	0,00	0	1,00	0,00
1,00	1	1	0	1	2,00	1	1,00	0,00

1000,00	1	0	0	0	0,00	0	1,00	0,00
2,00	1	0	0	1	1,00	1	1,00	0,00
1000,00	1	0	1	0	1,00	1	0,00	0,00
1000,00	1	0	0	0	0,00	0	1,00	0,00
1,00	1	0	0	1	1,00	1	1,00	0,00
1000,00	1	0	0	0	0,00	0	1,00	0,00
1000,00	1	0	0	0	0,00	0	1,00	0,00
1000,00	1	0	0	0	0,00	0	1,00	0,00
1,00	1	0	0	1	1,00	1	1,00	0,00
1000,00	1	0	0	0	0,00	0	0,00	1,00
1,00	1	0	0	1	1,00	1	1,00	0,00
2,00	1	0	0	1	1,00	1	1,00	0,00
1,00	1	0	0	1	1,00	1	0,00	1,00
1,00	1	1	1	1	3,00	1	1,00	0,00
1,00	1	0	1	1	2,00	1	0,00	1,00
1000,00	1	0	0	0	0,00	0	1,00	0,00
2,00	1	0	0	1	1,00	1	1,00	0,00
1000,00	1	0	0	0	0,00	0	1,00	0,00
1000,00	1	0	0	0	0,00	0	0,00	1,00
1000,00	1	0	0	0	0,00	0	1,00	0,00
1000,00	1	0	0	0	0,00	0	1,00	0,00
2,00	1	0	0	1	1,00	1	0,00	1,00
1000,00	1	0	0	0	0,00	0	1,00	0,00
1000,00	1	0	0	0	0,00	0	1,00	0,00
2,00	1	1	0	1	2,00	1	0,00	1,00
1000,00	1	0	0	0	0,00	0	0,00	1,00
1,00	1	1	1	1	3,00	1	0,00	1,00
1,00	1	1	1	1	3,00	1	0,00	0,00
1000,00	1	0	0	0	0,00	0	1,00	0,00
1000,00	1	0	0	0	0,00	0	1,00	0,00
1000,00	1	0	1	0	1,00	1	1,00	0,00
1000,00	1	1	0	0	1,00	1	1,00	0,00
2,00	1	0	1	1	2,00	1	1,00	0,00
1000,00	1	0	0	0	0,00	0	1,00	0,00
1000,00	1	0	0	0	0,00	0	1,00	0,00
1000,00	1	0	1	0	1,00	1	1,00	0,00
2,00	1	1	0	1	2,00	1	1,00	0,00
2,00	1	0	0	1	1,00	1	0,00	0,00
2,00	1	0	0	1	1,00	1	1,00	0,00
1000,00	1	0	0	0	0,00	0	1,00	0,00
2,00	1	0	1	1	2,00	1	1,00	0,00
1000,00	1	0	0	0	0,00	0	1,00	0,00
1000,00	1	0	1	0	1,00	1	1,00	0,00
1000,00	1	0	0	0	0,00	0	1,00	0,00
1,00	1	0	1	1	2,00	1	1,00	0,00
1,00	1	1	0	1	2,00	1	1,00	0,00
1,00	1	0	1	1	2,00	1	0,00	1,00
1000,00	1	0	0	0	0,00	0	1,00	0,00
1,00	1	1	1	1	3,00	1	0,00	1,00
1000,00	1	0	0	0	0,00	0	1,00	0,00

1,00	1	0	1	1	2,00	1	0,00	1,00
1000,00	1	0	0	0	0,00	0	0,00	1,00
1,00	1	1	1	1	3,00	1	1,00	0,00
1000,00	1	0	0	0	0,00	0	1,00	0,00
1,00	1	0	0	1	1,00	1	1,00	0,00
1,00	1	0	0	1	1,00	1	0,00	0,00
1000,00	1	0	0	0	0,00	0	0,00	1,00
1,00	1	0	1	1	2,00	1	0,00	1,00
1000,00	1	0	0	0	0,00	0	1,00	0,00
2,00	1	0	0	1	1,00	1	0,00	1,00
1,00	1	1	1	1	3,00	1	1,00	0,00
1000,00	1	0	0	0	0,00	0	0,00	1,00
1000,00	1	0	0	0	0,00	0	1,00	0,00
1,00	1	0	0	1	1,00	1	1,00	0,00
1000,00	1	0	0	0	0,00	0	1,00	0,00
1000,00	1	0	1	0	1,00	1	0,00	1,00
1000,00	1	0	0	0	0,00	0	1,00	0,00
1000,00	1	0	0	0	0,00	0	0,00	0,00
1000,00	1	0	0	0	0,00	0	1,00	0,00
1000,00	1	0	0	0	0,00	0	1,00	0,00
1000,00	1	0	0	0	0,00	0	1,00	0,00
1000,00	1	0	0	0	0,00	0	1,00	0,00
2,00	1	1	0	1	2,00	1	0,00	1,00
2,00	1	1	0	1	2,00	1	0,00	1,00
2,00	1	0	0	1	1,00	1	0,00	1,00
1000,00	1	0	0	0	0,00	0	0,00	0,00
1000,00	1	0	0	0	0,00	0	0,00	1,00
1000,00	1	0	0	0	0,00	0	1,00	0,00
1,00	1	1	1	1	3,00	1	0,00	1,00
1000,00	1	0	0	0	0,00	0	1,00	0,00
2,00	1	0	0	1	1,00	1	1,00	0,00
2,00	1	0	0	1	1,00	1	1,00	0,00
1000,00	1	0	0	0	0,00	0	1,00	0,00
2,00	1	1	1	1	3,00	1	1,00	0,00
1000,00	1	0	0	0	0,00	0	1,00	0,00
1,00	1	0	0	1	1,00	1	1,00	0,00
1,00	1	1	0	1	2,00	1	1,00	0,00
1000,00	1	0	0	0	0,00	0	0,00	1,00
1000,00	1	0	0	0	0,00	0	1,00	0,00
1,00	1	0	1	1	2,00	1	1,00	0,00
1,00	1	0	0	1	1,00	1	1,00	0,00
2,00	1	0	0	1	1,00	1	1,00	0,00
1,00	1	0	0	1	1,00	1	1,00	0,00
1,00	1	0	0	1	1,00	1	0,00	1,00
1000,00	1	1	0	0	1,00	1	1,00	0,00
1000,00	1	0	0	0	0,00	0	0,00	1,00
1000,00	1	0	0	0	0,00	0	1,00	0,00
1000,00	1	0	0	0	0,00	0	1,00	0,00
1000,00	1	0	#NULL!	0	#NULL!	#NULL!	1,00	0,00
1000,00	1	0	0	0	0,00	0	1,00	0,00

1000,00	1	0	0	0	0,00	0	1,00	0,00
1000,00	1	0	0	0	0,00	0	1,00	0,00
1000,00	1	0	0	0	0,00	0	0,00	1,00
1000,00	1	0	0	0	0,00	0	1,00	0,00
1,00	1	0	0	1	1,00	1	1,00	0,00
1,00	1	0	0	1	1,00	1	1,00	0,00
1,00	1	0	0	1	1,00	1	1,00	0,00
1,00	1	1	0	1	2,00	1	1,00	0,00
1000,00	1	0	0	0	0,00	0	1,00	0,00
1000,00	1	0	0	0	0,00	0	1,00	0,00
1,00	1	0	0	1	1,00	1	1,00	0,00
1000,00	1	0	0	0	0,00	0	0,00	1,00
1000,00	1	0	0	0	0,00	0	0,00	0,00
1000,00	1	0	0	0	0,00	0	1,00	0,00
1000,00	1	0	0	0	0,00	0	1,00	0,00
1000,00	1	0	1	0	1,00	1	1,00	0,00
1000,00	1	0	0	0	0,00	0	0,00	0,00
1,00	1	1	1	1	3,00	1	1,00	0,00
1000,00	1	0	0	0	0,00	0	1,00	0,00
1,00	1	0	0	1	1,00	1	1,00	0,00
1000,00	1	0	0	0	0,00	0	0,00	0,00
1,00	1	0	0	1	1,00	1	0,00	1,00
1,00	1	0	0	1	1,00	1	1,00	0,00
1,00	1	1	0	1	2,00	1	1,00	0,00
2,00	1	0	1	1	2,00	1	0,00	1,00
1,00	1	0	0	1	1,00	1	0,00	1,00
1000,00	1	0	0	0	0,00	0	1,00	0,00
1,00	1	1	1	1	3,00	1	1,00	0,00
1,00	1	1	0	1	2,00	1	1,00	0,00
1000,00	1	0	0	0	0,00	0	0,00	0,00
1,00	1	0	0	1	1,00	1	1,00	0,00
1000,00	1	0	0	0	0,00	0	0,00	1,00
1,00	1	0	1	1	2,00	1	1,00	0,00
1,00	1	1	0	1	2,00	1	1,00	0,00
1,00	1	1	1	1	3,00	1	1,00	0,00
1,00	1	0	1	1	2,00	1	0,00	1,00
1,00	1	0	0	1	1,00	1	1,00	0,00
2,00	1	0	0	1	1,00	1	1,00	0,00
2,00	1	1	1	1	3,00	1	1,00	0,00
1,00	1	0	0	1	1,00	1	1,00	0,00
1,00	1	1	1	1	3,00	1	1,00	0,00
1,00	1	1	1	#NULL!	#NULL!	#NULL!	1,00	0,00
1,00	1	0	1	1	2,00	1	1,00	0,00
1,00	1	0	0	1	1,00	1	1,00	0,00
1000,00	1	0	0	1	1,00	1	1,00	0,00
1000,00	1	0	0	1	1,00	1	1,00	0,00
1,00	1	0	0	1	1,00	1	1,00	0,00
1000,00	1	0	0	0	0,00	0	1,00	0,00
1,00	1	0	1	1	2,00	1	1,00	0,00
1,00	1	0	1	1	2,00	1	1,00	0,00

1,00	1	0	0	1	1,00	1	1,00	0,00
1,00	1	0	0	1	1,00	1	0,00	1,00
1000,00	1	0	0	0	0,00	0	1,00	0,00
1000,00	1	0	0	0	0,00	0	0,00	1,00
1000,00	1	0	0	0	0,00	0	1,00	0,00
1,00	1	0	1	1	2,00	1	0,00	1,00
1,00	1	0	1	1	2,00	1	0,00	1,00
1000,00	1	0	1	0	1,00	1	1,00	0,00
1,00	1	0	0	1	1,00	1	0,00	1,00
1,00	1	0	1	1	2,00	1	1,00	0,00
1,00	1	1	0	1	2,00	1	1,00	0,00
2,00	1	0	0	1	1,00	1	1,00	0,00
1000,00	1	0	0	0	0,00	0	1,00	0,00
1000,00	1	1	1	0	2,00	1	0,00	1,00
1,00	1	0	1	1	2,00	1	1,00	0,00
2,00	1	0	1	1	2,00	1	1,00	0,00
1,00	1	0	0	1	1,00	1	1,00	0,00
1000,00	1	0	0	0	0,00	0	1,00	0,00
1000,00	1	0	0	0	0,00	0	0,00	1,00
2,00	1	0	1	1	2,00	1	1,00	0,00
1,00	1	0	0	1	1,00	1	1,00	0,00
1000,00	1	0	0	0	0,00	0	1,00	0,00
2,00	1	1	1	1	3,00	1	1,00	0,00
1000,00	1	0	0	0	0,00	0	1,00	0,00
1000,00	1	0	0	0	0,00	0	1,00	0,00
1000,00	1	0	0	0	0,00	0	0,00	0,00
1000,00	1	0	0	0	0,00	0	1,00	0,00
1000,00	1	0	0	0	0,00	0	1,00	0,00
1000,00	1	0	0	0	0,00	0	1,00	0,00
1,00	1	1	1	1	3,00	1	1,00	0,00
1000,00	1	0	0	0	0,00	0	1,00	0,00
1000,00	1	0	0	0	0,00	0	1,00	0,00
1000,00	1	0	0	0	0,00	0	1,00	0,00
1000,00	1	0	0	0	0,00	0	1,00	0,00
1,00	1	0	0	1	1,00	1	1,00	0,00
1000,00	1	1	0	1	2,00	1	1,00	0,00
1000,00	1	0	0	0	0,00	0	0,00	0,00
1,00	1	0	0	1	1,00	1	1,00	0,00
1,00	1	1	1	1	3,00	1	1,00	0,00
1000,00	1	0	0	0	0,00	0	1,00	0,00
1,00	1	0	0	1	1,00	1	1,00	0,00
2,00	1	0	0	1	1,00	1	1,00	0,00
1000,00	1	0	0	0	0,00	0	1,00	0,00
1000,00	1	0	0	0	0,00	0	1,00	0,00
1,00	1	0	0	1	1,00	1	1,00	0,00
1000,00	1	0	0	0	0,00	0	1,00	0,00
1,00	1	1	0	1	2,00	1	1,00	0,00
1,00	1	0	0	1	1,00	1	1,00	0,00
1000,00	1	0	0	0	0,00	0	1,00	0,00
1000,00	1	0	0	0	0,00	0	1,00	0,00

1000,00	1	0	0	0	0,00	0	1,00	0,00
1,00	1	0	1	1	2,00	1	1,00	0,00
1000,00	1	1	0	0	1,00	1	1,00	0,00
2,00	1	#NULL!	0	1	#NULL!	#NULL!	0,00	0,00
1,00	1	0	0	1	1,00	1	0,00	1,00
1000,00	1	0	0	0	0,00	0	1,00	0,00
1000,00	1	0	0	0	0,00	0	0,00	1,00
1000,00	1	1	0	0	1,00	1	1,00	0,00
2,00	1	0	0	1	1,00	1	1,00	0,00
1000,00	1	0	0	0	0,00	0	1,00	0,00
1000,00	1	0	0	0	0,00	0	1,00	0,00
1000,00	1	0	0	0	0,00	0	1,00	0,00
1000,00	1	0	0	0	0,00	0	0,00	0,00
1000,00	1	0	0	0	0,00	0	0,00	0,00
2,00	1	0	0	1	1,00	1	1,00	0,00
1000,00	1	0	0	0	0,00	0	1,00	0,00
1,00	1	0	1	1	2,00	1	0,00	1,00
1000,00	1	0	0	0	0,00	0	1,00	0,00
1000,00	1	0	0	0	0,00	0	1,00	0,00
1000,00	1	0	0	0	0,00	0	0,00	1,00
1,00	1	1	0	1	2,00	1	1,00	0,00
1000,00	1	0	0	0	0,00	0	0,00	1,00
1000,00	1	0	0	0	0,00	0	0,00	0,00
1000,00	1	0	0	0	0,00	0	1,00	0,00
1000,00	1	0	0	0	0,00	0	1,00	0,00
1000,00	1	0	0	0	0,00	0	0,00	1,00
1,00	1	#NULL!	0	1	#NULL!	#NULL!	1,00	0,00
1,00	1	0	0	1	1,00	1	0,00	1,00
1,00	1	1	0	1	2,00	1	0,00	1,00
1,00	1	1	0	1	2,00	1	0,00	1,00
1,00	1	0	0	1	1,00	1	1,00	0,00
2,00	1	1	0	1	2,00	1	1,00	0,00
1000,00	1	0	0	0	0,00	0	0,00	1,00
1000,00	1	0	0	0	0,00	0	1,00	0,00
1000,00	1	0	0	0	0,00	0	0,00	1,00
1000,00	1	0	0	0	0,00	0	1,00	0,00
1000,00	1	0	0	0	0,00	0	1,00	0,00
1000,00	1	0	0	0	0,00	0	0,00	1,00
2,00	1	1	1	1	3,00	1	1,00	0,00
1000,00	1	0	0	0	0,00	0	1,00	0,00
1,00	1	0	0	1	1,00	1	1,00	0,00
1000,00	1	0	#NULL!	0	#NULL!	#NULL!	1,00	0,00
1,00	1	0	0	1	1,00	1	1,00	0,00
1000,00	1	#NULL!	#NULL!	#NULL!	#NULL!	#NULL!	0,00	0,00
1000,00	1	0	0	0	0,00	0	0,00	1,00
1000,00	1	0	0	0	0,00	0	1,00	0,00
1000,00	1	0	0	0	0,00	0	0,00	0,00
1000,00	1	0	0	0	0,00	0	1,00	0,00
1,00	1	0	0	1	1,00	1	1,00	0,00
1,00	1	0	1	1	2,00	1	0,00	1,00

1000,00	1	0	0	0	0,00	0	1,00	0,00
1000,00	1	0	1	0	1,00	1	1,00	0,00
1000,00	1	0	0	0	0,00	0	1,00	0,00
1,00	1	0	1	1	2,00	1	0,00	1,00
1000,00	1	0	0	0	0,00	0	1,00	0,00
1,00	1	0	1	1	2,00	1	0,00	1,00
1000,00	1	0	0	0	0,00	0	1,00	0,00
1000,00	1	0	0	0	0,00	0	1,00	0,00
1000,00	1	0	0	0	0,00	0	1,00	0,00
1000,00	1	0	0	0	0,00	0	0,00	1,00
1000,00	1	0	0	0	0,00	0	1,00	0,00
1000,00	1	0	0	0	0,00	0	1,00	0,00
1000,00	1	0	0	0	0,00	0	1,00	0,00
1,00	1	0	1	1	2,00	1	1,00	0,00
1,00	1	0	0	1	1,00	1	1,00	0,00
1000,00	1	0	0	0	0,00	0	1,00	0,00
1000,00	1	0	0	0	0,00	0	1,00	0,00
1,00	1	0	1	1	2,00	1	1,00	0,00
2,00	1	0	0	1	1,00	1	1,00	0,00
1,00	1	0	0	1	1,00	1	1,00	0,00
1,00	1	0	0	1	1,00	1	1,00	0,00
1000,00	1	0	0	0	0,00	0	1,00	0,00
1000,00	1	0	0	0	0,00	0	1,00	0,00
1,00	1	0	1	1	2,00	1	0,00	1,00
2,00	1	0	1	1	2,00	1	1,00	0,00
1,00	1	1	0	1	2,00	1	1,00	0,00
1,00	1	1	1	1	3,00	1	1,00	0,00
1000,00	1	0	0	0	0,00	0	1,00	0,00
1,00	1	0	1	1	2,00	1	1,00	0,00
1,00	1	0	1	1	2,00	1	1,00	0,00
1000,00	1	1	0	0	1,00	1	0,00	1,00
1,00	1	0	0	1	1,00	1	0,00	1,00
1,00	1	1	1	1	3,00	1	0,00	1,00
2,00	1	0	1	1	2,00	1	0,00	1,00
1,00	1	1	1	1	3,00	1	1,00	0,00
1000,00	1	0	0	0	0,00	0	1,00	0,00
1,00	1	0	0	1	1,00	1	0,00	1,00
1000,00	1	0	0	0	0,00	0	1,00	0,00
1,00	1	0	1	1	2,00	1	0,00	0,00
1,00	1	1	1	1	3,00	1	1,00	0,00
1,00	1	1	0	1	2,00	1	0,00	1,00
2,00	1	1	0	1	2,00	1	1,00	0,00
2,00	1	0	1	1	2,00	1	1,00	0,00
1000,00	1	0	0	0	0,00	0	1,00	0,00
1000,00	1	0	0	0	0,00	0	1,00	0,00
1000,00	1	0	0	0	0,00	0	1,00	0,00
1000,00	1	0	0	0	0,00	0	1,00	0,00
2,00	1	1	1	1	3,00	1	0,00	1,00
1,00	1	0	0	1	1,00	1	1,00	0,00
1,00	1	0	0	1	1,00	1	1,00	0,00

2,00	1	0	0	1	1,00	1	1,00	0,00
1,00	1	1	0	1	2,00	1	0,00	1,00
1,00	1	1	1	1	3,00	1	0,00	1,00
1000,00	1	0	0	0	0,00	0	1,00	0,00
1000,00	1	0	0	0	0,00	0	1,00	0,00
1000,00	1	0	0	0	0,00	0	1,00	0,00
1000,00	1	0	0	0	0,00	0	1,00	0,00
2,00	1	0	0	1	1,00	1	1,00	0,00
1000,00	1	0	0	0	0,00	0	1,00	0,00
1,00	1	0	0	1	1,00	1	1,00	0,00
99,00	1	0	0	1	1,00	1	1,00	0,00
2,00	1	0	1	1	2,00	1	0,00	1,00
1,00	1	0	0	1	1,00	1	1,00	0,00
2,00	1	1	1	1	3,00	1	0,00	1,00
1,00	1	0	1	1	2,00	1	1,00	0,00
1,00	1	1	1	1	3,00	1	0,00	1,00
1000,00	1	0	0	0	0,00	0	1,00	0,00
1000,00	1	0	0	0	0,00	0	1,00	0,00
1,00	1	1	0	1	2,00	1	1,00	0,00
1,00	1	1	1	1	3,00	1	0,00	1,00
1000,00	1	1	0	0	1,00	1	1,00	0,00
1,00	1	0	1	1	2,00	1	0,00	1,00
1,00	1	0	0	1	1,00	1	1,00	0,00
1,00	1	0	0	1	1,00	1	0,00	0,00
1,00	1	1	1	1	3,00	1	1,00	0,00
1000,00	1	0	1	1	2,00	1	1,00	0,00
1,00	1	0	0	1	1,00	1	1,00	0,00
1000,00	1	0	0	0	0,00	0	1,00	0,00
1,00	1	0	0	1	1,00	1	1,00	0,00
1,00	1	0	0	1	1,00	1	1,00	0,00
1000,00	1	0	0	0	0,00	0	1,00	0,00
1000,00	1	0	0	0	0,00	0	1,00	0,00

NEW_AGE	NEW_PART	Nrelationsh	Newrelatio	twelvemon	twelvesexu	Twelveemo	Totalabuse12
2,00	0,00	1 married	1 currently	2Nophysica	Noexualab	No emotio	0,00
2,00	0,00	1 married	1 currently	2Nophysica	Noexualab	No emotio	0,00
2,00	0,00	1 married	1 currently	2Nophysica	Noexualab	No emotio	0,00
2,00	1,00	1 married	1 currently with				0,00
2,00	1,00	1 married	1 currently with		Twelve mo	Emotional	2,00
2,00	0,00	1 married	1 currently with				0,00
2,00	0,00	1 married	1 currently with		Twelve mo	Emotional	2,00
1,00	1,00	1 married	1 currently with		Twelve mo	Emotional	2,00
1,00	0,00	2 current p	1 currently with		Noexualab	Emotional	1,00
1,00	0,00	1 married	1 currently	2Nophysica	Noexualab	Emotional	1,00
1,00	0,00	1 married	1 currently with			Emotional	1,00
1,00	0,00	1 married	1 currently with		Noexualab	No emotio	0,00
1,00	0,00	1 married	1 currently	2Nophysical twel			0,00
2,00	0,00	1 married	1 currently	2Nophysica	Twelve mo	No emotio	1,00
1,00	1,00	2 current p	2 no currer	2Nophysica	Noexualab	No emotio	0,00
1,00	0,00	1 married	1 currently with				0,00
1,00	0,00	1 married	1 currently with		Twelve mo	Emotional	2,00
1,00	0,00	1 married	1 currently with			Emotional	1,00
1,00	0,00	1 married	1 currently	2Nophysical twel			0,00
2,00	0,00	1 married	1 currently	1 twelve m	Noexualab	Emotional	2,00
2,00	1,00	1 married	1 currently	1 twelve m	Noexualab	Emotional	2,00
2,00	0,00	1 married	1 currently	1 twelve m	Twelve mo	Emotional	3,00
2,00	1,00	2 current p	1 currently with		Twelve months ab		1,00
2,00	1,00	1 married	1 currently with		Twelve mo	Emotional	2,00
2,00	1,00	1 married	1 currently with		Noexualab	No emotio	0,00
2,00	0,00	1 married	1 currently with		Twelve mo	Emotional	2,00
2,00	0,00	1 married	1 currently with				0,00
2,00	0,00	1 married	1 currently with				0,00
2,00	0,00	1 married	1 currently	1 twelve m	Twelve mo	No emotio	2,00
2,00	1,00	1 married	1 currently with		Twelve mo	Emotional	2,00
2,00	0,00	1 married	1 currently with			No emotio	0,00
2,00	0,00	1 married	1 currently with			Emotional	1,00
2,00	1,00	2 current p	1 currently	1 twelve m	Twelve mo	Emotional	3,00
2,00	1,00	2 current p	2 no currer	2Nophysica	Noexualab	No emotio	0,00
2,00	0,00	1 married	1 currently with		Noexualab	Emotional	1,00
2,00	0,00	1 married	1 currently	2Nophysica	Noexualab	No emotio	0,00
2,00	1,00	1 married	1 currently	2Nophysical twel		No emotio	0,00
2,00	0,00	1 married	1 currently with				0,00
2,00	1,00	1 married	1 currently with		Twelve mo	Emotional	2,00
2,00	1,00	1 married	1 currently	2Nophysica	Noexualab	Emotional	1,00
2,00	1,00	2 current p	1 currently with				0,00
2,00	0,00	1 married	1 currently with		Twelve mo	Emotional	2,00
2,00	0,00	1 married	1 currently	1 twelve m	Twelve mo	Emotional	3,00
2,00	0,00	1 married	1 currently with			No emotio	0,00
2,00	1,00	1 married	1 currently with		Noexualab	Emotional	1,00
2,00	0,00	1 married	1 currently with				0,00
1,00	0,00	1 married	1 currently	1 twelve m	Twelve mo	Emotional	3,00
1,00	0,00	1 married	1 currently with		Twelve mo	Emotional	2,00
1,00	0,00	1 married	1 currently with			Emotional	1,00

1,00	0,00	1 married	1 currently with		Twelve mo	No emotio	1,00
1,00	0,00	2 current p	1 currently with				0,00
1,00	0,00	1 married	1 currently with		Twelve months ab		1,00
1,00	1,00	1 married	1 currently with				0,00
1,00	0,00	1 married	1 currently with			No emotio	0,00
2,00	0,00	1 married	1 currently	1 twelve m	Twelve months ab		2,00
2,00	0,00	1 married	1 currently	2Nophysical twel		No emotio	0,00
1,00	0,00	2 current p	1 currently with			Emotional	1,00
1,00	0,00	1 married	1 currently	1 twelve months		Emotional	2,00
1,00	0,00	1 married	1 currently with				0,00
1,00	0,00	1 married	1 currently with			Emotional	1,00
1,00	1,00	1 married	1 currently with			No emotio	0,00
1,00	0,00	2 current p	1 currently with			No emotio	0,00
1,00	0,00	2 current p	1 currently	1 twelve m	Noexualab	Emotional	2,00
1,00	0,00	1 married	1 currently with			Emotional	1,00
1,00	0,00	2 current p	1 currently	1 twelve months			1,00
1,00	1,00	2 current p	1 currently	1 twelve m	Noexualab	Emotional	2,00
1,00	0,00	1 married	1 currently with				0,00
1,00	0,00	2 current p	1 currently with				0,00
1,00	1,00	2 current p	1 currently	2Nophysica	Noexualab	No emotio	0,00
1,00	0,00	1 married	1 currently with				0,00
1,00	1,00	1 married	1 currently	2Nophysica	Noexualab	No emotio	0,00
1,00	0,00	2 current p	1 currently with				0,00
1,00	1,00	2 current p	1 currently	1 twelve m	Noexualabuse twe		1,00
1,00	0,00	2 current p	1 currently with				0,00
1,00	1,00	1 married	1 currently with				0,00
1,00	0,00	1 married	1 currently	2Nophysical twel		No emotio	0,00
1,00	0,00	2 current p	1 currently with			No emotio	0,00
1,00	0,00	1 married	1 currently	2Nophysica	Noexualab	Emotional	1,00
1,00	1,00	1 married	1 currently	2Nophysica	Twelve mo	Emotional	2,00
1,00	0,00	1 married	1 currently with			Emotional	1,00
1,00	0,00	1 married	1 currently with				0,00
1,00	1,00	1 married	1 currently with			Emotional	1,00
1,00	1,00	1 married	1 currently	2Nophysica	Noexualab	Emotional	1,00
1,00	0,00	2 current p	1 currently	1 twelve months		Emotional	2,00
1,00	0,00	1 married	1 currently with				0,00
1,00	0,00	1 married	1 currently	1 twelve months		Emotional	2,00
1,00	0,00	1 married	1 currently with			Emotional	1,00
1,00	0,00	1 married	1 currently with		Twelve mo	Emotional	2,00
1,00	1,00	1 married	1 currently	1 twelve months		No emotio	1,00
1,00	0,00	1 married	1 currently with			Emotional	1,00
1,00	0,00	1 married	1 currently with			No emotio	0,00
1,00	1,00	1 married	1 currently with			Emotional	1,00
1,00	1,00	2 current p	2 no current par				0,00
1,00	0,00	1 married	1 currently with			No emotio	0,00
1,00	0,00	1 married	1 currently	2Nophysical twel		No emotio	0,00
1,00	0,00	1 married	1 currently with				0,00
1,00	0,00	1 married	1 currently	1 twelve months		Emotional	2,00
1,00	0,00	2 current p	2 no current par				0,00
1,00	0,00	1 married	1 currently	2Nophysical twel		Emotional	1,00

1,00	0,00	1 married	1 currently with				0,00
1,00	0,00	2 current p	1 currently with			No emotioı	0,00
1,00	1,00	1 married	1 currently with		Noexualabuse twe		0,00
1,00	0,00	2 current p	1 currently with				0,00
1,00	0,00	1 married	1 currently with			Emotional	1,00
1,00	0,00	1 married	1 currently with				0,00
1,00	0,00	1 married	1 currently with				0,00
1,00	0,00	2 current p	1 currently with				0,00
1,00	0,00	2 current p	1 currently with			Emotional	1,00
1,00	1,00	1 married	1 currently with				0,00
1,00	0,00	2 current p	1 currently with			Emotional	1,00
1,00	0,00	1 married	1 currently with			No emotioı	0,00
1,00	1,00	1 married	1 currently with			Emotional	1,00
1,00	0,00	2 current p	1 currently	1 twelve m	Twelve mo	Emotional	3,00
1,00	1,00	1 married	1 currently with		Twelve mo	Emotional	2,00
1,00	0,00	1 married	1 currently with				0,00
1,00	0,00	1 married	1 currently with			No emotioı	0,00
1,00	0,00	1 married	1 currently with				0,00
2,00	1,00	2 current p	1 currently with				0,00
2,00	0,00	1 married	1 currently with				0,00
2,00	0,00	1 married	1 currently with				0,00
2,00	1,00	1 married	1 currently with			No emotioı	0,00
2,00	0,00	1 married	1 currently with				0,00
2,00	0,00	2 current p	1 currently with				0,00
2,00	1,00	1 married	1 currently	2Nophysical twel		No emotioı	0,00
2,00	1,00	1 married	1 currently with				0,00
2,00	1,00	1 married	1 currently	1 twelve m	Twelve mo	Emotional	3,00
2,00	1,00	1 married	1 currently	2Nophysica	Noexualabı	Emotional	1,00
2,00	0,00	1 married	1 currently with				0,00
1,00	0,00	2 current p	1 currently with				0,00
1,00	0,00	2 current p	1 currently with		Twelve months ab		1,00
1,00	0,00	2 current p	1 currently	1 twelve months			1,00
1,00	0,00	2 current p	1 currently with		Noexualabı	No emotioı	0,00
1,00	0,00	2 current p	1 currently with				0,00
1,00	0,00	2 current p	1 currently with				0,00
1,00	0,00	2 current p	1 currently with		Noexualabuse twe		0,00
1,00	0,00	1 married	1 currently with			No emotioı	0,00
1,00	1,00	2 current p	1 currently with			No emotioı	0,00
1,00	0,00	1 married	1 currently with			No emotioı	0,00
1,00	0,00	1 married	1 currently with				0,00
1,00	0,00	1 married	1 currently with		Noexualabı	No emotioı	0,00
1,00	0,00	2 current p	1 currently with				0,00
1,00	0,00	1 married	1 currently with				0,00
1,00	0,00	1 married	1 currently with				0,00
1,00	0,00	1 married	1 currently with		Twelve mo	Emotional	2,00
1,00	0,00	1 married	1 currently with			Emotional	1,00
1,00	1,00	1 married	1 currently with		Twelve mo	Emotional	2,00
1,00	0,00	2 current p	1 currently with				0,00
1,00	1,00	1 married	1 currently	1 twelve m	Twelve mo	Emotional	3,00
1,00	0,00	1 married	1 currently with				0,00

1,00	1,00	1 married	1 currently with		Twelve mo	Emotional	2,00
1,00	1,00	2 current p	1 currently with				0,00
1,00	0,00	1 married	1 currently	1 twelve m	Twelve mo	Emotional	3,00
1,00	0,00	2 current p	1 currently with				0,00
1,00	0,00	2 current p	1 currently with			Emotional	1,00
1,00	1,00	2 current p	1 currently with			Emotional	1,00
1,00	1,00	1 married	1 currently with				0,00
1,00	1,00	2 current p	1 currently with		Twelve mo	Emotional	2,00
1,00	0,00	2 current p	1 currently with				0,00
1,00	1,00	1 married	1 currently with			No emotio	0,00
1,00	0,00	2 current p	1 currently	1 twelve m	Noexualab	Emotional	2,00
1,00	1,00	1 married	1 currently with				0,00
1,00	0,00	2 current p	1 currently with				0,00
1,00	0,00	1 married	1 currently with			Emotional	1,00
1,00	0,00	1 married	1 currently with				0,00
1,00	1,00	1 married	1 currently with				0,00
1,00	0,00	2 current p	1 currently with				0,00
1,00	1,00	2 current p	1 currently with				0,00
1,00	0,00	2 current p	1 currently with				0,00
1,00	0,00	2 current p	1 currently with				0,00
1,00	0,00	1 married	1 currently with				0,00
1,00	0,00	2 current p	1 currently with				0,00
1,00	1,00	1 married	1 currently	2Nophysical twel		No emotio	0,00
1,00	1,00	2 current p	1 currently	2Nophysical twel		No emotio	0,00
1,00	1,00	2 current p	1 currently with			No emotio	0,00
1,00	1,00	1 married	1 currently with				0,00
1,00	1,00	1 married	1 currently with				0,00
1,00	0,00	2 current p	1 currently with				0,00
1,00	1,00	1 married	1 currently	1 twelve m	Twelve mo	Emotional	3,00
1,00	0,00	2 current p	1 currently with				0,00
1,00	0,00	1 married	1 currently with			No emotio	0,00
1,00	0,00	1 married	1 currently with			No emotio	0,00
1,00	0,00	2 current p	1 currently with				0,00
1,00	0,00	1 married	1 currently	2Nophysica	Noexualab	No emotio	0,00
1,00	0,00	1 married	1 currently with				0,00
1,00	0,00	1 married	1 currently with			Emotional	1,00
1,00	0,00	1 married	1 currently with			Emotional	1,00
1,00	1,00	1 married	1 currently with				0,00
1,00	0,00	2 current p	1 currently with				0,00
1,00	0,00	1 married	1 currently with		Noexualab	Emotional	1,00
1,00	0,00	1 married	1 currently with			Emotional	1,00
1,00	0,00	2 current p	2 no current par			No emotio	0,00
1,00	0,00	2 current p	2 no current par			Emotional	1,00
1,00	1,00	2 current p	1 currently with			Emotional	1,00
1,00	0,00	1 married	1 currently	1 twelve months			1,00
1,00	1,00	1 married	1 currently with				0,00
1,00	0,00	1 married	1 currently with				0,00
1,00	0,00	1 married	1 currently with				0,00
1,00	0,00	1 married	1 currently with				0,00
1,00	0,00	1 married	1 currently with				0,00

1,00	0,00	1 married	1 currently with				0,00
1,00	0,00	2 current p	1 currently with				0,00
1,00	1,00	2 current p	2 no current par				0,00
1,00	0,00	1 married	1 currently with				0,00
1,00	0,00	1 married	1 currently with			Emotional	1,00
1,00	0,00	1 married	1 currently with			Emotional	1,00
1,00	0,00	1 married	1 currently with			Emotional	1,00
1,00	0,00	1 married	1 currently	1 twelve months		Emotional	2,00
1,00	0,00	2 current p	2 no current par				0,00
1,00	0,00	2 current p	2 no current par				0,00
1,00	0,00	1 married	1 currently with			Emotional	1,00
1,00	1,00	2 current p	1 currently with				0,00
1,00	1,00	2 current p	2 no current par				0,00
1,00	0,00	2 current p	1 currently with				0,00
1,00	0,00	1 married	1 currently with				0,00
1,00	0,00	2 current p	1 currently with		Twelve months ab		1,00
1,00	1,00	2 current p	1 currently with				0,00
1,00	0,00	2 current p	1 currently	1 twelve m	Twelve mo	Emotional	3,00
1,00	0,00	2 current p	1 currently with				0,00
1,00	0,00	2 current p	1 currently with			Emotional	1,00
1,00	1,00	2 current p	2 no current par				0,00
1,00	1,00	2 current p	2 no current par			Emotional	1,00
1,00	0,00	1 married	1 currently with			Emotional	1,00
1,00	0,00	1 married	1 currently	1 twelve m	Twelve mo	Emotional	3,00
1,00	1,00	2 current p	1 currently with		Noexualab	No emotio	0,00
1,00	1,00	1 married	1 currently with			Emotional	1,00
1,00	0,00	1 married	1 currently with				0,00
1,00	0,00	2 current p	1 currently	1 twelve m	Twelve mo	Emotional	3,00
1,00	0,00	1 married	1 currently	1 twelve months		Emotional	2,00
1,00	1,00	2 current p	1 currently with				0,00
1,00	0,00	1 married	1 currently with			Emotional	1,00
1,00	1,00	1 married	1 currently with				0,00
1,00	0,00	2 current p	1 currently with		Twelve mo	Emotional	2,00
1,00	0,00	1 married	1 currently	1 twelve months		Emotional	2,00
1,00	0,00	1 married	1 currently	1 twelve m	Twelve mo	Emotional	3,00
1,00	1,00	1 married	1 currently	1 twelve m	Twelve mo	Emotional	3,00
2,00	0,00	1 married	1 currently with			Emotional	1,00
2,00	0,00	2 current p	1 currently with			No emotio	0,00
2,00	0,00	2 current p	1 currently	2Nophysica	Noexualab	No emotio	0,00
2,00	0,00	1 married	1 currently with			Emotional	1,00
2,00	0,00	1 married	1 currently	2Nophysica	Noexualab	Emotional	1,00
2,00	0,00	1 married	1 currently	1 twelve m	Twelve mo	Emotional	3,00
2,00	0,00	1 married	1 currently with		Twelve mo	Emotional	2,00
2,00	0,00	1 married	1 currently with			Emotional	1,00
2,00	0,00	1 married	1 currently with				0,00
2,00	0,00	1 married	1 currently with				0,00
2,00	0,00	1 married	1 currently with			Emotional	1,00
1,00	0,00	1 married	1 currently with				0,00
1,00	0,00	1 married	1 currently with		Twelve mo	Emotional	2,00
1,00	0,00	1 married	1 currently with		Twelve mo	Emotional	2,00

1,00	0,00	1 married	1 currently with			Emotional	1,00
1,00	1,00	1 married	1 currently with			Emotional	1,00
1,00	0,00	1 married	1 currently with				0,00
1,00	1,00	1 married	1 currently with				0,00
1,00	0,00	1 married	1 currently with				0,00
1,00	1,00	1 married	1 currently with		Twelve mo	Emotional	2,00
1,00	1,00	2 current p	1 currently with		Noexualab	Emotional	1,00
1,00	0,00	1 married	1 currently with		Twelve months ab		1,00
1,00	1,00	1 married	1 currently with			Emotional	1,00
1,00	0,00	1 married	1 currently with		Twelve mo	Emotional	2,00
1,00	0,00	1 married	1 currently	1 twelve months		Emotional	2,00
1,00	0,00	2 current p	1 currently with			No emotio	0,00
1,00	0,00	2 current p	1 currently with				0,00
1,00	1,00	1 married	1 currently	1 twelve m	Twelve months ab		2,00
1,00	0,00	2 current p	1 currently with		Twelve mo	Emotional	2,00
1,00	0,00	2 current p	1 currently with		Noexualab	No emotio	0,00
1,00	0,00	1 married	1 currently with			Emotional	1,00
1,00	0,00	2 current p	1 currently with				0,00
1,00	1,00	2 current p	1 currently with				0,00
1,00	0,00	2 current p	1 currently with		Noexualab	No emotio	0,00
1,00	0,00	2 current p	1 currently with			Emotional	1,00
1,00	0,00	2 current p	1 currently with				0,00
1,00	0,00	2 current p	1 currently	1 twelve m	Noexualab	No emotio	1,00
1,00	0,00	2 current p	1 currently with				0,00
1,00	0,00	2 current p	1 currently with				0,00
1,00	1,00	2 current p	1 currently with				0,00
1,00	0,00	2 current p	1 currently with				0,00
1,00	0,00	2 current p	1 currently with				0,00
1,00	0,00	1 married	1 currently with				0,00
1,00	0,00	2 current p	1 currently	2Nophysica	Twelve mo	Emotional	2,00
1,00	0,00	1 married	1 currently with				0,00
1,00	0,00	1 married	1 currently with				0,00
1,00	0,00	1 married	1 currently with				0,00
1,00	0,00	1 married	1 currently with				0,00
1,00	0,00	1 married	1 currently with			Emotional	1,00
1,00	0,00	1 married	1 currently	1 twelve months			1,00
1,00	1,00	2 current p	1 currently with				0,00
1,00	0,00	2 current p	1 currently with			Emotional	1,00
1,00	0,00	1 married	1 currently	1 twelve m	Twelve mo	Emotional	3,00
1,00	0,00	2 current p	1 currently with				0,00
1,00	0,00	1 married	1 currently with			Emotional	1,00
1,00	0,00	1 married	1 currently with			No emotio	0,00
1,00	0,00	1 married	1 currently with				0,00
1,00	0,00	1 married	1 currently with				0,00
1,00	0,00	2 current p	1 currently with			Emotional	1,00
1,00	0,00	1 married	1 currently with				0,00
1,00	0,00	1 married	1 currently	1 twelve months		Emotional	2,00
1,00	0,00	1 married	1 currently with			Emotional	1,00
1,00	0,00	1 married	1 currently with				0,00
1,00	0,00	2 current p	1 currently with				0,00

1,00	0,00	2 current p	1 currently with				0,00
1,00	0,00	2 current p	1 currently with		Twelve mo	Emotional	2,00
1,00	0,00	2 current p	1 currently	1 twelve months			1,00
1,00	1,00	1 married	1 currently	2Nophysical twel		No emotio	0,00
1,00	1,00	1 married	1 currently with			Emotional	1,00
1,00	0,00	1 married	1 currently with				0,00
1,00	1,00	2 current p	1 currently with				0,00
1,00	0,00	2 current p	1 currently	2Nophysical twel			0,00
1,00	0,00	2 current p	1 currently with			No emotio	0,00
1,00	0,00	2 current p	1 currently with				0,00
1,00	0,00	1 married	1 currently with				0,00
1,00	0,00	2 current p	1 currently with				0,00
1,00	1,00	2 current p	1 currently with				0,00
1,00	1,00	2 current p	1 currently with				0,00
1,00	0,00	2 current p	1 currently with			No emotio	0,00
1,00	0,00	2 current p	1 currently with				0,00
1,00	1,00	1 married	1 currently with		Twelve mo	Emotional	2,00
1,00	0,00	2 current p	1 currently with				0,00
1,00	0,00	2 current p	1 currently with				0,00
1,00	1,00	2 current p	1 currently with				0,00
1,00	0,00	1 married	1 currently	2Nophysical twel		Emotional	1,00
1,00	1,00	1 married	1 currently with				0,00
1,00	1,00	2 current p	1 currently with				0,00
1,00	0,00	1 married	1 currently with				0,00
1,00	0,00	2 current p	1 currently with				0,00
1,00	1,00	2 current p	2 no current par				0,00
1,00	0,00	1 married	1 currently	1 twelve months		Emotional	2,00
1,00	1,00	1 married	1 currently with			Emotional	1,00
1,00	1,00	1 married	1 currently	1 twelve months		Emotional	2,00
1,00	1,00	1 married	1 currently	1 twelve months		Emotional	2,00
1,00	0,00	2 current p	1 currently with			Emotional	1,00
1,00	0,00	1 married	1 currently	2Nophysical twel		No emotio	0,00
1,00	1,00	2 current p	1 currently with				0,00
1,00	0,00	1 married	1 currently with				0,00
1,00	1,00	2 current p	1 currently with				0,00
1,00	0,00	2 current p	1 currently with				0,00
1,00	0,00	2 current p	1 currently with				0,00
1,00	1,00	1 married	1 currently with				0,00
1,00	0,00	1 married	1 currently	2Nophysica	Noexualab	No emotio	0,00
1,00	0,00	1 married	1 currently with				0,00
1,00	0,00	1 married	1 currently with			Emotional	1,00
1,00	0,00	2 current p	1 currently with				0,00
1,00	0,00	2 current p	1 currently with			Emotional	1,00
1,00	#NULL!	2 current p	1 currently with				0,00
1,00	1,00	1 married	1 currently with				0,00
1,00	0,00	1 married	1 currently with				0,00
1,00	1,00	1 married	1 currently with				0,00
1,00	0,00	1 married	1 currently with				0,00
1,00	0,00	2 current p	1 currently with			Emotional	1,00
1,00	1,00	2 current p	1 currently with		Twelve mo	Emotional	2,00

1,00	0,00	1 married	1 currently with				0,00
1,00	0,00	2 current p	1 currently with		Twelve months ab		1,00
1,00	0,00	1 married	1 currently with				0,00
1,00	1,00	1 married	1 currently with		Twelve mo	Emotional	2,00
1,00	0,00	2 current p	1 currently with				0,00
1,00	1,00	1 married	1 currently with		Twelve mo	Emotional	2,00
1,00	0,00	1 married	1 currently with				0,00
1,00	0,00	2 current p	1 currently with				0,00
1,00	0,00	2 current p	1 currently with				0,00
1,00	1,00	1 married	1 currently with				0,00
1,00	0,00	1 married	1 currently with				0,00
1,00	0,00	1 married	1 currently with				0,00
1,00	0,00	2 current p	1 currently with				0,00
1,00	0,00	1 married	1 currently with		Twelve mo	Emotional	2,00
1,00	0,00	1 married	1 currently with			Emotional	1,00
1,00	0,00	1 married	1 currently with				0,00
1,00	0,00	1 married	1 currently with				0,00
1,00	0,00	2 current p	2 no current par		Twelve mo	Emotional	2,00
1,00	0,00	2 current p	1 currently with			No emotio	0,00
1,00	0,00	1 married	1 currently with			Emotional	1,00
1,00	0,00	2 current p	1 currently with			Emotional	1,00
1,00	0,00	1 married	1 currently with				0,00
1,00	0,00	2 current p	1 currently with				0,00
1,00	1,00	1 married	1 currently with		Twelve mo	Emotional	2,00
1,00	0,00	1 married	1 currently with		Noexualab	No emotio	0,00
1,00	0,00	1 married	1 currently	1 twelve months		Emotional	2,00
1,00	0,00	1 married	1 currently	1 twelve m	Twelve mo	Emotional	3,00
1,00	0,00	1 married	1 currently with				0,00
1,00	0,00	1 married	1 currently with		Noexualab	Emotional	1,00
1,00	0,00	1 married	1 currently with		Noexualab	Emotional	1,00
1,00	1,00	1 married	1 currently	2Nophysical twel			0,00
1,00	1,00	1 married	1 currently with			Emotional	1,00
1,00	1,00	1 married	1 currently	1 twelve m	Noexualab	Emotional	2,00
1,00	1,00	1 married	1 currently with		Noexualab	No emotio	0,00
1,00	0,00	1 married	1 currently	1 twelve m	Twelve mo	Emotional	3,00
1,00	0,00	2 current p	1 currently with				0,00
1,00	1,00	1 married	1 currently with			Emotional	1,00
1,00	0,00	1 married	1 currently with				0,00
1,00	1,00	2 current p	1 currently with		Twelve mo	Emotional	2,00
1,00	0,00	1 married	1 currently	1 twelve m	Twelve mo	Emotional	3,00
1,00	1,00	1 married	1 currently	1 twelve months		Emotional	2,00
1,00	0,00	1 married	1 currently	1 twelve months		No emotio	1,00
1,00	0,00	2 current p	1 currently with		Noexualab	No emotio	0,00
1,00	0,00	1 married	1 currently with				0,00
1,00	0,00	1 married	1 currently with				0,00
1,00	0,00	1 married	1 currently with				0,00
1,00	0,00	2 current p	1 currently with				0,00
1,00	1,00	1 married	1 currently	2Nophysica	Noexualab	No emotio	0,00
1,00	0,00	2 current p	1 currently with			Emotional	1,00
1,00	0,00	2 current p	1 currently with			Emotional	1,00

1,00	0,00	1 married	1 currently with			No emotio	0,00
1,00	1,00	1 married	1 currently	2Nophysical twel		Emotional	1,00
1,00	1,00	1 married	1 currently	2Nophysica	Twelve mo	Emotional	2,00
1,00	0,00	1 married	1 currently with				0,00
1,00	0,00	1 married	1 currently with				0,00
1,00	0,00	1 married	1 currently with				0,00
1,00	0,00	2 current p	1 currently with				0,00
1,00	0,00	1 married	1 currently with			No emotio	0,00
1,00	0,00	1 married	1 currently with				0,00
1,00	0,00	1 married	1 currently with			Emotional	1,00
1,00	0,00	1 married	1 currently with				0,00
1,00	1,00	1 married	1 currently with		Noexualab	No emotio	0,00
1,00	0,00	1 married	1 currently with			Emotional	1,00
1,00	1,00	1 married	1 currently	1 twelve m	Noexualab	No emotio	1,00
1,00	0,00	1 married	1 currently with		Twelve mo	Emotional	2,00
1,00	1,00	2 current p	1 currently with		Twelve mo	Emotional	2,00
2,00	0,00	2 current p	2 no current par				0,00
2,00	0,00	1 married	1 currently with				0,00
2,00	0,00	2 current p	1 currently	1 twelve months		Emotional	2,00
2,00	1,00	1 married	1 currently	1 twelve m	Twelve mo	Emotional	3,00
1,00	0,00	1 married	1 currently	2Nophysical twel			0,00
1,00	1,00	1 married	1 currently with		Twelve mo	Emotional	2,00
1,00	0,00	1 married	1 currently with			Emotional	1,00
1,00	1,00	1 married	1 currently with			Emotional	1,00
1,00	0,00	1 married	1 currently	1 twelve m	Twelve mo	Emotional	3,00
1,00	0,00	1 married	1 currently with		Twelve months ab		1,00
1,00	0,00	2 current p	1 currently with			Emotional	1,00
1,00	0,00	2 current p	1 currently with				0,00
1,00	0,00	2 current p	1 currently with			Emotional	1,00
1,00	0,00	1 married	1 currently with			Emotional	1,00
1,00	0,00	1 married	1 currently with				0,00
1,00	0,00	1 married	1 currently with				0,00
